# Evaluating the impact of sling provision and training upon maternal mental health, wellbeing and parenting: A randomised feasibility trial

**DOI:** 10.1371/journal.pone.0293501

**Published:** 2023-11-10

**Authors:** Helen Wigglesworth, Vyv Huddy, Rosie Knowles, Abigail Millings

**Affiliations:** 1 Sheffield Children’s NHS FT, Sheffield, United Kingdom; 2 Clinical Psychology Unit, University of Sheffield, Sheffield, United Kingdom; 3 Sheffield Sling Surgery and Library, Carrying Matters, Sheffield, United Kingdom; 4 Centre for Behavioural Science and Applied Psychology, Sheffield Hallam University, Sheffield, United Kingdom; PLOS: Public Library of Science, UNITED KINGDOM

## Abstract

**Background:**

Close body contact interventions such as Kangaroo Mother Care have been shown to improve maternal mental health following birth. Infant carriers (‘slings’) facilitate hands-free close body contact. No studies have specifically examined whether sling use improves maternal mental health. A full-scale efficacy study is needed to examine whether sling use is beneficial to maternal mental health. The current study is a feasibility study designed to gather information to support the design of a future RCT, such as acceptability and study parameters, including recruitment rates, consent rate and attrition.

**Method:**

Mothers of infants aged 0–6 weeks were randomised to one of two conditions: intervention (*n* = 35) vs. waitlist control (*n* = 32). Intervention participants received sling training, support, and free sling hire for 12 weeks. Participants completed self-report measures of mood, wellbeing and parenting at baseline (Time 1), and 6- (Time 2) and 12- (Time 3) weeks post-baseline.

**Results:**

Eligibility and consent rates met feasibility objectives, though there were some difficulties with retention of participants in the study. Preliminary effectiveness analyses showed a non-significant improvement with a small effect size in postnatal depression from T1 to T3, and a significant improvement with a medium effect size in maternal self-efficacy from T1 to T3. Qualitative feedback indicated acceptability of the intervention and study participation. Intervention participants attributed greater autonomy, bonding with their baby, and parental self-confidence, to the intervention.

**Conclusions:**

These findings indicate a randomised study of the impact of a sling and related support intervention upon maternal mental health is feasible. These findings should be interpreted within the context of sampling bias (due to the use of volunteer sampling methods), an absence of feedback from those who discontinued participation in the study, and the study not being adequately powered.

**Trial registration:**

Registration number ISRCTN88575352.

## Introduction

Women commonly experience both physical and psychological difficulties following childbirth [1]. Worldwide, 10–15% of mothers suffer from postnatal depression (PND) [2]. A greater percentage of mothers (around 30%) may experience subthreshold depressive symptoms following childbirth [3]. There is a need for low intensity interventions to mitigate these symptoms, and support mothers’ well-being.

For those with mild to moderate symptoms of PND, National Institute for health and Care Excellence (NICE) guidelines recommend guided self-help, cognitive behaviour therapy or exercise programmes [4, 5]. These low-level interventions can be onerous for mothers to access soon after giving birth [6], yet without early intervention, PND symptoms can worsen [3].

Evidence is emerging for alternative early intervention methods, including psycho-educational home visits [7], parenting groups [8], and baby massage [9], but these are not yet well- supported.

A low cost and low intensity intervention, known to have beneficial effects on both maternal and infant wellbeing, is close body contact [10]. Skin-to-skin contact (SSC) and Kangaroo Mother Care (KMC) both involve placing the infant upon the mother’s chest in a vertical position, dressed only in a nappy, so that mother and infant frontal body contact is skin-to-skin [6, 11]. Scime et al. [11] state that SSC and KMC differ, with SSC an intermittent intervention, while KMC is provided continuously for a certain period of time. However, this distinction does not appear to be well supported, with some studies implementing continuous SSC [12], or intermittent KMC [13, 14].

Both SSC and KMC have been found to be associated with significant reductions in maternal symptoms of depression, in comparison to treatment-as-usual [6, 15, 16]. For example, Bigelow et al. [6] found that mothers who provided regular skin-to-skin contact for the first month of their infant’s life had lower depression scores than mothers who provided little or no skin-to-skin contact. Moreover, SSC and KMC have been found to impact positively upon parenting behaviours, including maternal sensitivity and responsiveness to infant cues [12, 16, 17], maternal confidence [18, 19], and mother-infant attachment scores [13, 20].

One way in which a mother may increase close body contact with their infant is through the use of an infant carrier, or ‘sling’. This is a structured piece of fabric that allows the parent to carry the infant against their body [21]. There are many different types of sling available (e.g. ring-sling, stretchy-wrap, structured carrier etc.) in order to suit different body shapes, postures, infant weights etc. The word ‘sling’ when discussed in this study refers to all sling types. There are clear similarities between sling use and SSC or KMC as each includes positioning the infant upright against the mother’s body, and each enables the infant to sense the mother’s breathing, temperature and heartbeat [22]. In comparison to SSC or KMC, a sling offers the opportunity for an infant to be close in this way to their parent, but with an added practicality that both infant and parent may be fully clothed for the duration of their contact [22]. However, in comparison to SSC or KMC, research into the use of slings is in its infancy [21].

One study which randomised new mothers to a sling use vs. a control intervention found that in follow up interviews, mothers reporting greater sling use were also likely to discuss themes of bonding, calming, and infant well-being [23]. A further study in which adolescent new mothers were randomized between a sling vs control intervention examined the effects of maternal-infant interaction in the still face paradigm. Findings indicated that the intervention resulted in greater social and exaggerated positive engagement from mothers, and greater social monitoring from infants. Furthermore, at 7 months, infants in the sling intervention were more likely to have secure attachments and less likely to have disorganized attachments compared to those in the control group [21].

To the best of our knowledge, no research randomly allocating participants to a sling intervention has focused on maternal mental health and psychological well-being outcomes. In addition to the maternal psychological benefits of close body contact as described above, sling use may promote mothers’ autonomy and social engagement through allowing mothers to have their hands free, travel more easily, and access a range of sling-based social networks (e.g. via social media) [24, 25]. In particular, “sling libraries” loan out slings, offering advice and information on safe and functional sling use. Furthermore, these organisations can also offer psycho-education regarding infant development and mother-infant bonding, and allow parents to connect with, and support, one another [26]. For these reasons, it may be that sling use, and in particular using slings through a sling library, can increase feelings of parental self-efficacy and improve maternal mental health [27].

Whilst the above studies on SSC and KMC examine the impact of close body contact on factors that may affect maternal mental health, studies so far have not explored whether sling use, including accessing sling-related social support or services (e.g. sling libraries), can also promote maternal psychological well-being and reduce postnatal depression symptoms. To establish such a relationship, a full-scale efficacy study, utilising a Randomised Control Trial (RCT) design is needed [28]. A full scale RCT is time-consuming and costly. It is therefore important to first evidence such a trial’s feasibility and acceptability, and to identify key study parameters, prior to undertaking the RCT. As such, this study is a feasibility study, defined by Eldridge et al. [29] as a study which asks whether, and how, something can or should be done. In this feasibility study, the research aim was to explore the feasibility and acceptability of: i) this study design; ii) a sling and sling support intervention; and iii) to provide data to estimate the parameters required to design a definitive RCT.

### Aims and objectives

The primary aim of this study is to explore participation, practicality, uptake, and acceptability, which are all aspects of RCT feasibility [30].

- Participation refers to the number eligible to participate in the study from the number screened, and the number who consent to participate, from those invited to do so. Based on studies of close body contact, KMC and other psychosocial interventions for postnatal depression, an eligibility rate of around 60% or above [31–33] and a consent rate of around 70%, or above [34–37], would be satisfactory.
- Practicality (or actual fit) refers to whether it is it possible to implement the study as it was designed within the research protocol, including whether the attrition rate is low enough to be practical. Attrition rates of higher than 15% are difficult to address using missing value methods (e.g. multiple imputation) [38]. If more than 40% of data are missing at the point of analysis, then it is unlikely that a full trial will be seen to be feasible unless significant changes are made to the study design (e.g. [39]).
- Uptake refers to the extent to which participants allocated to receive the sling intervention actually use the sling and associated support provided, and, conversely, the extent to which participants in the control group use slings independently from the study.
- Acceptability refers to whether the participants perceive participating in the study, the intervention, and the outcome measures administered, to be acceptable, operationalised here qualitatively, using the seven components of acceptability outlined by Sekhon et al. [40].

The secondary aim of this feasibility study is to gather preliminary data regarding treatment effectiveness [41]. Whilst CONSORT guidelines for feasibility studies do not recommend effectiveness testing, such preliminary analyses may generate parameters that are of use to a future RCT (e.g. effect sizes) and are acceptable as a secondary aim [29].

It is predicted that sling use with the support of sling library services will lead to lower postnatal depression scores, higher wellbeing, perceived social support and parental self-efficacy scores, and greater rates of secure mother-infant attachment (indicated by higher Quality of Attachment, Absence of Hostility, and Pleasure in Interaction scores on a mother-infant attachment measure [42]), in the intervention group, in comparison to the control group. Maternal attachment style and infant temperament are considered as possible covariates within this predicted relationship.

## Method

### Design

This is a primarily quantitative, experimental, feasibility study, which followed a predefined, pre-registered protocol, registered on OSF (https://doi.org/10.17605/OSF.IO/P23DW). Given that the primary aims of the study were feasibility related, the study was not registered on a clinical trial registry prior to completion. The study was registered on ISRCTN trial registry post-hoc, however, at the request of the publishing journal, though the pre-registered protocol address remains unchanged. There are currently no ongoing related trials, but any future related trials will be registered in advance.

Participants were randomised to one of two conditions (intervention vs. control) using a computer-generated random number sequence following a 1:1 randomisation ratio. This was conducted by the first author.

### Participants

Participants were mothers recruited while pregnant. Expectant mothers were eligible to participate if they were due to give birth within the baseline data collection period, able to travel to the sling library, and if they had not regularly used a sling previously. Mothers of twins were included in the study, but completed measures based on one child only. Mothers of infants with a serious illness or disability were excluded as they require a greater level of sling support and training than this study provided [43]. Flyers and posters were shared on social media and distributed in shops, community centres, libraries, toddler groups and cafes. Two charities; the National Childbirth Trust and Forging Families, advertised the study locally. Participants were recruited from 1^st^ April 2019. Baseline data collection concluded on on 14^th^ August 2019. Twelve-week follow up data collection concluded on 19^th^ November 2019.

As a feasibility study, sample size was selected based on whether it could adequately estimate parameters that would support the design of a future RCT, rather than a power calculation [29, 44]. Following the recommendations of the National Institute of Health Research [45], a sample size of 50–60 participants (25–30 per condition) was selected.

Ninety-one mothers expressed interest in study participation. Sixty-seven were eligible, consented, and were randomly allocated to either the intervention (*n* = 35), or control (*n* = 32) condition. Sixty-one completed baseline measures (32 intervention, 29 control) and thus were included in data analysis.

### Measures

A service user involvement group of seven mothers provided feedback regarding the acceptability and relevance of proposed outcome measures. Qualitative question phrasing and final measure selection was adjusted considering this feedback. All measures were self-reported and completed by the mothers online using Qualtrics, a web- based survey tool. Table 1 outlines the measures completed by participants throughout the study.

**Table 1:** Measures.

Primary/secondary/covariate	Measure	Description of measure and construct	Reliability and Validity	Purpose	Timepoint(s) Administered
Primary Measures	Sling and sling library use measure	This measure was developed for this study by the first author alongside service users and sling library staff. Participants indicate how often they had used a sling, pram and sling library services, over the past six weeks on a 6-point Likert scale. Participants were asked the same questions in relation to their partner (if applicable) to control for partner sling and sling library use as possible confounding variables.	N/A	To assess frequency of sling use and use of sling library services.	T1, T2, T3
	Edinburgh Postnatal Depression Scale (EPDS) [46]	10-item scale designed to screen for postnatal depression in nonclinical populations. Used widely in the National Health Service (NHS) [47]. Participants who scored above the clinical threshold for this measure were informed of this and encouraged to contact their GP or health visitor for further support.	Cronbach’s α > .80 [48, 49]	To identify symptoms of postnatal depression.	T1, T2, T3
Secondary Measures	21-item Depression Anxiety and Stress Scale (DASS-21) [50]	Participants indicated the degree to which statements applied over the past week using a 4-point Likert scale. Generated three scores: Anxiety, Depression and Stress; with higher scores indicating greater levels of each difficulty.	Has established reliability and validity with clinical and non-clinical populations Cronbach’s α > .76 [51, 52]	To supplement the EPDS as recommended [53] due to between-study variation in sensitivity and specificity of EPDS [54]	T1, T3
	Warwick Edinburgh Mental Wellbeing Scale (WEMWBS) [55]	14-item wellbeing scale. Participants rated positively worded statements on a 5-point Likert scale, with a higher total score indicating greater wellbeing.	Cronbach’s α = .91 [55]	To assess parental mental wellbeing.	T1, T3
	Parenting Sense of Competency Scale (PSCS) [56]	16-item Likert-scale questionnaire. Participants indicate agreement with statements relating to their confidence as a parent.	Cronbach’s α = .80 [57]	To assess parental self-efficacy.	T1, T3
	Maternal Postnatal Attachment Scale (MPAS) [42]	19-item Likert-scale questionnaire. Participants rated statements regarding their feelings towards their child, generating three scores; Quality of Attachment, Absence of Hostility, and Pleasure in Interaction.	Cronbach’s α = .78 [42]	To evaluate mother-infant relationship.	T1, T3
	Five-item version of the Social Provisions Scale (SPS) [58, 59]	Participants rated statements on a 7-point Likert scale. A higher total score indicates greater perceived social support.	Reported internal consistency of .65 [59].	To assess perceived social support.	T1, T3
	Measure of infant stroking by mothers [37]	Participants answered questions about how often they stroke various parts of their infant’s body. Higher scores indicate more frequent stroking.	N/A	To assess the frequency with which participants were stroking their infants.	T1, T2, T3
	Breastfeeding	Participants reported their preferred breastfeeding method.	N/A	To record feeding methods utilised by participants during the study.	T1
Covariates	12-item version of the Experiences in Close Relationships Scale (ECR-12) [60]	Participants used a 7-point Likert scale to rate their agreement with statements. Higher scores indicate higher levels of attachment anxiety or avoidance. 12 item scale.	Anxiety subscale: Cronbach’s α = 0.87 Avoidance subscale: Cronbach’s α = 0.79 [60]	To assess adult attachment style of participants to control for this as a possible covariate within data analysis.	T1
	The Infant Behaviour Questionnaire-Revised Very Short Form (IBQ-R VSF) [61]	Participants used a 7-point Likert scale to indicate the frequency with which their child displayed specific behaviours within certain situations, generating three scores: Negative Emotionality, Positive Affectivity/Surgency, and Orienting/Regulatory Capacity.	Cronbach’s α = 0.62–0.90 [62]	To evaluate infant temperament to control for this as a possible covariate within data analysis.	T3
	Infant Illness/Discomfort	Participants reported the number of days out of the past week that their infant had been unwell or experiencing physical discomfort.	N/A	To record instances of infant illness/discomfort to control for this as a possible covariate during data analysis.	T1, T2, T3

A number of factors may relate to both the independent variable (provision of sling and support vs. no sling and support provision) and the outcomes measured (e.g. PND, parenting, etc.), including: maternal attachment style, infant temperament, infant illness or discomfort and participant demographics. To potentially control for these variables, each was measured and it was planned that they would be included as covariates in effectiveness analyses should scores for each differ between conditions.

In addition to the measures listed in Table 1, participants answered a range of demographic questions, including: age, ethnicity, marital status, education, income, mental health history, infant age and infant birth order. At the end of the intervention, intervention participants also answered open-ended questions about their experience of participating in the study. These open-ended questions were designed in consultation with the local sling library, and (as described above) were reviewed by the service user involvement group.

### Procedure

Ethical approval was granted by the University of Sheffield Research Ethics Committee. Following recruitment and informed written consent procedures, participants were randomised to one of two conditions (intervention vs. control). Intervention and control participants completed the same measures at the same timepoints. Participants completed baseline measures when their child was between 0 and 6 weeks old (T1). To monitor treatment fidelity, and gather preliminary data regarding any mechanisms of change, an additional battery of measures was administered 6 weeks post-baseline (T2). T2 measures comprised the EPDS, sling/pram/sling library use, stroking, and infant illness/discomfort. At 12 weeks post-baseline (T3), participants completed the same measures as at T1, excluding demographics or the ECR-12 (as adult attachment style is considered a relatively stable trait [63]), but with the addition of qualitative questions regarding their experience of participation, and a measure of infant temperament (as infant temperament is considered a stable trait [64]. Throughout the study, participants were contacted via email or, if preferred, via SMS text. Participants were debriefed via email at the end of the study. All measures were completed online using Qualtrics. This study was not blinded, as participants were aware whether they were invited to receive their free sling and free sling session after completing T1 measures, or after completing T3 measures. The first author was the only author who had access to identifiable data during data collection (in order to invite participants to receive their free sling and free sling session following the correct timepoint for their condition). Identifiable data was deleted after study completion. It may be noted that a level of blindness was present, in that measures were self-report and were completed online using Qualtrics, which “administered” the measures without knowing which condition participants were randomised to.

#### Intervention

Upon completion of baseline measures, intervention participants were invited to attend a two-hour drop-in session at the sling library. These drop-in sessions were part of the sling library’s usual provision at the time of the study. In this usual provision, parents were welcome to stay for as long as they wished within this time-period. In usual provision, parents typically attend these sessions seeking advice and to try using a sling for the first time before buying or hiring, as well as seeking advice for slings that they are already using (e.g. through a previous purchase or hire). All contact between staff and parents takes place within one large room. As such, staff may sometimes demonstrate a sling to a group of interested parents, and parents are able to meet and chat to each other, offering opportunities for the development of social networks and social support.

To support standardisation of session content and improve replicability, a checklist was created for use by sling library staff during interactions with study participants. Following the checklist, participants were offered sling training and advice, and a sling demonstration. Participants learned how to use one of two different types of sling: a ‘Close Caboo’ or buckle carrier, dependent on the needs and preferences of the mother and their infant. Participants were then given this sling, for free hire, for the duration of the study. Participants were invited to join an online sling community for further support and were given information about safe sling use and further sling library services. Throughout the study participants were able to attend further sling library sessions and swap their sling if they had any concerns or felt that another sling may be more suited to themselves and their infant. This flexibility was designed to replicate the responsive flexibility of usual provision by the sling library, but, unlike usual provision, at no cost to the participant.

#### Control

Control participants were not offered the intervention (provision of a sling and sling library support) during the study. However, they were also not asked to refrain from sling use or from accessing the sling library during this time, as this may have represented an unethical withholding of benefits [65]. Therefore, it was possible for control participants to independently access the same sling library services as intervention participants, but with no access to free sling hire. Control participants were offered free sling hire and support following completion of T3 measures.

### Analysis

Statistical analysis of data was conducted using SPSS^®^ Statistics. Graphs were generated using Microsoft Excel. For each analysis, the significance level was set at 0.05. For Eligibility, consent, and attrition rates are described below. Frequencies and descriptive statistics for demographic measures were examined and presented for both groups. For infant illness/discomfort, maternal attachment style, and infant temperament, which were seen as potential covariates within the analysis, Mann Whitney *U* tests (infant illness/discomfort) and *t*-tests (maternal attachment style, infant temperament) were used to compare between groups.

With regards to treatment fidelity, frequency totals for sling and sling library use are presented for both groups across timepoints. These frequency total scores were calculated by assigning ordinal values to participants’ responses and totalling these values.

Between-group comparisons of sling use were conducted using Mann Whitney U tests. Participants’ partners’ sling use was also compared between groups as a possible confounding variable.

Preliminary effectiveness data was examined using Intention-To-Treat (ITT) analysis. Between-group comparisons of EPDS, possible covariate (attachment, infant temperament, infant illness), and secondary outcome measure scores, were conducted using t-tests or Mann Whitney U tests.

A 2x3 mixed ANOVA, with the between-subjects variable condition (intervention, control) and repeated variable time (T1, T2, T3) was conducted on participants’ EPDS scores. Post-hoc between-group comparison of estimated marginal means for EPDS scores was conducted. Six 2x2 mixed ANOVAs (between-subjects variable: condition (intervention, control); repeated variable: time (T1, T3)) were conducted for scores on secondary outcome measures (DASS21, WEMWBS, SPS, PSCS, MPAS, stroking).

To gather information regarding acceptability, participants’ responses to the qualitative questions asked at T3 were subject to thematic analysis procedures outlined by Braun and Clarke [66]. A deductive approach to the generation of themes was utilised [67]. Participants’ responses were coded, then clustered into a priori themes from the theoretical framework of acceptability [40]. This model conceptualises acceptability as consisting of seven component constructs: participants’ feelings towards participation (affective attitude); perceived burden; ethicality; opportunity costs; ability to implement the intervention; and intervention effectiveness.

## Results

**Fig 1:**
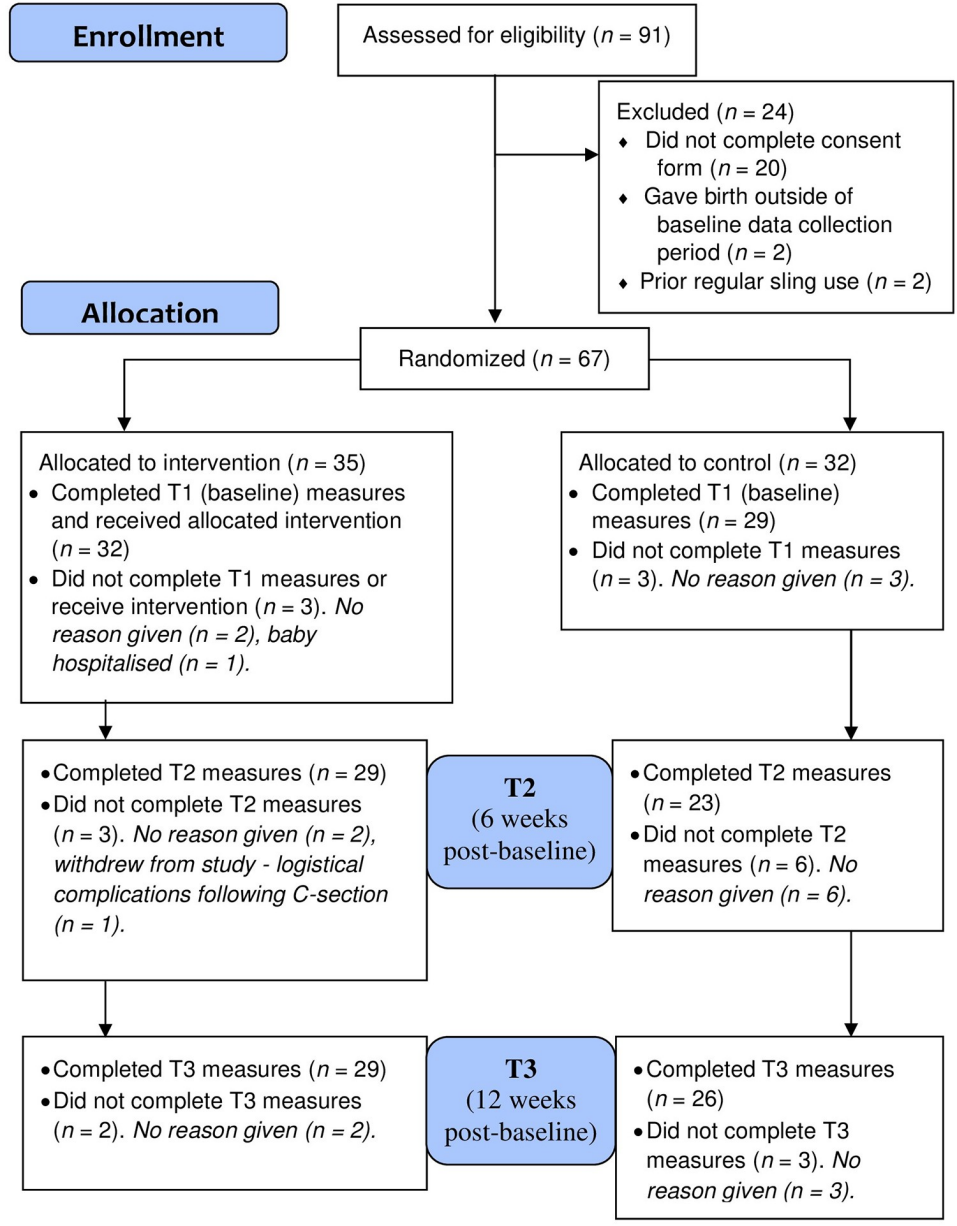
CONSORT diagram of participant flow [68].

In a feasibility study, effectiveness testing is not recommended [29]. While a preliminary effectiveness analysis did take place and is reported below, results primarily focus on findings around feasibility parameters (uptake, attrition, acceptability), and tabulated summaries of means and standard deviations (SDs). Fig 1 shows the participant flow diagram.

As seen in Fig 1, of the 91 mothers who expressed an interest, 87 (96%) were eligible to participate. Of the 87 mothers eligible to participate, 67 (77%) consented. Mothers who did not consent to participate automatically did not consent to further contact. Therefore, information was not gathered regarding reasons for non-consent. Of the 67 mothers who consented to participate, 61 (91%) completed T1 measures. Only those who completed T1 measures were asked to complete T2 and T3 measures. Of the participants who completed T1 measures, 52 (85%) completed measures at T2, and 55 (90%) at T3, with 50 completing measures at all three time-points, giving an overall attrition rate of 18%. Most of the participants who discontinued gave no reason for their discontinuation. Of the 61 participants who completed T1 and thus were included in data analysis, 12 (20%) had data missing for at least one variable over at least one timepoint. Out of a total of 4,148 possible values in the dataset, 127 (3%) were missing. For all participants, if the participant completed one quantitative measure at a time-point, then they completed all of the quantitative measures.

### Sample characteristics

Table 2 shows maternal and infant characteristics and demographics by condition. Infant illness/discomfort, maternal attachment style and infant temperament were identified as possible covariates within analysis. As such statistical comparisons were conducted to identify any significant between-group differences for these variables. Descriptive statistics and comparisons for each of these three variables are presented in Table 3. Due to the ordinal nature of the data, Mann-Whitney *U* tests were used to conduct between-group comparisons for infant illness/discomfort, maternal attachment style, and infant temperament scores. There were no significant differences between the two groups for any of these three variables.

**Table 2:** Intervention and control group characteristics.

Characteristics (when measured, if only once)	Categories	Intervention Group (*n* = 32)	Control Group (*n* = 29)
		n (%)	n (%)
Infant’s age in weeks (T1)	n/a	1.4 (1.1)	1.2 (1.1)
Mother’s age (T1)	Under 18	0	0
	18–25	2 (6.3)	0
	26–35	21 (65.6)	22 (75.9)
	36–45	8 (25.0)	7 (24.1)
	46–55	1 (3.1)	0
	Over 55	0	0
# Child (T1)	Firstborn (1)	25 (78.1)	25 (86.1)
	Second born (2)	4 (12.5)	2 (6.9)
	Third born (3)	2 (6.3)	2 (6.9)
	Fourth born (4)	1 (3.1)	0
	Fifth born + (5)	0	0
Ethnicity^a^ (T1)	White British	25 (78.1)	27 (93.1)
	Asian/Asian British	2 (6.3)	0
	Mixed Asian/White British	1 (3.1)	0
	White European	2 (6.3)	1 (3.4)
	White—Other	1 (3.1)	0
	South American/	1 (3.1)	0
	Latin American	0	1 (3.4)
Marital status (T1)	Single	2 (6.3)	0
	Married	22 (68.8)	16 (55.2)
	Co-habiting	7 (21.9)	11 (37.9)
	In a relationship, not co-habiting	0	2 (6.9)
	Separated/divorced	1 (3.1)	0
	Widowed	0	0
	Single	0	0
Employment (T1)	Employed full-time	22 (68.8)	20 (69.0)
	Employed part-time	6 (18.8)	6 (20.7)
	Unemployed	2 (6.3)	1 (3.4)
	Student	0	1 (3.4)
	Other	2 (6.3)	1 (3.4)
Partner’s employment (T1)	Employed full-time	27 (84.4)	26 (89.7)
	Employed part-time	1 (3.1)	1 (3.4)
	Unemployed	2 (6.3)	0
	Student	0	0
	Other	1 (3.1)	2 (6.9)
	N/A	1 (3.1)	0
Education (T1)	High school	0	2 (6.9)
	Apprenticeship	0	0
	College Qualification	5 (15.6)	5 (17.2)
	University—UG degree	13 (40.6)	8 (27.6)
	University—PG degree	12 (37.5)	13 (44.8)
	Professional /other vocational qualification	2 (6.3)	1 (3.4)
Income (T1)	Less than £10,000	0	0
	£10,000-£19,999	4 (12.5)	1 (3.4)
	£20,000- £29,999	1 (3.1)	5 (17.2)
	£30,000-£39,999	2 (6.3)	2 (6.9)
	£40,000-£49,999	5 (15.6)	5 (17.2)
	£50,000-£59,999	6 (18.8)	5 (17.2)
	£60,000 or over	14 (43.8)	11 (37.9)
Postcode affluence (T1)	Affluent	11 (34.4)	10 (34.5)
	Not affluent	21 (65.6)	19 (65.5)
Infant feeding method (T1)	Formula	1 (3.1)	1 (3.4)
	Breastfeeding	25 (78.1)	18 (62.1)
	Both formula and breastfeeding	6 (18.8)	10 (34.5)
Current maternal mental health (T1)	Good	18 (56.3)	22 (75.9)
	Somewhat good	9 (28.1)	5 (17.2)
	Average	3 (9.4)	2 (6.9)
	Somewhat poor	2 (6.3)	0
	Poor	0	0
Previous mental health diagnosis (T1)	Yes, prior to pregnancy	14 (43.8)	12 (41.4)
	Yes, during pregnancy	1 (3.1)	0
	No	17 (53.1)	17 (58.6)
Accessing mental health support (T1)	Yes	7 (21.9)	2 (6.9)
	No	25 (78.1)	27 (93.1)
Family history of mental ill-health (T1)	Yes	15 (46.9)	12 (41.4)
	No	14 (43.8)	16 (55.2)
	I don’t know	3 (9.4)	1 (3.4)

^a^ *Note*: Only ethnicities that were selected by participants appear in this table.

**Table 3:** Comparison of intervention and control group characteristics for infant illness, maternal attachment style and infant temperament.

Characteristics (when measured, if only once)	Timepoint/Subscales	Intervention Group (*n* = 32)	Control Group (*n* = 29)	Test of difference or association	*P*
		Mean (SD)	Mean (SD)		
				*U*	
Infant illness/discomfort	T1	3.2 (4.7)	3.6 (3.4)	363.50	.141
	T2	3.9 (3.5)	5.0 (4.4)	406.50	.401
	T3	3.6 (3.6)	3.3 (3.3)	458.00	.930
Maternal attachment style (T1)	Anxiety	3.1 (0.9)	3.1 (1.0)	459.00	.942
	Avoidance	1.9 (0.9)	1.5 (0.6)	588.00	.071
Infant temperament (T3)	Positive Affectivity/Surgency	3.7 (1.0)	4.1 (0.8)	365.00	.152
	Negative Emotionality	3.4 (0.9)	3.5 (0.9)	393.00	.305
	Orienting/Regulatory Capacity	5.0 (0.7)	4.7 (0.7)	564.00	.148

Eleven mothers did not complete outcome measures at all three timepoints. Completer vs non-completer comparison analyses found no differences between completers and non-completers on any demographic or baseline measures and no differences on adult attachment style. There were differences in infant temperament between completers and non-completers (S1 Table), with non-completers rating their infants higher in Positive Affectivity/Surgency and Negative Emotionality, and lower in Orienting/Regulatory Capacity than completers.

**Fig 2:**
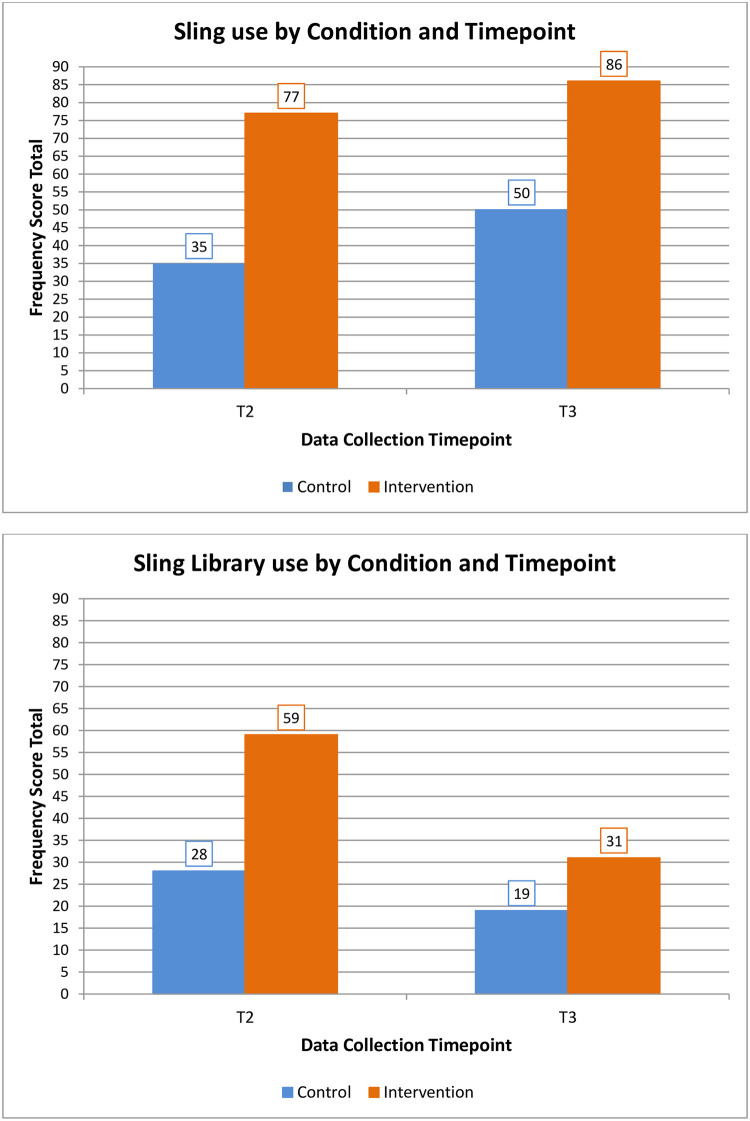
Total sling and sling library use frequency scores at each timepoint by condition.

All intervention participants had used a sling in the past six weeks at both T2 and T3. At T2, 5/23 control participants (22%) had not used a sling in the past six weeks, decreasing to 2/26 (8%) at T3. Fig 2 displays sling and sling library use total frequency scores at T2 and T3 for each condition.

Mann-Whitney U tests found a significant between-group difference in sling use frequency both at T2 (*U* = 230.50, *p* < .001) and at T3 (*U* = 211.00, *p* < .001), with median scores higher for the intervention (Mdn = 3) than the control group (Mdn = 2) at both timepoints.

Mann Whitney tests found a significant between-group difference in participants’ sling library use at T2 (*U* = 313.50, *p* = .023), but not T3 (*U* = 398.00, *p* = .292), with median frequency scores 3 (intervention) and 0 (control), and 0.5 (intervention) and 0 (control) at T2 and T3, respectively. No significant between-group differences in partner sling or sling library use frequencies were found at either T2 (sling: *U* = 381.00, *p* = .208; sling library: *U* = 388.50, *p* = .174) or T3 (sling: *U* = 368.00, *p* = .141; sling library: *U* = 419.00, *p* = .292), see S1 Fig.

### Effectiveness

As a small feasibility study, this study was not designed to detect between-group differences, and effect size calculations were unlikely to be reliable [29]. Still, ANOVAs were conducted in order to provide an initial estimate of the effect of group allocation. Assumptions of normal distribution of the dependent variable and homogeneity of variance, and the presence of outliers, were checked prior to each analysis. All variables, including the EPDS, were found to have non-normal distributions at one or more timepoints, with the exception of the PSCS. All variables met the homogeneity of variance assumption. There were outliers present at one or more timepoints for the majority of variables, with the exception of the WEMWBS, PSCS, MPAS Quality of Attachment subscale, and stroking.

ANOVAs may be robust to non-normality and the presence of outliers, particularly if the homogeneity of variance assumption is met [69]. Thus, ANOVAs were conducted, accepting the possibility of an increased likelihood of Type 1 error (false positive). Groups did not differ significantly in infant illness/discomfort and adult attachment style (measured at baseline (T1)), infant temperament (measured at T3), or partner sling and sling library use at T2 and T3. It was therefore not necessary to include any covariates within the analyses [70].

#### Postnatal depression symptoms

Across the three data collection timepoints, a total of nine participants met the clinical threshold for PND on the EPDS. For those scoring at, or higher than, this threshold, an email was sent to the participant, encouraging them to speak to their GP or health visitor. To assess change in the primary outcome measure of PND, a 2 (Group: intervention vs. control) x3 (Time: T1,T2, T3) mixed ANOVA was conducted examining EPDS scores. There were no significant main effect for either time (*F*(1.68, 98.98) = 2.33, *p* = .111) or condition (*F*(1, 59) = 0.36, *p* = .553), and no significant time*condition interaction (*F*(1.68, 98.98) = 1.85, *p* = .169). Follow-up comparisons of estimated marginal means (EMMs) were conducted for EPDS scores at each timepoint and are presented in Table 4. No significant between-group differences were found at any timepoint. Though non-significant, a small effect size was shown (*d* = .23), with mean EPDS scores lower for the intervention group at T3 than for the control group.

**Table 4:** Group comparison of EPDS estimated marginal means.

Variable	Timepoint	Intervention Group (*n* = 32)		Control Group (*n* = 29)		Mean Difference	*p*
		Mean	SE	Mean	SE		
Postnatal	T1	8.0	0.8	7.2	0.8	0.73	.514
Depression	T2	7.1	0.8	5.7	0.9	1.45	.240
(EPDS)	T3	6.4	0.5	7.1	0.6	0.70	.381

Table 5 shows mean scores for secondary measures across the timepoints and the results of post hoc between-group comparisons of these scores. Mann Whitney U tests found no significant between-group differences for any secondary measure scores at any timepoint.

**Table 5:** Group comparisons on secondary outcome scores.

Variable	Subscale	Timepoint	Intervention Group (n = 32)	Control Group (n = 29)	*U*	*p*
			Mean (SD)	Mean (SD)		
Mental health (DASS21)	Depression	T1	5.0 (4.7)	4.5 (3.9)	449.50	.832
		T3	4.5 (5.7)	5.2 (6.0)	437.50	.692
	Anxiety	T1	5.8 (6.8)	4.3 (3.6)	448.00	.814
		T3	3.7 (4.6)	2.9 (3.0)	462.50	.982
	Stress	T1	13.3 (9.1)	11.4 (6.7)	426.50	.587
		T3	11.4 (8.8)	13.5 (8.9)	389.50	.280
Wellbeing (WEMWBS)	Total score	T1	49.6 (8.0)	50.6 (8.5)	397.00	.333
		T3	53.0 (8.9)	50.3 (9.9)	535.50	.301
Parental self-efficacy (PSCS)		T1	29.9 (4.5)	31.2 (3.1)	393.00	.302
		T3	78.5 (8.9)	73.9 (9.7)	588.50	.072
Mother-child relationship (MPAS)	Quality of Attachment	T1	39.3 (4.5)	39.7 (6.0)	394.00	.309
		T3	40.0 (4.2)	40.4 (4.3)	417.50	.499
	Absence of Hostility	T1	20.0 (2.5)	20.3 (3.6)	411.00	.443
		T3	18.4 (3.1)	18.4 (3.7)	460.50	.960
	Pleasure in Interaction	T1	22.0 (2.7)	21.4 (4.2)	462.50	.982
		T3	22.6 (2.4)	20.9 (3.9)	345.50	.083
Perceived social support (SPS)		T1	71.1 (10.6)	72.2 (1.9)	393.00	.302
		T3	30.2 (3.3)	30.3 (4.1)	423.00	.551
Maternal stroking of infant		T1	12.5 (2.9)	12.7 (2.4)	440.50	.731
		T2	12.7 (2.6)	12.4 (2.7)	435.00	.672
		T3	13.6 (2.5)	13.0 (2.5)	392.50	.296

To assess the secondary outcome variables, six 2 (Time: T1 vs T3) x 2 (Group: intervention vs control) mixed ANOVAs were conducted. Anxiety and absence of hostility were found to significantly decrease (DASS Anxiety subscale: *F*(1, 59) = 5.13, *p =* .027; MPAS Absence of Hostility subscale: *F*(1, 59) = 21.41, *p* < .01), whilst maternal self-efficacy and stroking of infants were found to significantly increase (PSCS: *F*(1, 59) = 11.57, *p* < .01; Stroking: *F*(1, 59) = 3.47, *p =* .034), over time for both conditions.

**Fig 3:**
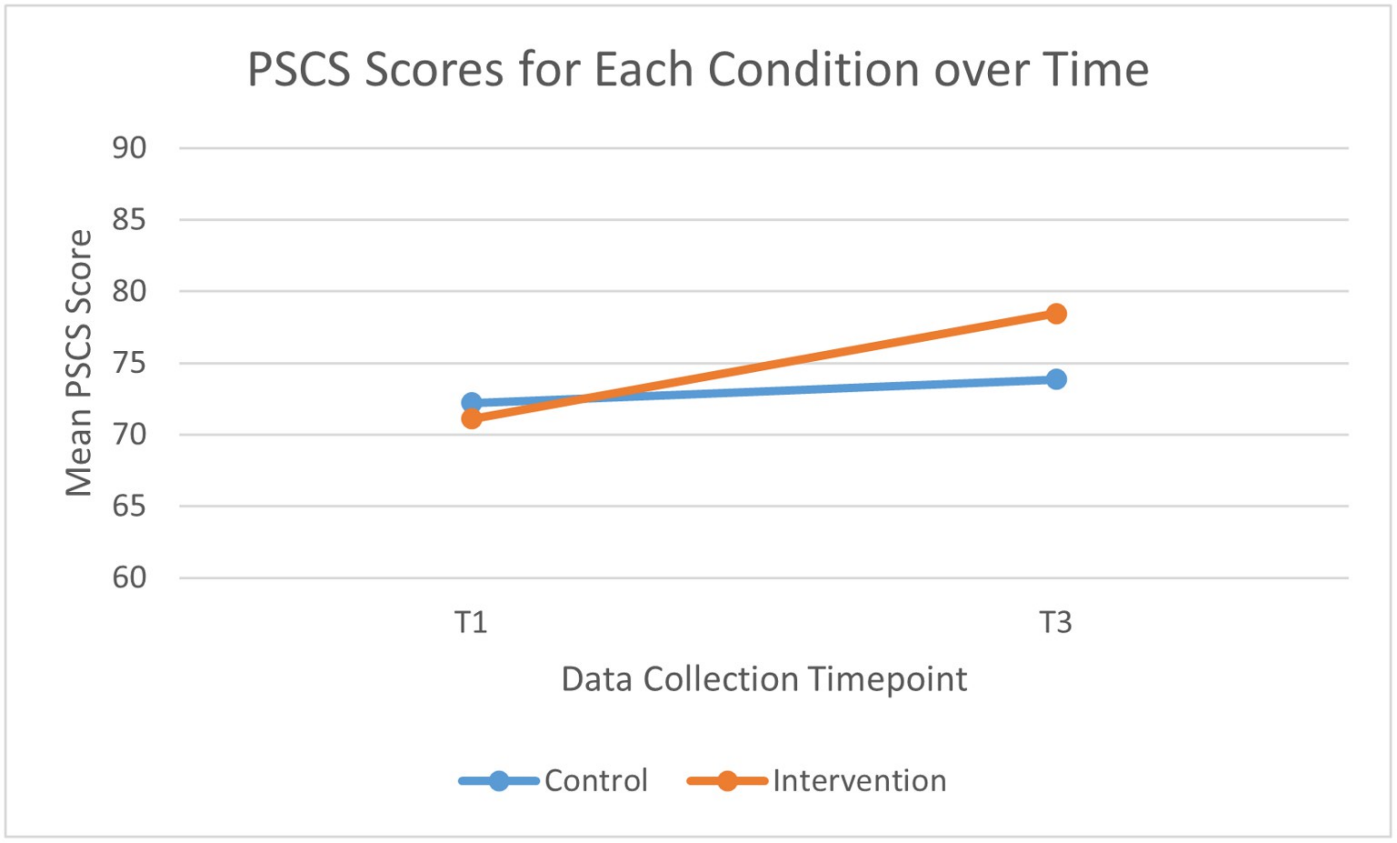
Mean PSCS scores by condition at T1 and T3.

For parental self-efficacy scores, a significant time*condition interaction was found (*F*(1, 59) = 4.64, *p =* .035), with a medium effect size shown (η_p_^2^ = .07). Examination of means indicated that PSCS scores for the intervention group showed a significantly greater increase than control PSCS scores (Fig 3).

### Acceptability

Twenty-nine intervention and eight control participants completed qualitative questions at T3. Responses were coded, with codes clustered into a priori themes from the theoretical framework of acceptability [40]. Illustrative quotations for each theme can be found in Table 6. Sekhon [40] defines the first component, Burden, as the perceived effort of participation. Eight participants described participation in the study as “not a problem” and the outcome measures as “ok” to complete. In contrast three participants described the outcome measures as “long”, repetitive, or onerous to complete. Thirteen participants described using a sling as “easy”, whilst another eight reported that using a sling was difficult at first, but became easier over time.

**Table 6:** Illustrative quotations mapped onto Sekhon et al.’s (2017) model of acceptability themes.

Theme	Example
Burden	“The surveys were a bit long but it was worth it for the experience of going to the sling library and getting a sling for free.” “The surveys are easy to complete.” “It [the sling] was so easy to use so we started using it every day immediately.”
Affective Attitude	“Really interesting, really enjoyed the use of the sling, especially when trying to do something or soothe baby to sleep.” “Enjoyable, interesting answering the questionnaires.”
Ethicality	“I enjoyed the thought that our responses might assist with research in some way “Being able to contribute to something meaningful, and taking time to check in with my mental health.” “I have felt privileged to be part of this study. It has made me consider my own emotions in relation to motherhood…I have enjoyed the opportunity to think about my own mood and emotions and the bonding process between me and my son.” “Mildly distressing—it made me understand that I’ve been having difficulty in moderating my mood and has somewhat made me question whether my own mental health is having a detrimental impact on my baby”
Intervention Coherence	“Some questions are worded a little confusing.” “I appreciated having the expert instruction; I wouldn’t have felt confident wearing such a small baby otherwise.” “Learning how to carry our baby safely. I think for us the advice was key to us having a carrier that worked for what we needed. It felt like we were really listened to and we appreciated all the advice.”
Opportunity Costs	“Great to get free sling hire for 3 months…” “The sling library made all the difference this time and this study gave me access to their services when I might not usually have had the confidence to go there to seek advice. I am so so glad I took part.” “Having baby close but being able to do basic things such as walking the dog or hanging out washing.” “I can get so much done while baby wearing, it’s so much easier to travel outside of the house with baby wearing rather than the pram and best of all: my baby loves being in it!” “The sling gave me freedom that a pram wouldn’t have done.”
Perceived Effectiveness	“A greater choice of slings may have been better…” “…my baby loves it! It’s her favourite place to be, she is instantly soothed and often naps while in the sling.” “There is nothing that I dislike about the sling. My son loves to be close to me and the sling enables us to be close even when I need my hands. There have been times where he has been upset and I have put him in the sling and it has soothed him. It has enabled us to go for walks together in beautiful countryside.” “I love the sense of closeness to my baby. After a somewhat turbulent start in hospital with lots of medical intervention, I feel I am able to bond more with my child.” “We found the sling library a friendly and welcoming place. The lady who saw us helped us work out which sling or carrier was best for us at that point and was very patient teaching us how to use it correctly.” “Being able to drop in (rather than make an appointment) to a friendly patient environment where I can also breast feed comfortably and meet other parents.” “If I am honest, it felt a bit rushed.” “…a one to one would have been better. There were lots of people waiting for support during that clinic and I felt a bit “watched” by the others and felt like I needed to understand how to use the sling quickly because there were others waiting.”
Self-efficacy	“I feel confident using a sling thanks to their [the sling library’s] help.” “[It] took time to build up confidence with sling and to use it without having buggy on hand.”

The second component, Affective Attitude, refers to the feelings expressed by participants towards the study. Twenty-four participants expressed positive feelings (“interesting”, “enjoyable”) towards study participation, or using a sling or the sling library.

The third component is Ethicality, which is defined as the extent to which the study fits with participants’ values. Twelve participants reported that it felt positive to contribute to research, and 14 participants reported that completing the outcome measures offered them the opportunity to reflect on their mental health and experiences of motherhood. Whilst the majority of participants spoke about this as a positive, two mothers found it distressing.

The fourth component is Intervention Coherence, which refers to participants’ understanding of the intervention. Most participants did not comment on their understanding of the intervention or of the study as a whole. However, participants described the sling library sessions as informative or helpful when learning to use their sling (n = 19). One participant described outcome measure questions as “confusing”.

The fifth component is Opportunity Costs, which is the extent to which participants gave up benefits or values to engage in the intervention (study). Participants appeared to report gains rather than losses, such as gaining free sling hire (n = 7), being made aware of the sling library (n = 6), and the practical benefits and increased freedom of using a sling (n = 13). Some participants reported wanting more sling options (n = 8) and one-to-one sling library consultations or further sessions (n = 9), to be included in the intervention.

The sixth component is Perceived Effectiveness, defined as the extent to which participants view the intervention as likely to achieve its purpose. Participants described the sling and support intervention as useful or helpful (n = 11) and sling library staff as friendly and knowledgeable (n = 16). Participants listed a number of positive effects of the intervention, including: their baby enjoying being in the sling and being easier to soothe or settle when in the sling (n = 14), feeling closer, or bonding, with their baby, feeling more confident as a parent (n = 20), and the opportunity to meet other parents (n = 10). Twenty-four participants reported positive and effective experiences of the sling library specifically. Eight described their sling library session as rushed or overwhelming.

The final component is Self-Efficacy, which refers to participants’ confidence in their ability to implement the intervention. Attending the sling library session and practice were described by participants as helpful for building confidence in using a sling (n = 8).

## Discussion

This study explored whether it is feasible to conduct a randomised trial examining whether sling use and support impacts upon maternal mental health and wellbeing (including variables such as parental self-efficacy, perceived social support and rates of secure mother-infant attachment). This study aimed to support the design of a future RCT through the assessment of feasibility indicators (recruitment, attrition), acceptability of the intervention, and preliminary effectiveness data. Both eligibility and consent rates (96% and 77%, respectively) were found to meet this study’s feasibility objectives, indicating that mothers of newborns are able and willing to participate in a sling and sling support intervention trial. While this study’s consent rate is similar to rates seen in feasibility studies of other close body contact interventions, such Kangaroo Mother Care [35], or maternal stroking [37], the eligibility rate is higher than rates seen in studies of other psychosocial interventions [31, 33]. In this study, eligibility criteria were included on recruitment materials, perhaps increasing the likelihood that mothers contacting the research team would be eligible to participate.

The majority of participants completed measures at all three timepoints across 12 weeks, however, attrition was higher than 15%. This rate is comparable to that seen in Neu and Robinson’s study [71], which also took place within a community setting, and examined the impact of skin-to-skin contact upon mother-infant interactions across six months. Hospital-based studies of close body contact interventions appear to show lower rates of attrition [18, 20, 72]. It is not possible to suggest effective methods for promoting participant engagement and retention in future sling studies on the basis of this study. This is because, for the majority of mothers who did not consent to participate or discontinued participation, information was not gathered regarding reasons for non-consent or discontinuation. In total, less than 20% of data was missing at the point of analysis, indicating that a full-scale RCT may be feasible [39].

By their nature, effectiveness testing is not the primary aim of a feasibility studies and is not recommended given small sample sizes; however preliminary effectiveness analyses are acceptable as a secondary aim [29]. As such, hypotheses were given for this study regarding the possible impact of the sling and support intervention. It was predicted that that sling use with the support of sling library services would lead to lower postnatal depression scores, higher wellbeing, perceived social support and parental self-efficacy scores, and greater rates of secure mother-infant attachment in the intervention group, in comparison to the control group.

Preliminary effectiveness analyses found a significant effect of the sling and support intervention upon parental self-efficacy scores, such that participants in the intervention group showed significant improvement in these scores in comparison to control group participants. No other statistical analyses found a significant effect of intervention group. However, this small feasibility study was not designed to detect between-group differences, and effectiveness analyses are unlikely to be reliable [73]. Aside from the positive effect of the intervention on parental self-efficacy scores, these quantitative findings do not reflect qualitative feedback from participants, who spoke about enjoying using the sling and sling library services, and attributed improvements in their relationship with their baby, and increased autonomy and social engagement, to the sling and support intervention.

This contrast between the quantitative and qualitative results may simply reflect that the study was not powered to detect the effects of the sling and support intervention upon outcome measure scores [73]. It should be noted that the effects seen, though small (*d* = .23) and non-significant, were in the expected direction, with intervention participants showing lower mean EPDS scores at T3 than control participants. These data can inform the sample size for a later study. This study design, conducted with a larger sample, may be more able to capture the outcomes reported within the qualitative feedback, and to show a statistically significant relationship between a sling and support intervention, and maternal psychological outcomes.

Moreover, generally participants’ scores on the EPDS were low, with average scores for T1, T2 and T3 all below the clinical cut-off for this measure. Given the sample for this study was a non-clinical population of mothers who elected to participate, it may be that the sampling method employed produced a floor effect, whereby it was not possible to evidence significant change in EPDS scores over time or according to condition, as they were already low to begin with. The limitations of the volunteer sampling method employed are discussed further in the “Limitations and Recommendations” section below, and recommendations are made for a future, larger-scale RCT to employ alternative methods.

There were no difficulties found regarding engaging mothers in the intervention group with the sling and support intervention. This is in contrast to Bigelow and Power’s study of the impact of SSC upon mother-infant interaction, in which 33% of participants in a skin-to-skin contact intervention condition were excluded from the study due to poor treatment adherence [74]. Most control participants also engaged in sling use and accessed sling library services, but to a lesser extent than mothers in the intervention group. These findings indicate that mothers may be motivated to use slings without encouragement or support, but implementing a sling and support resource can be helpful in supporting mothers to use slings.

The majority of participants appear to have found the sling and support intervention, and the study as whole, acceptable. Participants appeared to value contributing to research in this area, and also the apparent benefits of the sling for their baby (e.g. being easier to soothe, sleeping more) or for themselves (increased autonomy, feeling closer to their baby and less anxious as a parent, meeting other parents). A number of participants wished for greater one-to-one sling library input as part of the intervention, with some describing the drop-in session as busy or rushed.

In terms of the outcome measures, a number of participants appeared to view completing the measures as a valuable opportunity to reflect on their mental health and their relationship with their child. A small minority found completing the measures distressing, confusing or onerous. It seems likely that the process of gaining feedback regarding possible outcome measure batteries from a service user involvement group contributed to the acceptability of these measures within this study [75].

Overall the majority of participants reported positive feelings regarding their participation in the study, using a sling, or accessing sling library services, with very few mothers describing participation as onerous or distressing. However it must be noted that this feedback is gained exclusively from participants who completed the study with minimal information collected from those that discontinued participation. Nevertheless, this study indicates that sling use with support may be viewed by mothers as an acceptable psychosocial intervention for mood or wellbeing following birth, should future studies establish a significant positive impact of sling use and support upon maternal mental health.

### Limitations and recommendations

Due to the study design, it was not possible to provide a population level screening rates (i.e, the proportion of the target population appropriate to screen for eligibility), or to comment on reasons for discontinuation. Without this information it is difficult to understand barriers to engagement within this research area. Moreover, qualitative feedback was only provided by those who completed the study. It is likely that this biases findings with regards to the acceptability of the study.

A volunteer sampling method was utilised. Social media and word-of- mouth were found to be effective recruitment strategies. However, the use of such methods increase the risk of selection bias and of contamination between conditions, with intervention participants perhaps discussing slings and the sling library with control participants. Indeed, the majority of participants were from a similar demographic background (White British, educated, high annual income) and some mothers knew each other through community or parenting groups. This also increased the risk of social desirability effects upon study results. The presence of selection bias and contamination brings into question the validity and generalisability of the results of this study. To reduce the risk of selection bias, it would have been better to utilise a random sampling method across several, demographically diverse, locations and settings. Moreover, in a future RCT, cluster randomisation methods may be used to reduce the risk of contamination [76].

The use of a Likert scale-based idiographic measure to record frequency of participant sling, pram and sling library use, limits the extent to which participants’ use of these resources was accurately captured. For example, within the sling use Likert scales, response options went from “a few times” to “once a day” with regards to frequency of use over the past six weeks. Participants using a sling about once a week would have to choose between these two options, neither of which truly reflects this frequency. Asking participants to keep a diary of time spent using their sling, and accessing sling library services (including online services, e.g. their social media page), may have better captured participants’ sling and sling library use, as participants would not have been required to choose between set response options which may not be reflective of their actual frequency of sling or support use.

The intervention received within this study was matched to the service that mothers typically receive when accessing slings via a sling library, promoting the ecological validity of this study’s results. However as such, no steps were taken to ensure that the sling and support intervention was standardised across participants other than providing the sling library with a session checklist to use. Fidelity to the intervention model outlined by this checklist was not monitored (e.g. by recording and rating library sessions for fidelity to the checklist). This makes it more difficult to attribute any effects seen to the intervention, and may have increased the likelihood of a Type 1 error. Future studies may wish to consider developing a manualised sling intervention, within a one-to-one or group setting (rather than a drop-in session as seen in this study), and monitoring fidelity to the treatment model, in order to support standardisation of the intervention across participants. Such an approach may minimise the risk of Type 1 error, but would have more limited ecological validity than this study.

Lastly, this study examined only maternal outcomes. However, fathers are also primary caregivers, and they use slings [25]. While it is noteworthy that in this study, participants’ partners did not differ in their sling use at T2 or T3, future research should examine the uptake and impact of a sling-based interventions on both parents to better understand any family-level effects.

As this is a feasibility study, in which the main questions are whether and how a study should be done [44], without even the level of detail of a pilot study in which specific elements or a smaller-scale version of the exact RCT design is conducted [77], it seems more appropriate to focus recommendations or implications towards the next steps for research rather than attempting to offer direct implications for clinical practice at this very early stage. A number of recommendations are proposed for future research. Using information gathered from this study to calculate parameters, a larger, appropriately powered, study should be conducted in order to effectively examine the impact of a sling and support intervention upon maternal psychological outcomes within the community. Future studies may wish to utilise a manualised sling intervention, and to take steps to monitor adherence to the intervention model, in order to support standardisation across participants. The present study was limited in the extent to which it recorded reasons for participant non-consent or discontinuation. Future studies in this area should work to gather such information so that barriers to engagement may be better understood. Implementation of a “sling and support” model, similar to the intervention seen in this study, may be helpful in promoting sling use. In a future RCT, a model such as this may support participant engagement in a sling intervention condition. Future research should also consider the method by which sling use is reported. It may be better to ask mothers to record hours using a sling, rather than using a frequency-based Likert-scale. Future studies may find it helpful to test proposed batteries of outcome measures with a focus group of mothers, in order to increase the likelihood that the measures used within the study will be acceptable, appropriate and relevant. Future research should examine the impact of sling use and support upon paternal, as well as maternal, psychological outcomes, as fathers may also be a primary caregiver.

## Conclusions

Overall, this study found it feasible to recruit mothers of young infants, to implement a sling use and support intervention within the community, and to collect relevant outcome data. There were a number of limitations to this study, particularly with regards to the sampling methods employed and difficulties around standardisation of the intervention across participants. Nevertheless, it is hoped that the information gathered in this study supports the design of a future RCT; particularly as qualitative feedback from participants indicates that sling use and support may be an acceptable psychosocial intervention for mothers that could support maternal mental health.

## Supporting information

S1 Table Comparison of completer and non-completer characteristics. (DOCX)

S1 Fig Partner sling use by timepoint and condition. (TIF)

S2 Fig Partner sling library use by timepoint and condition. (TIF)

S1 Checklist CONSORT 2010 checklist. (DOC)

S1 Protocol Research protocol version 3. (PDF)
