# Supplementary material for: Evaluating the impact of sling provision and training upon maternal mental health, wellbeing and parenting: A randomised feasibility trial
Source: PLoS One. 2023 Nov 10;18(11):e0293501. doi: 10.1371/journal.pone.0293501 (PMC10637655; doi:10.1371/journal.pone.0293501)
Supplement: S1 Table — (DOCX) [file pone.0293501.s001.docx]

**Table S1**

**Comparison of completer and non-completer characteristics**

|  |  | Participants with No Missing Data  (n = 50) | Participants with Data Missing  (n = 11) |  |  |
| --- | --- | --- | --- | --- | --- |
| Characteristics (when measured, if only once) | Categories/  subscales | Mean (SD) or n (%) | Mean (SD) or n (%) | Test of difference or association | *p* |
|  |  |  |  | *U* |  |
| Infant’s age in weeks (T1) | n/a | 1.4 (1.1) | 1.1 (1.0) | 227.50 | .310 |
| Infant Illness/Discomfort (T1) | n/a | 3.3 (4.1) | 3.4 (4.1) | 268.50 | .902 |
| Infant temperament (T3) | Positive Affectivity/Surgency  Negative Emotionality | 3.7 (1.0)  3.3 (1.0) | 4.7 (0.2)  3.9 (0.4) | 451.00  390.50 | <.001  .030 |
|  |  |  |  | *t* |  |
| Infant temperament (T3) | Orienting/Regulatory Capacity | 5.0 (0.6) | 4.4 (0.7) | 2.69 | .009 |
| Maternal attachment style (T1) | Anxiety  Avoidance | 3.1 (0.9)  1.7 (0.8) | 3.1 (0.9)  1.8 (0.8) | 0.09  -0.07 | .932  .948 |
|  |  |  |  | *χ*^2^ |  |
|  |  |  |  |  |  |
| Mother’s age (T1) | Under 18  18-25  26-35  36-45  46-55  Over 55 | 0  2 (4)  35 (70)  12 (24)  1 (2)  0 | 0  0  8 (72.7)  3 (27.3)  0  0 | 0.71 | .871 |
| # Child (T1) | Firstborn (1)  Second born (2)  Third born (3)  Fourth born (4)  Fifth born + (5) | 42 (84)  4 (8)  4 (8)  0  0 | 8 (72.7)  2 (18.2)  1 (9.1)  0  0 |  |  |
| Ethnicity^a^ (T1) | White British  Asian/Asian British  Mixed Asian/White British  White European  White – Other  South American  Latin American | 41 (82)  2 (4)  1 (2)  3 (6)  1 (2)  1 (2)  1 (2) | 11 (100)  0  0  0  0  0  0 | 2.32 | .985 |
| Marital status (T1) | Single  Married  Co-habiting  In a relationship, not co-habiting  Separated/divorced  Widowed | 2 (4)  31 (62)  15 (30)  1 (2)  1 (2)  0 | 0  7 (63.6)  3 (27.3)  1 (9.1)  0  0 | 2.07 | .723 |
| Employment (T1) | Employed full-time  Employed part-time  Unemployed  Student  Other | 34 (68)  11 (22)  2 (4)  1 (2)  2 (4) | 8 (72.7)  1 (9.1)  1 (9.1)  0  1 (9.1) | 1.97 | .742 |
| Partner’s employment (T1) | Employed full-time  Employed part-time  Unemployed  Student  Other  N/A | 44 (88)  1 (2)  1 (2)  0  3 (6)  1 (2) | 9 (81.8)  1 (9.1)  1 (9.1)  0  0  0 | 3.69 | .450 |
| Education (T1) | High school  Apprenticeship  College Qualification  University - UG degree  University – PG degree  Professional /other vocational qualification | 1 (2)  0  8 (16)  19 (38)  20 (40)  2 (4) | 1 (9.1)  0  2 (18.2)  2 (18.2)  5 (45.5)  1 (9.1) | 2.98 | .561 |
| Income (T1) | Less than £10,000  £10,000-£19,999  £20,000- £29,999  £30,000-£39,999  £40,000-£49,999  £50,000-£59,999  £60,000 or over | 0  3 (6)  5 (10)  3 (6)  9 (18)  10 (20)  20 (40) | 0  2 (18.9)  1 (9.1)  1 (9. 1)  1 (9. 1)  1 (9. 1)  5 (45.5) | 2.87 | .720 |
| Postcode affluence (T1) | Affluent  Not affluent | 19 (38)  31 (62) | 2 (18.2)  9 (81.8) | 1.57 | .210 |
| Infant feeding method (T1) | Formula  Breastfeeding  Both formula and breastfeeding | 2 (4)  35 (70)  13 (26) | 0  8 (72.72)  3 (27.27) | 0.46 | .796 |
| Current maternal mental health (T1) | Good  Somewhat good  Average  Somewhat poor  Poor | 34 (68)  10 (20)  4 (8)  2 (4)  0 | 6 (54.6)  4 (36.4)  1 (9.1)  0  0 | 1.75 | .625 |
| Previous mental health diagnosis (T1) | Yes, prior to pregnancy  Yes, during pregnancy  No | 23 (46)  0  27 (54) | 3 (27.3)  1 (9.1)  7 (63.6) | 5.44 | .066 |
| Accessing mental health support (T1) | Yes  No | 8 (16)  42 (84) | 1 (9.1)  10 (90.91) | 0.34 | .559 |
| Family history of  mental ill-health (T1) | Yes  No  I don’t know | 22 (44)  24 (48)  4 (8) | 5 (45.5)  6 (54.6)  0 | 0.96 | .618 |
