# Supplementary figures and images for: Evaluating the impact of sling provision and training upon maternal mental health, wellbeing and parenting: A randomised feasibility trial
Source: PLoS One. 2023 Nov 10;18(11):e0293501. doi: 10.1371/journal.pone.0293501 (PMC10637655; doi:10.1371/journal.pone.0293501)

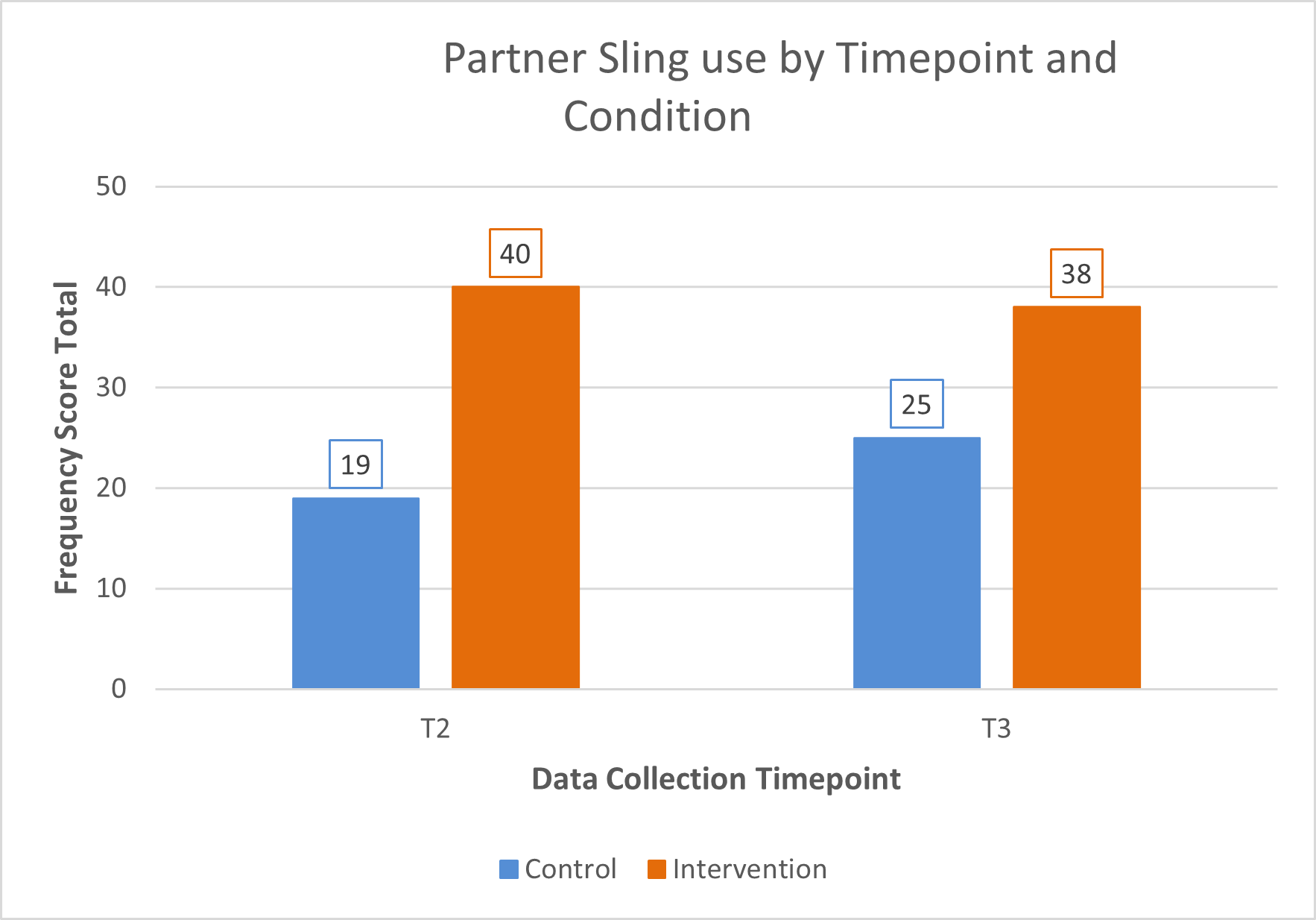

Supplement: S1 Fig — (TIF) [file pone.0293501.s002.tif]

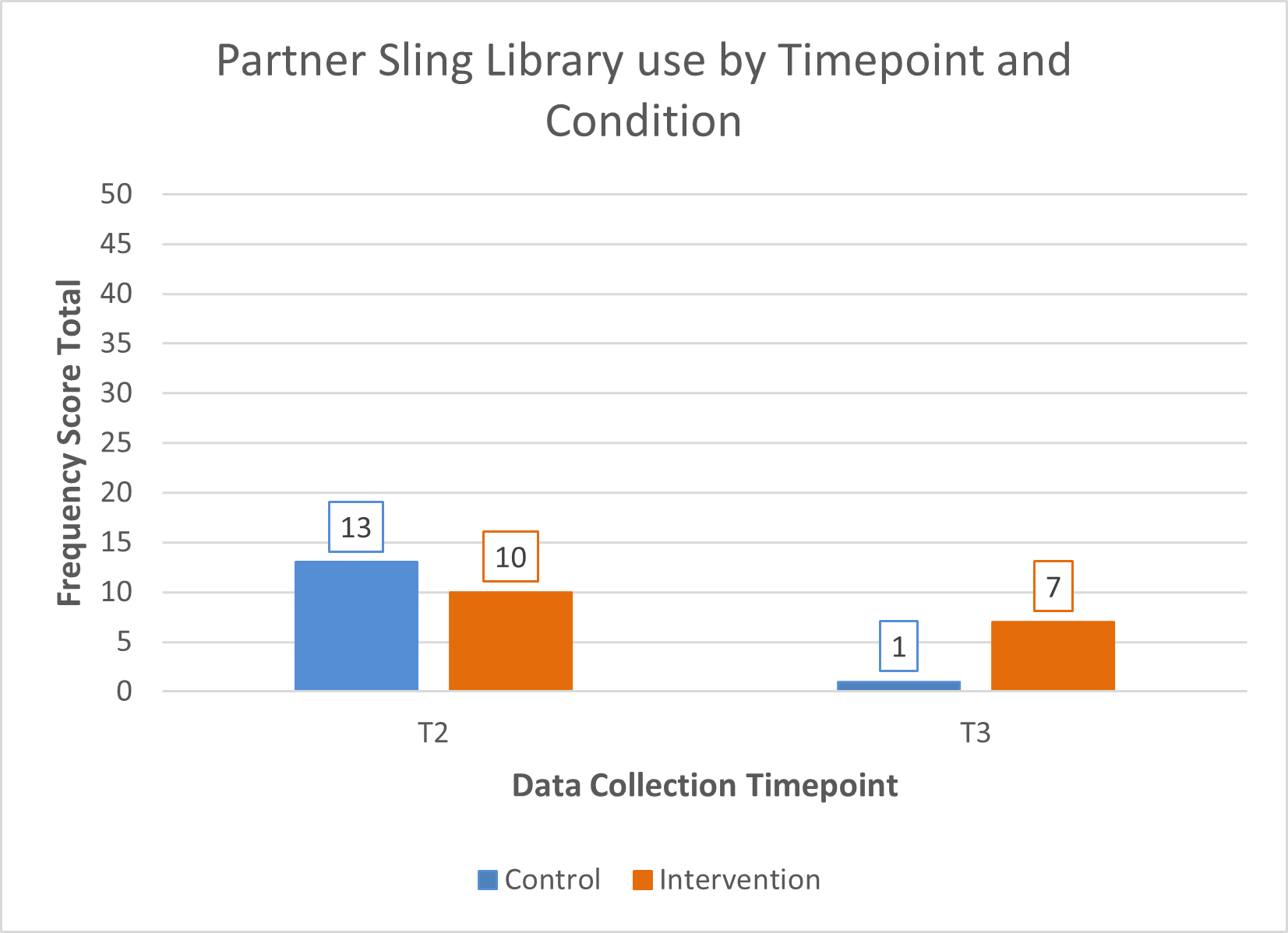

Supplement: S2 Fig — (TIF) [file pone.0293501.s003.tif]
