# Supplementary material for: Evaluating the impact of sling provision and training upon maternal mental health, wellbeing and parenting: A randomised feasibility trial
Source: PLoS One. 2023 Nov 10;18(11):e0293501. doi: 10.1371/journal.pone.0293501 (PMC10637655; doi:10.1371/journal.pone.0293501)
Supplement: S1 Protocol — (PDF) [file pone.0293501.s005.pdf]

RESEARCH PROTOCOL

Evaluating the Impact of Sling Provision and Training upon Maternal Wellbeing and Parenting: A Randomised Feasibility Trial

A quantitative study.

Version 3.

22/02/19

Trainee Clinical Psychologist:

Helen Wigglesworth

Research Supervisor:

Dr. Abigail Millings

Other Collaborators:

Dr. Rosie Knowles (Sheffield Sling Surgery and Library)

The National Childbirth Trust (NCT)

Contents

Abstract 3
Introduction 3
Clinical Implications 6
Aims and Hypotheses 7
Method 8
Measures 11
Procedure 21
Appendix A Recruitment Materials 46
Appendix B Items for All Time Points 47
Appendix C Edinburgh Post-natal Depression Scale 49
Appendix D Ten-point Supplement to EPDS Manual 50
Appendix E EPDS: Postnatal Depression Threshold Letter 51
Appendix F Depression Anxiety and Stress Scales-21 52
Appendix G Experiences in Close Relationships Scale-12 53
Appendix H Warwick Edinburgh Mental Wellbeing Scale 54
Appendix I Short Warwick Edinburgh Mental Well-Being Scale 55
Appendix J Parenting Sense of Competency Scale 56
Appendix K Items from the Caregiving Experiences Questionnaire 57
Appendix L Infant Behaviour Questionnaire-Revised Very Short Form 58
Appendix M Short Version of the Social Provisions Scale 62
Appendix N Maternal Stroking Questions 63
Appendix O Demographic Questions 64
Appendix P Qualitative Questions 68
Appendix Q Participant Information Sheet 69
Appendix R Participant Consent Form 74
Appendix S Templates of all Emails/Texts and Qualtrics Pages 76
Appendix T Drop-In Session Checklist 88
Appendix U Sling Surgery Materials and Emails 89
Appendix V Participant Debrief Sheet 95
Appendix W CONSORT 2010 Flow Diagram Template 98
Appendix X Timetable of Work 99
Appendix Y Project Costing 100

Abstract

Postnatal depression affects 10-15% of women worldwide (Cox, Murray, & Chapman, 1993). This randomised feasibility study investigates whether using an infant carrier leads to reductions in postnatal depression scores, increased wellbeing scores, and changes in parenting experiences. Maternal attachment style is examined as a potential moderator of this effect. Mothers of infants aged 0-6 weeks will be randomised to receive sling training and free sling hire, either immediately, or after a wait list period of 12 weeks (the control group). This study aims to collect information regarding the feasibility of recruitment and administering the intervention for future Randomized Control Trials.

Introduction

Worldwide, 10-15% of mothers suffer from postnatal depression (PND)(Cox et al., 1993), a form of depression where onset occurs soon after childbirth (Di Florio & Jones, 2018).

An even greater percentage of mothers (around 30%) may experience subthreshold depressive symptoms following childbirth. (Kingston et al., 2018). There is a need for low intensity interventions to mitigate these symptoms, and support mothers' well-being.

Biopsychosocial Perspective on PND

As with other depressive disorders, the biopsychosocial model (Engel, 1977) may be used to conceptualise the causes and maintaining factors of postnatal depression. This model proposes that biological, psychological and social factors are all interlinked and important in causing and maintaining illness (McInerney, 2002). Biologically, associations have been found between the functioning of the endocrine (hormone), immune and neurotransmitter systems, and the occurrence of PND (Harris, 1994).

Psychologically, women with PND tend to experience negative thoughts about themselves and specifically their ability to parent (Milgrom & McCloud, 1996). Negative patterns of thinking are key to the development and maintenance of depression (e.g. Beck's "cognitive triad"; Beck, 1967). Cognitive-based therapies typically involve building evidence that challenges negative thinking (Beck, 1979).

Finally, social isolation and social engagement are risk and protective factors, respectively, for depression (Thoits, 1985), including PND (Nielsen, Videbech, Hedegaard, Dalby, & Secher, 2005).

National Institute for health and Care Excellence (NICE) guidelines recommend psychotropic medication or high-intensity psychological interventions for those with moderate to severe PND (NICE, 2014). For those with mild to moderate symptoms, NICE recommend seeking healthy lifestyle advice, self-help programmes, computerised behavioural therapy, or exercise programmes (NICE, 2009). These various interventions may be onerous for mothers to access soon after giving birth (Bigelow, Power, MacLellan-Peters, Alex, & McDonald, 2012).

Evidence is emerging for alternative early intervention methods, including psycho-educational home visits (Ammaniti et al., 2006), parenting groups (Puckering, McIntosh, Hickey, & Longford, 2010), and baby massage (Onozawa, Glover, Adams, Modi, & Kumar, 2001), but these are not yet well-supported.

A low cost, low intensity intervention known to have beneficial effects on both maternal and infant wellbeing is close body contact (Winberg, 2005). Bigelow et al. (2012) found that mothers who provided regular skin-to-skin contact for the first month of their infant's life had lower depression scores than mothers in a control condition who provided little or no skin-to-skin contact.

One way in which a mother may increase close body contact with their infant, which may have similar benefits as skin-to-skin, is through the use of a "baby sling". This is a structured piece of fabric that allows the parent to carry their infant against their body (McGhee, n.d.).

Drawing from biopsychosocial models of depression, there are several mechanisms by which sling use might positively affect PND and maternal wellbeing.

Biological: Hormonal Effects

Close body contact between a mother and infant, such as that promoted by sling use, promotes the release of oxytocin in the mother (Uvnäs-Moberg & Prime, 2013). Oxytocin may reduce symptoms of those mental health difficulties which involve impaired social functioning, including depression (Cochran, Fallon, Hill, & Frazier, 2013; Domes, Normann, & Heinrichs, 2016).

Social: Autonomy and Social Support Effects

Self-determination theory notes that the absence of autonomy can lead to mental health difficulties (Deci & Ryan, 2008). Sling use allows the mother to have her hands free, and thus travel and carry out daily activities while holding her infant in close body contact (Blois, 2005). Sling use may therefore positively impact upon maternal wellbeing by providing this increased freedom and improving mothers' sense of autonomy, which in turn could reduce the risk of social isolation.

Additionally, sling use provides access to a range of social networks built around using slings (e.g. facebook groups and social meetings). Therefore sling use may also increase mothers' social opportunities and engagement.

Psychological: Infant-parent Relationship and Interaction Effects

An infant's attachment to the mother may contribute to the mother's experience of parenthood. For example, insecurely attached infants may cry more often than those who are securely attached to their parent (Belsky, Rovine, & Taylor, 1984).

Anisfeld et al., (1990) found that sling use may promote secure attachment in infants. Related to this, carrying has been found to reduce infant crying (Hunziker and Barr, 1986). Therefore, sling use may lead to a more positive parenting experience, building the number of positive interactions they experience, and consequently increasing feelings of parental self-efficacy (Jackson, 2000).

Altogether, by supporting each of these biopsychosocial factors, sling use may have a positive effect on symptoms of PND in mothers of young infants.

Parental “attachment style”, which refers to how the person approaches relationships later in life (Jones, Cassidy, & Shaver, 2015), may moderate the relationship between sling use and postnatal depression.

Parents with different attachment styles appear to differ in their thoughts and feelings about parenting (Millings, Walsh, Hepper, & O’Brien, 2013; Rholes, Simpson, & Friedman, 2006) and also about close physical contact (Posado, Waters, Crowell & Lay, 1995; Simpson, Rholes & Nelligan, 1992). Thus mothers with different attachment styles may respond differently to interventions for PND which involve such contact.

The aim of this study is to be the first to examine whether sling use may have a positive impact upon symptoms of postnatal depression, and furthermore, whether parental attachment style mediates this relationship.

Ideally, a randomised control trial (RCT) design would be used to establish a causal relationship between sling use and reduced occurrence of postnatal depression (Cartwright, 2010). To the researchers’ knowledge, there has not yet been an RCT that examines the impact of sling use on the parent specifically. However, before investing time and resources in a full-scale RCT, it is important to know whether it is possible to study the impact of this intervention (sling use) upon this population (mothers of young infants). In particular, it is anticipated that there may be challenges around recruitment and treatment fidelity.

For these reasons this study is a feasibility study, gathering information which may be used to estimate parameters (e.g. sample size, likely recruitment and drop-out rates) for future RCTs.

Clinical Implications

The primary clinical implication of this study is that, should sling use be found to reduce postnatal depression scores, then this lends evidence to the effectiveness of sling use as a possible early intervention for post-natal depression, perhaps reducing both the prevalence and impact of this condition.

Aims and Hypotheses

A feasibility study is underpowered to reliably assess treatment effectiveness (Williams, 2016). Therefore the primary focus of a feasibility study is not hypothesis testing, though statistical analysis of between-group outcome differences may be conducted in a preliminary way in order to inform the sample size calculations of future RCTs (Williams, 2016). Instead, as a feasibility study, the focus of this study is on collecting information that will support the design of future studies, e.g. recruitment rates, treatment adherence etc.

The primary aim of this study is therefore to examine whether it is possible to conduct an RCT with this population (mothers of new-born infants) and intervention (sling use, training and support). This study has a particular focus around the feasibility of recruitment of this population and also around treatment fidelity, including whether control group participants independently engage in sling use during the trial.

While this study is underpowered to draw definitive conclusions around treatment effectiveness, it is predicted that sling use will lead to lower Postnatal Depression scores (as measured by the Edinburgh Postnatal Depression Scale (Cox, Holden, & Sagovsky, 1987), and higher wellbeing scores (as measured by the Warwick Edinburgh Mental Wellbeing Scale (WEMWBS); Tennant et al., 2007), parenting self-efficacy and responsiveness (as measured by the Parenting Sense of Competency Scale (PSCS); Gibaud-Wallston & Wandersman, 1978; and Caregiving Experiences Questionnaire (CEQ); Brennan, George & Solomon, 2013), and breastfeeding frequency and duration, in the intervention group, compared to the control group. It is predicted that any improvement seen in the scores on these measures post-intervention, in comparison to pre-intervention scores, will be significantly greater for intervention group participants, than any improvement seen for control group participants.

A secondary hypothesis is that maternal attachment style (as measured by the Experiences in Close Relationships Scale-Short (ECR-S; Wei, Russell, Mallinckrodt & Vogel, 2007) might act as a moderator of the relationship between sling use and the scores on these various measures.

Method

Design

This study will use primarily quantitative methodology with an experimental design. As a feasibility study for a RCT, we will use the CONSORT guidelines for randomised pilot and feasibility trials (Eldridge et al., 2016) to inform decisions around sample size, methodology and reporting.

Organisations called "sling libraries" loan out slings and carriers, offer advice and information on safe and functional sling use, and allow users to connect with, and support, one another. Sheffield Sling Surgery runs one of the largest sling libraries in the UK (https://www.sheffieldslingsurgery.co.uk/). They have agreed to provide hire slings, and sling training and support, for the duration of this study.

There are many different types of sling available (e.g. ring-sling, stretchy-wrap, structured carrier etc.) in order to suit different body shapes, postures, infant weights etc. "Sling" in this study refers to all sling types.

Participants will be randomised to either the intervention group, in which participants will be provided with a sling and training and support in how to use it safely and comfortably, or a wait list control (WLC) group, in which participants will receive the intervention following the final data collection time-point. Data will be collected online at three time points (pre-, mid- and post-intervention), using self-report measures. These data will be quantitative, however, we

will also include a number of brief, open-ended questions at the final data collection time-point, and the resultant data will be analysed qualitatively.

Sample Size

With regards to sample size, as this is a feasibility study and hypothesis testing is not the primary aim, a normal power calculation need not be undertaken (Eldridge et al., 2016; Williams, 2016). Instead the sample size should be selected based on whether it can adequately estimate parameters that will support the design of future RCTs, e.g. recruitment rates, treatment adherence, completion rates of outcome measures etc. Furthermore it is common for the sample size of a feasibility study to be influenced by practical considerations, such as demands on time and resources (Eldridge et al., 2016).

With these considerations in mind, and following the recommendations of the National Institute of Health Research (NIHR: Research Designs Service; Hooper, 2014), it was estimated that a sample size of 50-60 participants (25-30 per condition) is likely to be both feasible and able to accurately estimate parameters for future RCTs, such as drop-out rates and standard deviations which could be used to determine future sample sizes.

Participants

Participants will be pregnant women with a due date that falls in the T1 data collection window (see below). Participants will be recruited via the following methods:

1) The national charity, The National Childbirth Trust will ask their Sheffield teachers to hand out our flyers at their group classes.
2) Flyers will also be distributed in locations likely to be frequented by expectant mothers. For example, shops selling baby-care products, community centres, libraries, hypnobirthing classes

etc.

Representatives from NCT have already agreed to assist with recruitment. Recruitment materials may be found in Appendix A.

Inclusion Criteria

This study aims to recruit expectant mothers whose due dates fall within the expected baseline data collection period (late February 2019 to July 2019).

This study aims to recruit women who have not already been a regular sling user (e.g. with a previous infant).

Participants will need to be able to travel to Sheffield Sling Surgery (Sheffield city centre) where the intervention will take place. This includes all participants, as those randomised to the control group will receive the intervention following trial completion.

Exclusion Criteria

Participants will be excluded from this study if they have used a sling previously or attended an antenatal workshop at a sling library. They will also be excluded if their infant has a chronic or serious illness, or a disability. This is because the sling library have advised that that parent-infant dyads for whom this is the case often require a greater level of support and training when using a sling than this study would be able to provide (Knowles, 2016). Any participants who are interested in the study, but get excluded on this basis, by definition meet the inclusion criteria for the charitable “Building Bonds” scheme, currently running at the sling library, offering free sling hire, training and support specifically for families with chronic conditions and disabilities. These individuals would be referred to this scheme, and so will not be disadvantaged by the exclusion criteria.

As it is a feasibility study, this study has few inclusion and exclusion criteria. However data will be collected regarding any mental health diagnoses, number of children prior to their current infant, participant age, socioeconomic status, ethnicity, education level, postcode, and whether they are in a relationship or not. During data analysis, the impact of these factors upon the results of the study will be examined in order to potentially inform inclusion and exclusion criteria of future RCTs.

Service User Involvement

Collaborators for this study include the National Childbirth Trust (NCT), as well as the local sling library.

The NCT provides independent information to parents, and also helps parents to connect with local parent support groups in order to gain both practical and emotional support (NCT, n.d.). The NCT have agreed to assist in the recruitment of participants for this study who are not already sling users.

This project has developed, in part, through pre-existing collaboration between the project supervisor and the local sling surgery and library. The sling surgery has helped to develop the design of this project from its earliest stages.

Furthermore, it is hoped that, through these organisations, it will be possible to send an example battery of measures to both sling users and non-sling users in order to receive feedback around whether the battery as it stands looks too onerous, or time consuming, for mothers who have recently given birth, to complete.

Measures

All measures are self-report and will be completed online using the programme

Qualtrics.

At this stage in the research process a number of measures have been selected to be administered to participants. As mentioned in the "Service User Involvement" section above, it is hoped that service users might feedback on whether, as it stands, this battery of measures is currently too onerous for participants to complete, particularly as baseline measures are due to be completed soon after childbirth. Thus while the full battery of possible measures is presented here, some of these measures may be omitted from the final battery administered to participants, based on service user feedback as well as the judgement of the researchers and staff at the sling surgery.

Primary Measures

Sling Use and Support

With the support of the sling surgery, as in previous studies (Anisfeld et al., 1990), an idiographic measure has been designed, asking participants how often they have used their sling over the past 6 weeks (please see Appendix B).

For comparison, participants will also be asked how often they have used a pram or stroller over the same time period.

This measure also asks participants how often they have accessed the services of the Sling Surgery over the past 6 weeks, or any other sling-related support.

Participants will be asked how often their partner has used a sling, pram, or accessed the Sling Surgery Services over the past 6 weeks, so that this information may be taken into account. This is partly in order to account for partner sling use as a possible confounding variable when analysing the results of the study, but it also acts as a way of capturing paternal involvement and use of slings, which may be of interest in future research.

At T2 and T3, participants will be asked whether they have swapped their sling over the past 6 weeks (the course of the study since the previous data collection time-point), and if so, how often they have done so.

The time period which all of the above questions refer to (6 weeks) may be altered according to feedback from the Sling Surgery and service users.

Postnatal Depression

Our primary measure is the Edinburgh Postnatal Depression Scale (EPDS; Cox, Holden & Sagovsky, 1987)(Appendix C) . The EPDS is a 10 item self-report scale designed to screen for postnatal depression in nonclinical populations. Participants give one of four possible responses to each of the ten statements. These possible responses indicate frequency, e.g. ranging from “Not at all” to “Yes, most of the time”, but the exact wording varies according to the statement. The scale typically takes around 5 minutes to complete (Cox et al., 1987).

The EPDS has been used widely in the National Health Service (NHS) as a screening tool for post-natal depression; and in previous studies in the field of maternal mental health and wellbeing (Leahy-Warren, McCarthy & Corcoran, 2012). A number of past studies found this scale to be a valid and reliable (Cox & Holden, 1994; Pope & Pope, 2000). In 2017, Cox published a 10 point supplement to their previous manual for this scale, designed to assist researchers in avoiding misuse of the EPDS (Cox, 2017). This study aims to follow both the manual for the scale (Cox, Holden & Henshaw, 2014), and this supplement (see Appendix D), in order to ensure standardised practice.

Because this measure is the primary screening tool for PND used in the NHS, if a participant scores above the clinical threshold implemented in the NHS, they will be sent a letter informing them of this, and encouraging them to access assessment and treatment for

postnatal depression via their GP or health visitor. This letter can be found in Appendix E.

At all three data collection time-points, information will be collected around whether participants are currently accessing pharmacotherapy or psychological care, using specific mental health-related items (please see Appendix B). This is in order to control for this variable during data analysis.

Recent studies have found that the sensitivity and specificity of the EPDS can vary between 59% to 100% and 44% to 97% (respectively) according to the geographic location of the study, the cutoff scores chosen by researchers, and the reference standards used for diagnosis (Novotney & Maurer, 2017).

Due to this variability in terms of sensitivity and specificity, some studies have recommended supplementing the EPDS with a second validated measure of depression that has shown less variation in sensitivity and specificity (Miller, Pallant, & Negri, 2006).

While a number of studies have used the Beck Depression Inventory (BDI; Beck et al., 1961) or Patient Health Questionnaire (PHQ-9; Kroenke, Spitzer, & Williams, 2001) measures in this way (Moraes, Lorenzo, Pontes, Montenegro, & Cantilino, 2017), Miller et al., suggest that these measures are not valid for use with a postnatal population, as often the items included in these measures are experiences that commonly occur following birth (e.g. increased tiredness, change in eating patterns, change in sleeping).

For this reason Miller et al., elected to supplement the EPDS with the Depression Anxiety and Stress Scales (DASS; Lovibond & Lovibond, 1995). The DASS does not include these items that have been identified as potentially confounding within measures such as the BDI (Miller et al., 2006). It was originally designed as a measure that distinguished between symptoms of Anxiety and those of Depression, and subsequently was found to also identify a third symptom group, that the authors labelled as “stress” (Lovibond & Lovibond, 1995). The

DASS has established reliability and validity with both non-clinical and clinical populations (Ng et al., 2007). Miller et al., argue that the DASS offers "a more complete picture" of maternal mood following childbirth, because it is a general measure of "postnatal distress", including anxiety and stress rather than focusing on depression alone. Furthermore this measure has been found to be sensitive to mild presentations of these conditions, thus not only capturing those with severe symptomatology and instead identifying a range of levels of distress.

The original DASS consists of 42 statements. Participants indicate the degree to which they feel each statement has applied to themselves over the last week, using a four-point Likert scale ranging from "Did not apply to me at all" to "Applied to me very much, or most of the time". For the sake of brevity, this study will be using the DASS-21(Appendix F), a version of the DASS that includes only 21 statements. The DASS-21 has been widely used with both clinical and non-clinical populations and has been found to have strong concurrent validity with other measures, such as the BDI, and the Beck Anxiety Inventory (Beck, Epstein, Brown & Steer, 1988)(Le et al., 2017).

Attachment

We will use the Experiences in Close Relationships Scale (12 item version; ECR-12) (Lafontaine et al., 2015) (see Appendix G) as a measure of attachment. This is a 12-item version of the original 36-item Experiences in Close Relationships Scale (ECR) (Brennan, Clark & Shaver, 1998). The ECR and ECR-S are used to evaluate adult attachment style. They contain 12 and 36 statements respectively, which participants rate from 1 (strongly disagree) to 7 (strongly agree) using a 7-point likert scale. Both produce two scores labelled *Anxiety* and *Avoidance.* The ECR is a well-validated and reliable measure, and the ECR-S has been found to have validity equivalent to that of the ECR (Wei et al., 2007).

Secondary Measures

Wellbeing

This study aims to examine the impact of sling use upon both maternal mental health and maternal wellbeing. Currently, mental wellbeing is seen as more than the absence of mental health difficulties ("Promotion of mental well-being", n.d.), with these constructs viewed as separate rather than two ends of the same scale (Tennant et al., 2007). Thus it seemed appropriate to also include a separate measure of wellbeing to administer alongside the DASS and EPDS.

The Warwick Edinburgh Mental Wellbeing Scale (WEMWBS; Tennant et al., 2007) is a 14-item scale designed to measure and monitor mental wellbeing in the general population ("Warwick Edinburgh Mental Wellbeing Scale (WEMWBS)", n.d.). The items are worded positively, with participants rating each statement on a five-point Likert scale from "None of the time" to "All the time".

The WEMWBS has been validated for use with people aged 13 to 74 in both clinical and non-clinical populations. (Tennant, et al.,2007; Clarke, et al., 2011; Stewart-Brown et al., 2009; Maheswaran, Weich, Powell, & Stewart-Brown, 2012; Stewart-Brown, 2013). Crawford et al., (2011) found that service users find the WEMWBS easy to complete and prefer this scale over other mental health scales. The WEMWBS has been used previously with mothers who have recently given birth (McKellar, Steen & Lorensuhewa, 2017; Mustaffa, Marappan, Abu, Khan, & Ahmad, 2014). Therefore this measure will be used as a primary measure of maternal wellbeing within this study (Appendix H).

There is a short 7-item version of the WEMWBS (the SWEMWBS, Appendix I),

which may be used in this study if the battery of measures becomes too onerous. This version has been found to have adequate internal consistency and reliability (Haver, Akerjordet, Caputi, Furunes, & Magee, 2015).

Parenting

The Parenting Sense of Competency Scale (PSCS; Gibaud-Wallston & Wandersman, 1978)(Appendix J) will be used to measure parental self-efficacy. This scale measures two dimensions of parental competence: Satisfaction and Efficacy. It is a 16-item likert-scale questionnaire in which participants give responses ranging from “strongly agree” (1) to “strongly disagree” (6). There are nine questions for the dimension of Satisfaction (examining the parents’ perceived anxiety, motivation and frustration), and seven assessing Efficacy (parents’ perceived competence, capability and problem-solving abilities). This scale has been validated (Ohan, Leung, & Johnston, 2000).

Following the example of Cutrona & Troutman (1986), and again, following feedback from service users, this study may, for the sake of brevity, use only the seven Efficacy items.

The Caregiving Experiences Questionnaire (CEQ; Brennan, George & Solomon, 2013) will also be used as a parenting measure within this study, assessing the nature of each participating mother’s relationship with their child. The CEQ is a measure designed to assess 5 dimensions of the parent-child relationship. These dimensions have emerged from attachment theory and include “enjoyment, discourages closeness, heightened, helplessness, and role reversal” (Brennan et al., 2013). Each item of this scale is a statement designed to examine a mother’s evaluations of themselves, their child, and their relationship with their child. Altogether the CEQ contains 40 of these statements (Appendix K), with mothers asked

to endorse items using a five-point Likert scale ranging from “Not at all characteristic” (1) to “Very characteristic” (5). The CEQ was developed and validated in a US population. Research investigating the validity of this measure in non-US populations is ongoing (Røhder et al., 2018).

Lastly, a measure of infant stroking by mothers has been included in the battery of questionnaires. This is because recent literature has highlighted possible associations between maternal mental health and maternal stroking (Sharp, Pickles, Meaney, Marshall, Tibu, & Hill, 2012). A measure developed by Sharp et al. (2012), which was designed for use in the community, will be used in this study also (please see Appendix N).

Infant Temperament

This study will use the Infant Behaviour Questionnaire-Revised Very Short Form (IBQ-R VSF; Putnam, Helbig, Gartstein, Rothbart, & Leerkes, 2014) to measure infant temperament. The original Infant Behaviour Questionnaire (IBQ; Rothbart, 1981) consisted of six scales assessing “Activity Level, Fear, Distress to Limitations, Smiling and Laughter, Soothability, and Duration of Orienting”. The revised version (IBQ-R; Gartstein & Rothbart, 2003) added several new scales (“Approach, Vocal

Reactivity, High and Low Intensity Pleasure, Perceptual Sensitivity, Sadness, Falling Reactivity, and Cuddliness”) resulting in a measure that consisted of 191 items in total. Putnam et al., recognised that, while it may be important to capture the wide range of characteristics that may be regarded as aspects of temperament, there is a limit to the demands that researchers may place upon participants in terms of both time and effort. In order to “resolve this conflict”, Putnam et al., developed the IBQ-R Short Form (91 items total) and the IBQ-R VSF (37 items total).

Due to the number of measures already included in the batteries that will be administered to our participants at each time-point, this study has opted to use the IBQ-R VSF (Appendix L). This version of the IBQ assesses three broader factors involved in infant temperament: Negative Emotionality (NEG), Positive Affectivity/Surgency (PAS), and Orienting/Regulatory Capacity (ORC). The 14 “fine-grained” scales examined in the IBQ-R make up these wider factors. For example, Putnam et al., propose that NEG is made up of the Sadness, Distress to Limitations, Fear, and Falling Reactivity scales, while PAS consists of the Approach, Vocal Reactivity, High Intensity Pleasure, Smiling and Laughter, Activity Level, and Perceptual Sensitivity scales. Finally ORC is made up of the Duration of Orienting, Low Intensity Pleasure, Cuddliness, and Soothability scales.

In all versions of the IBQ, participants are asked to use a seven-point Likert scale, ranging from “Never”(1) to “Always”(7), to indicate the frequency with which their child has displayed specific behaviours within certain situations (e.g. “When tired, how often did your baby show distress?”) over the past week. The scale also includes a “Not Applicable” option in case one (or more) of the situations described, had not arisen over the past week.

With regards to validity, the IBQ and IBQ-R are two of the most frequently used measures of infant temperament (Putnam et al., 2014). A number of studies have found these measures to have validity equal to observational measures of infant temperament (Gartstein et al., 2010; Gartstein & Marmion, 2008; Kochanska, Coy, Tjebkes, & Husarek, 1998; Parade & Leerkes, 2008). Peterson et al., (2017) found that the IBQ-R VSF has “promising reliability and predictive validity in a large, diverse sample.”

Perceived Social Support

A shortened, 5-item, version of the Social Provisions Scale (SPS; Russell & Cutrona, 1984), which has been used in previous studies and shown to correlate with the full, validated, SPS (Cutrona & Troutman, 1986) will be used to measure perceived social support. The SPS measures the six aspects of social support identified by Weiss (1974). Each item is a statement (specified in Cutrona & Troutman, 1986; please see Appendix M), which participants rate on a 7-point likert scale, from “Not at all true” to “Completely true”.

Demographic Measures

Data will be collected around the following:

Age, postcode, ethnicity, mental health, physical health, first born vs. latter born, how they have heard about the study, socioeconomic status, whether the person has a partner, and years in education.

Please see Appendix O**.**

Breastfeeding

Information will be collected around mothers’ feeding methods (i.e. whether they are breastfeeding or tend to formula feed) over the course of the study. This breastfeeding item has been integrated into the demographics questionnaires, but, unlike the demographic questions, will be re-administered at the final data collection time-point (Please see Appendix O).

Qualitative Questions

Qualitative data is commonly collected in feasibility studies, as it can help to improve the design of future trials, including intervention content, and the selection of relevant outcome measures in order to support greater measurement completion (O’Cathain et al.,

2015).

Therefore, at T3, all participants will be given qualitative questions about their experience of participating in the study, and their experience of using a sling and accessing the Sling Surgery services, to answer online. These questions will be phrased as open questions and will be formatted as free-text boxes on Qualtrics, rather than having any set response options. They may be found in Appendix P.

The exact wording of these questions may change following service user feedback.

Procedure

Participants will be recruited while they are pregnant. When recruited, participants will be provided with further information, complete consent forms and provide contact details via the software programme Qualtrics (please see Appendices P-R), then be randomised to one of two conditions: an intervention condition or a waitlist control condition. This randomisation will not be blind.

Participants will be allocated to each condition using a computer generated randomisation list. This will follow a 1:1 randomisation ratio (1 intervention participant per each control participant) so that there are equal numbers of participants allocated to each condition.

Once they have consented to participating, participants will be sent an email containing a link to the online baseline measures. This email will ask them to complete these measures when their child is between 0 and 6 weeks of age.

Participants will be sent a text reminder around the time of their due date (unless they have requested an email or no reminder instead), reminding them to complete these measures.

This text or email will again contain the link to the baseline measures in case they no longer have access to their first email.

Example templates of the emails and/or texts that may be sent to participants, as well as the information that may be shown to participants via Qualtrics when they are completing each battery of measures at each time-point, may be found in Appendix S.

Intervention

Upon completion of the baseline measures, participants allocated to the intervention condition will be invited (via Qualtrics and an email “triggered” when measures are completed on Qualtrics) to attend a drop-in session at the sling surgery, on a Thursday, Friday or Saturday, while their infant is between 0 and 6 weeks of age. Participants will be asked to book on to a session in order to monitor demand for free slings from the Sling Surgery and ensure that slings that have been put aside for use by the study are available on the day that the participant attends.

A window of 6 weeks has been given, as recommended by the sling surgery, as the researchers recognise that each birthing experience is different and mothers may vary in the time it takes for them to feel able to undertake a trip to the sling library following giving birth.

The drop-in sessions that participants will be invited to attend are those run regularly by the Sling Surgery, and are open to the public. Typically parents are able to attend these sessions at any point during the two-hour period that they run and are welcome to stay for as long as they like within this time period. Parents attend these sessions seeking advice for slings that they are currently using, or the opportunity to try using a sling for the first time before buying

or hiring.

Participants will be instructed to identify themselves as a participant in the study when they attend their initial session, and whenever they otherwise interact with the Sling Surgery. This is so that their data is stored correctly and so that they are not accidentally charged for any of the Sling Surgery services.

Furthermore, the research team has created a checklist (based on the standard drop-in session procedure already in place) to be used during sessions with study participants. This is in order to support standardisation of session content across each participants' sessions, and also to make the study more replicable. This checklist may be found in Appendix T.
By identifying themselves as study participants, participants will ensure they are given a session using this checklist.

Typically a parent or parents will sign-in, giving their name, email address, postcode, how they have travelled to the session, their child's name and age, and whether they require support that day). They will then be greeted by either a sling consultants or a voluntary peer supporter. On busy days there may be a short wait before someone is available to greet and work with the parent. At this point participants will state that they are a participant in this study. Following the session checklist, participants will be offered sling training and advice, and a sling use demonstration. Participants will learn how to safely use one of three different types of sling (either a stretchy, a close caboo, or one type of buckle carrier, dependent on the parent and infant's needs and preference) with a weighted demonstration doll before practicing with their own baby. Participants will be given this sling to hire for free for the duration of the study and a reminder leaflet containing sling safety information (Appendix U).

In these drop-in sessions, rather than being private 1:1 consultations, all contact between parents and Sling Surgery staff takes place within the same room. Thus while each parent, or set of parents, is met individually, if a number of parents have the same question, or a number are participants in this study, a Sling Surgery staff member may end up training, advising and demonstrating to a group of parents, rather than providing 1:1 support.
Because everything takes place within the one room (aside from completion of the paperwork for hiring or buying a sling), this offers parents the opportunity to meet and support each other, and in particular discuss their experiences of sling use so far.

Following this session, participants will be sent an email which contains a link to a webpage with reminder information about their hire sling and how to use it, as well as an invitation to join the online sling community for further support, and information about the other support available through the sling surgery (Appendix U). Staff at the Sling Surgery will create a new "library" on their myTurn database in order to send these emails which are tailored to this study (e.g. which do not mention hire fees etc.).

Throughout the study, participants will be able to access the sling surgery's support services as regularly as they like, including those that do not focus specifically on sling use. Participants will also be able to swap their sling if they suspect that another sling may be more suited to themselves and their infant. This is in line with the sling surgery's current standard practice.

Control Participants

Control participants will be asked to complete the same measures as participants in the intervention condition, at the same time points, again via email or text depending on their stated preferred method of communication. However control participants will not be offered the intervention (sling hire, training and support) when their infant is between 0 and 6 weeks old, but instead will be offered one month's free sling hire, training and support, following completion of the study. Via email, which will be triggered when participants complete their final set of measures on Qualtrics, participants will be asked to book on to a session in order to monitor demand for free slings from the Sling Surgery and ensure that slings that have been put aside for use by the study are available on the day that the participant attends.

Data Collection

When their infant is 0 to 6 weeks old participants will complete baseline measures. For intervention participants completion of baseline measures will be prior to attending the sling surgery session. Currently these are due to include measures of PND (EPDS, DASS21), attachment (ECR-S), a measure of sling and buggy use and also of use of sling surgery services, a breastfeeding measure, and measures of wellbeing (WEMWBS/SWEMWBS), parenting (PSCS, CEQ), infant temperament (IBQ-R VSF) perceived social support (SPS) and general demographics (as mentioned in the *participants* section above).

As part of this feasibility study, it would be helpful to examine how long is needed before an effect of sling use upon maternal mental health, wellbeing and parenting may be seen. This information could support future RCTs in deciding the duration of a sling use intervention condition. It was agreed that an additional time-point, midway through the study, may help to narrow down the point at which an effect of sling use may be seen. Thus, 6 weeks

after completion of baseline measures, both intervention and control participants will be asked (via text or email - see below) to complete Time-2 (T2) measures online. These T2 measures are not the full battery of measures. Rather, participants in both groups will be asked about current sling use and use of sling surgery services (including whether they have swapped their sling). The EPDS will also be administered a second time at this time-point.

A further 6 weeks later (T3) participants in both conditions will be asked (via text or email - see below) to complete for a second time the full battery of measures that had been given at baseline (T1) (excluding demographic measures).

A text or email reminder (depending on each participant's stated preferred method of contact) will be sent each time that the participant is due to complete their next battery of measures. This message will contain a link to these measures (via Qualtrics).

Debrief

Participants in both conditions will be debriefed following completion of measures at time-point 3. This will be sent via an email which will be triggered by Qualtrics when this final set of questionnaires is completed by the participant. For intervention participants, the email will also remind them to now return their sling, while, for control participants, the email will ask them to book on to a session at the Sling Surgery so that they may receive their sling training and begin their free sling hire. Examples of these emails may be found in Appendix S. A copy of the debrief sheet may be found in Appendix V.

Data Security and Management

Data will be stored using Qualtrics. Using this programme, the data is password protected and only accessible by the research team. Identifiable data will need to be kept, as participants will need to be reminded to complete T2 and T3 measures via phone or email (whichever is their preferred method) at the correct times, and data analysis relies on matching participants' data from each time point.

Contact details, as well as information about each participant's sling hire and any other access to Sling Surgery services will be securely stored on both Qualtrics and the sling surgery myTurn hire system, which is also password protected and only accessible by Sling Surgery staff. This will allow the sling surgery to follow their standard practice and keep track of where and when slings have been hired out.

Only secure university or Sling Surgery email addresses will be used to contact participants and the sim card used to contact participants will be kept in a locked location only accessible by research team members.

Proposed Analysis

As a feasibility study it is difficult to predict participant drop-out rate, and the impact that this may have on the results of the study. In order to control for drop-out, intention-to-treat (ITT) analysis will be used.

A mixed ANCOVA will be used to evaluate the relationship between sling provision and maternal mental health, wellbeing and parenting. This will include a comparison of both within-group pre- and post-intervention scores, and a between-group comparison of intervention vs. control group scores.

Attachment will be included as two covariates, as the measure of attachment used in this study produces two scores (*Avoidance* and *Anxiety*).

Thematic analysis will be used to evaluate the data collected from the two qualitative questions included at T3.

As a feasibility study, the impact of treatment fidelity on the results of the study is of interest. In order to examine this impact, it may be helpful to run the analysis again, this time excluding data from control participants who have used a sling, or accessed the sling surgery services over the course of the study. Similarly, it may be helpful to then re-run the analysis again, but this time excluding participants in the intervention condition who did not use their sling regularly. Through a similar process, it may be important to re-analyse the results of the study, investigating whether the frequency of participants' partners' sling use impacts upon these results.

It will be important to investigate whether the results of various demographic groups differ, in order to inform the inclusion or exclusion criteria of future RCTs. Frequencies and descriptive statistics derived from the demographic measures will be examined and presented. If possible, tests of difference will be conducted using this data, though this will of course depend upon the size of the demographic groups.

Reliability and Validity Checks

As mentioned above, in part in order to improve replicability (and thus reliability) of this study, a session content checklist will be used during the sling intervention session, so that the same intervention may be give both across participants and across studies should future

studies utilise this checklist.

In order to support the replicability and validity of this study, a CONSORT flow diagram (Moher, Schulz, & Altman, 2001) will be included in the reporting of the study (please see Appendix W).

This study aims to use only measures that have been validated and found to be reliable by past studies. Both intervention and control participants will be recruited from the same population. The intervention used in this study is representative of the intervention that a parent may typically receive from the service in which the study takes place (the sling surgery). Furthermore the study will take place in the usual setting, and with the usual staff, that such an intervention would normally involve.

Ethical Implications

Ethical approval will be sought from the University of Sheffield.
As mentioned in the *Procedure* section above, during recruitment, participants will be given information explaining the aims and procedure of the study (please see Appendix Q). They will be asked to complete a consent form prior to participating in the study (Appendix R). It will be made clear that participants are free to withdraw their data at any time. Participants will also be debriefed at the end of the study (Appendix V).

As mentioned in the *Primary Measures* section above, during the study, it may be that participants score above the clinical threshold applied in NHS settings on the EPDS. Should this happen, participants will be sent a letter (please see Appendix E) recommending that they speak to their midwife or health visitor, and also their GP. The letter will be marked “Private and Confidential”.

It must be acknowledged that there is an incentive for participation in this study, in that participants in both conditions will receive sling hire and training for free, that they might have otherwise paid for. Furthermore, while control participants will receive the intervention (sling hire, training and support) following completion of the study, they will receive one month of free sling hire, support and training, the intervention participants will have received three months of free sling hire, support and training over the course of the study. While this discrepancy may appear unfair, it is commensurate with sling library advice and based on the different level of support needed in relation to infant age. Parents of very young infants usually require more support with sling use than parents of older infants.

Timetable/ Plan of work

Following application for ethical approval in December 2018, it is hoped that recruitment will begin in February 2019.

Baseline measures (T1) will be collected from participants in both conditions, between late February 2019 and late July 2019. The aim will therefore be, to recruit participants whose due date falls within this period. Participants in the intervention condition will attend their session at the sling surgery during this period, once they have completed their baseline measures.

Following this, participants will complete T2 measures between April 2019 and mid-September 2019, and T3 measures between mid-May 2019 and late October 2019.

Following completion of data collection (from mid-May onwards, dependent upon when the participant completed their baseline measures), control participants will be offered one month's free sling hire, a session at the sling surgery and access to other support at the sling surgery.

It is hoped that data analysis may be completed by December 2019, ready for the results to be written up and submitted by May 2020.

This timetable is displayed in a Gantt chart in Appendix X.

Justification for Proposed Costs

Proposed costs of the study are detailed in Appendix Y. These costs are for the printing of flyers for recruitment, and the printing of any letters to participants, should they score highly on the EPDS (see "Ethical Implications" section above). Included in the cost is a pay-as-you-go sim card, in order to communicate with participants via text if this is their preference.

In order to reduce costs, the majority of communication with participants (participant information, consent forms, sling surgery information) and the administration of measures, will be done electronically.

References

Alvarez, S., Meltzer-Brody, S., Mandel, M., & Beeber, L. (2015). Maternal Depression and Early Intervention. *Infants & Young Children*, *28*(1), 72-87. https://dx.doi.org/10.1097/iyc.0000000000000024

Ammaniti, M., Speranza, A., Tambelli, R., Muscetta, S., Lucarelli, L., & Vismara, L. et al. (2006). A prevention and promotion intervention program in the field of mother–infant

relationship. *Infant Mental Health Journal*, *27*, 70-90. https://doi.org/ 10.1002/imhj.20081

Anisfeld, E., Casper, V., Nozyce, M., & Cunningham, N. (1990). Does Infant Carrying Promote Attachment? An Experimental Study of the Effects of Increased Physical Contact on the Development of Attachment. *Child Development*, *61*, 1617. https://doi.org/10.1111/j.1467-8624.1990.tb02888.x

Beck, A. T. (1967). *Depression: Clinical, experimental, and theoretical aspects*. University of Pennsylvania Press.

Beck, A. (1979). *Cognitive therapy of depression*. New York: Guilford Press.

Beck, A.T., Epstein, N., Brown, G., & Steer, R.A. (1988). An inventory for measuring clinical anxiety: Psychometric properties. *Journal of Consulting and Clinical Psychology, 56,* 893-897. https://doi.org/10.1037/0022-006X.56.6.893

Beck, A. T., Ward, C. H., Mendelson, M., Mock, J., & Erbaugh, J. (1961). An inventory for measuring depression. *Archives of general psychiatry*, *4*, 561-571. https://doi.org/10.1001/archpsyc.1961.01710120031004

Bigelow, A., Power, M., MacLellan-Peters, J., Alex, M., & McDonald, C. (2012). Effect of Mother/Infant Skin-to-Skin Contact on Postpartum Depressive Symptoms and Maternal Physiological Stress. *Journal Of Obstetric, Gynecologic & Neonatal Nursing*, *41*, 369-382. https://doi.org/10.1111/j.1552-6909.2012.01350.x

Blois, M. G. (2005). *Babywearing: the benefits and beauty of this ancient tradition*. Plano, TX: Hale

Publishing.

Brennan, K. A., Clark, C. L., & Shaver, P. R. (1998). Self-report measurement of adult attachment: An integrative overview. In J. A. Simpson & W. S. Rholes (Eds.), *Attachment theory and close relationships* (pp. 46-76). New York, NY, US: Guilford Press.

Brennan, J., George, C., & Solomon, J. (2013). The Caregiving Experiences Questionnaire. *Age*, *1*, 2-5.

Cartwright, N. (2010). What are randomised controlled trials good for?. *Philosophical studies*, *147*, 59. https://doi.org/10.1007/s11098-009-9450-2

Clarke, A., Friede, T., Putz, R., Ashdown, J., Martin, S., & Blake, A. et al. (2011). Warwick-Edinburgh Mental Well-being Scale (WEMWBS): Validated for teenage school students in England and Scotland. A mixed methods assessment. *BMC Public Health*, *11*. https://doi.org/10.1186/1471-2458-11-487

Cochran, D., Fallon, D., Hill, M., & Frazier, J. (2013). The Role of Oxytocin in Psychiatric Disorders. *Harvard Review Of Psychiatry*, *21*, 219-247. https://doi.org/10.1097/hrp.0b013e3182a75b7d

Cox, J. (2017). Use and misuse of the Edinburgh Postnatal Depression Scale (EPDS): a ten point ‘survival analysis’. *Archives of Women’s Mental Health,* 20, 789-790. https://doi.org/10.1007/s00737-017-0789-7

Cox, J. L., & Holden, J. (Eds.). (1994). *Perinatal psychiatry: use and misuse of the Edinburgh Postnatal Depression Scale*. Gaskell.

Cox, J., Holden, J., & Henshaw, C. (2014). *Perinatal Mental Health: The Edinburgh Postnatal Depression Scale (EPDS) Manual (2nd edn)*. RCPsych Publications.

Cox, J., Holden, J., & Sagovsky, R. (1987). Detection of Postnatal Depression. *British Journal Of Psychiatry*, *150*, 782-786. https://doi.org/10.1192/bjp.150.6.782

Cox, J., Murray, D., & Chapman, G. (1993). A Controlled Study of the Onset, Duration and Prevalence of Postnatal Depression. *British Journal Of Psychiatry*, *163*, 27-31. https://doi.org/10.1192/bjp.163.1.27

Crawford, M., Robotham, D., Thana, L., Patterson, S., Weaver, T., & Barber, R. et al. (2011). Selecting outcome measures in mental health: the views of service users. *Journal Of Mental Health*, *20*, 336-346. https://doi.org/10.3109/09638237.2011.577114

Cutrona, C. E., & Troutman, B. R. (1986). Social support, infant temperament, and parenting self-efficacy: A mediational model of postpartum depression. *Child development*, 1507-1518. https://doi.org/10.2307/1130428

Deci, E. L., & Ryan, R. M. (2008). Self-determination theory: A macrotheory of human motivation, development, and health. *Canadian psychology/Psychologie canadienne*, *49*, 182. http://dx.doi.org/10.1037/a0012801

Di Florio, A., & Jones, I. (2018, May). Postnatal depression. Retrieved November, 2018, from https://bestpractice.bmj.com/topics/en-gb/512

Domes, G., Normann, C., & Heinrichs, M. (2016). The effect of oxytocin on attention to angry and happy faces in chronic depression. *BMC Psychiatry*, *16*. https://doi.org/10.1186/s12888-016-0794-9

Eldridge, S., Chan, C., Campbell, M., Bond, C., Hopewell, S., Thabane, L., & Lancaster, G. (2016). CONSORT 2010 statement: extension to randomised pilot and feasibility trials. *BMJ*, i5239. https://doi.org/10.1186/s40814-016-0105-8

Engel, G. (1977). The need for a new medical model: a challenge for biomedicine. *Science*, *196*(4286), 129-136. https://doi.org/10.1126/science.847460

Gartstein, M. A., Bridgett, D. J., Rothbart, M. K., Robertson, C., Iddins, E., Ramsay, K., & Schlect, S. (2010). A latent growth examination of fear development in infancy: Contributions of maternal depression and the risk for toddler anxiety. *Developmental Psychology*, *46*, 651. https://doi.org/10.1037/a0018898

Gartstein, M. A., & Marmion, J. (2008). Fear and positive affectivity in infancy: Convergence/discrepancy between parent-report and laboratory-based indicators. *Infant Behavior and Development*, *31*, 227-238. https://doi.org/10.1016/j.infbeh.2007.10.012

Gartstein, M. A., & Rothbart, M. K. (2003). Studying infant temperament via the Revised Infant Behavior Questionnaire. *Infant Behavior and Development, 26*, 64-86. https://doi.org/10.1016/S0163-6383(02)00169-8

Gibaud-Wallston, J., & Wandersman, L. P. (1978). *Parenting sense of competence scale*. Lawrence Erlbaum Associates.

Harris, B. (1994). Biological and Hormonal Aspects of Postpartum Depressed Mood. *British Journal Of Psychiatry*, *164*, 288-292. https://doi.org/10.1192/bjp.164.3.288

Haver, A., Akerjordet, K., Caputi, P., Furunes, T., & Magee, C. (2015). Measuring mental well-being: a validation of the short Warwick–Edinburgh mental well-being scale in Norwegian and Swedish. *Scandinavian journal of public health*, *43*, 721-727. https://doi.org/10.1177/1403494815588862

Hooper, R. (2014). *Justifying sample size for a feasibility study.* National Institute of Health Research, Research Design Service document. Retrieved from: https://www.rds-london.nihr.ac.uk/RDSLondon/media/RDSContent/files/PDFs/Justifying-Sample-Size-for-a-Feasibility-Study_1.pdf

Hunziker U.A., & Barr R.G. (1986) Increased carrying reduces infant crying: A randomized controlled trial. *Paediatrics*, *77*, 641-648.

Jackson, A. P. (2000). Maternal Self-Efficacy and Children's Influence on Stress and Parenting among Single Black Mothers in Poverty. *Journal of Family Issues*, *21*, 3-

16. https://doi.org/10.1177/019251300021001001

Jones, J., Cassidy, J., & Shaver, P. R. (2015). Adult attachment styles and parenting. In J. Simpson & S. Rholes (Eds.), *New directions in adult attachment research*. New York: Guilford.

Kingston, D., Kehler, H., Austin, M., Mughal, M., Wajid, A., & Vermeyden, L. et al. (2018). Trajectories of maternal depressive symptoms during pregnancy and the first 12 months postpartum and child externalizing and internalizing behavior at three years. *PLOS ONE*, *13*, e0195365. https://doi.org/10.1371/journal.pone.0195365

Knowles, R. (2016). *Why Babywearing Matters* (2nd ed.). London: Pinter and Martin.

Kochanska, G., Coy, K. C., Tjebkes, T. L., & Husarek, S. J. (1998). Individual differences in emotionality in infancy. *Child development*, *69*, 375-390. https://doi.org/10.1111/j.1467-8624.1998.tb06196.x

Kroenke, K., Spitzer, R. L., & Williams, J. B. (2001). The PHQ-9: validity of a brief depression severity measure. *Journal of general internal medicine*, *16*, 606-613. https://doi.org/10.1046/j.1525-1497.2001.016009606.x

Lafontaine, M.-F., Brassard, A., Lussier, Y., Valois, P., Shaver, P. R., & Johnson, S. M. (2015). Selecting the Best Items for a Short-Form of the Experiences in Close Relationships Questionnaire. *European Journal of Psychological Assessment*. Advance online publication. http://dx.doi.org/10.1027/1015-5759/a000243

Le, M., Tran, T., Holton, S., Nguyen, H., Wolfe, R., & Fisher, J. (2017). Reliability, convergent validity and factor structure of the DASS-21 in a sample of Vietnamese adolescents. *PLOS ONE*, *12*, e0180557. https://doi.org/10.1371/journal.pone.0180557

Leahy-Warren, P., McCarthy, G., & Corcoran, P. (2012). First-time mothers: social support, maternal parental self-efficacy and postnatal depression. *Journal of clinical nursing*, *21*, 388-397. https://doi.org/10.1111/j.1365-2702.2011.03701.x

Lovibond, P. F., & Lovibond, S. H. (1995). The structure of negative emotional states: Comparison of the Depression Anxiety Stress Scales (DASS) with the Beck Depression and Anxiety Inventories. *Behaviour research and therapy*, *33*, 335-343. https://doi.org/10.1016/0005-7967(94)00075-U

Maheswaran, H., Weich, S., Powell, J., & Stewart-Brown, S. (2012). Evaluating the responsiveness of the Warwick Edinburgh Mental Well-Being Scale (WEMWBS): Group and individual level analysis. *Health And Quality Of Life Outcomes*, *10*, 156. https://doi.org/10.1186/1477-7525-10-156

McGhee, C. 10 of the best baby slings. Retrieved from http://www.madeformums.com/reviews-and-shopping/10-of-the-best-baby-slings/38008.html

McKellar L, Steen M, Lorensuhewa N. (2017) Capture my mood: a feasibility study to develop a visual scale for women to self-monitor their mental wellbeing following birth. *Evidence Based Midwifery, 15*, 54-59

Milgrom, J., & McCloud, P. (1996). Parenting stress and postnatal depression. *Stress Medicine*, *12*(3), 177-186. https://doi.org/10.1002/(sici)1099-1700(199607)12:3<177::aid-smi699>3.3.co;2-n

Miller, R. L., Pallant, J. F., & Negri, L. M. (2006). Anxiety and stress in the postpartum: is there more to postnatal distress than depression?. *BMC psychiatry*, *6*, 12. https://doi.org/10.1186/1471-244X-6-12

Millings, A., Walsh, J., Hepper, E., & O'Brien, M. (2013). Good partner, good parent: Responsiveness mediates the link between romantic attachment and parenting style. *Personality and Social Psychology Bulletin*, *39*, 170-180. https://doi.org/10.1177/0146167212468333

Moher, D., Schulz, K. F., & Altman, D. G. (2001). The CONSORT statement: revised recommendations for improving the quality of reports of parallel group randomized trials. *BMC medical research methodology*, *1*, 2. https://doi.org/10.1186/1471-2288-1-2

Moraes, G. P. D. A., Lorenzo, L., Pontes, G. A. R., Montenegro, M. C., & Cantilino, A. (2017). Screening and diagnosing postpartum depression: when and how?. *Trends in psychiatry and psychotherapy*, *39*, 54-61. http://doi.org/10.1590/2237-6089-2016-0034.

Mustaffa, S.M., Marappan, D., Abu, S.M., Khan, A. & Ahmad, R. (2014). Social Support during Pre-Natal and Post-Natal Stage: Influence on Maternal Depression and Mental WellBeing. *Procedia-Social and Behavioral Sciences, 143,* 417-422. https://doi.org/ 10.1016/j.sbspro.2014.07.506

National Institute for Health and Care and Excellence (2009). *Depression in adults: recognition and management* (CG90). Retrieved from https://www.nice.org.uk/guidance/cg90

National Institute for Health and Care and Excellence (NICE)(2014). *Antenatal and postnatal mental health: clinical management and service guidance* (CG192). Retrieved from https://www.nice.org.uk/guidance/cg192

NCT: The UK's largest charity for parents. Retrieved from https://www.nct.org.uk/

Ng, F., Trauer, T., Dodd, S., Callaly, T., Campbell, S., & Berk, M. (2007). The validity of the 21-item version of the Depression Anxiety Stress Scales as a routine clinical outcome measure. *Acta Neuropsychiatrica*, *19*, 304-310. https://doi.org/10.1111/j.1601-5215.2007.00217.x

Nielsen, D., Videbech, P., Hedegaard, M., Dalby, J., & Secher, N. J. (2000). Postpartum depression: identification of women at risk. *BJOG: An International Journal of Obstetrics & Gynaecology*, *107*, 1210-1217. https://doi.org/10.1111/j.1471-0528.2000.tb11609.x

Novotney, J., & Maurer, D. (2017). Is the Edinburgh Postnatal Depression Scale an effective way to screen for postpartum depression?. *Evidence-Based Practice*, *7*(20), E7-E8. https://doi.org/ 10.1097/01.EBP.0000541776.26600.35

O'Cathain, A., Hoddinott, P., Lewin, S., Thomas, K.J., Young, B., Adamson, J., Jansen, Y. J. F. M., Mills, N., Moore, G., & Donovan, J. L. (2015). Maximising the impact of qualitative research

in feasibility studies for randomised controlled trials: guidance for researchers. *Pilot and Feasibility Studies, 1,* 32. https://doi.org/10.1186/s40814-015-0026-y

Ohan, J. L., Leung, D. W., & Johnston, C. (2000). The Parenting Sense of Competence Scale: Evidence of a stable factor structure and validity. *Canadian Journal of Behavioural Science/Revue Canadienne des Sciences du Comportement, 32*, 251-261. https://doi.org/10.1007/s10826-015-0149-z

Onozawa, K., Glover, V., Adams, D., Modi, N., & Kumar, R. (2001). Infant massage improves mother–infant interaction for mothers with postnatal depression. *Journal Of Affective Disorders*, *63*, 201-207. https://doi.org/10.1016/s0165-0327(00)00198-1

Parade, S. H., & Leerkes, E. M. (2008). The reliability and validity of the Infant Behavior Questionnaire-Revised. *Infant Behavior and Development*, *31*, 637-646. https://doi.org/10.1016/j.infbeh.2008.07.009

Peterson, E. R., Mohal, J., Waldie, K. E., Reese, E., Atatoa Carr, P. E., Grant, C. C., & Morton, S. M. (2017). A cross-cultural analysis of the Infant Behavior Questionnaire Very Short Form: An item response theory analysis of infant temperament in New Zealand. *Journal of personality assessment*, *99*, 574-584. https://doi.org/10.1080/00223891.2017.1288128

Pisacane, A., Continisio, P., Filosa, C., Tagliamonte, V., & Continisio, G. I. (2012). Use of baby carriers to increase breastfeeding duration among term infants: the effects of an educational intervention in Italy. *Acta Paediatrica*, *101*, e434-e438. https://doi.org/10.1111/j.1651-2227.2012.02758.x

Pope, S., & Pope, S. (2000). *Postnatal depression: A systematic review of published scientific literature to 1999: An information paper*. Australia: National Health and Medical Research Council.

Posado, G., Waters, E., Crowell, J. A., & Lay, K. L. (1995). Is it easier to use a secure mother as a secure base? Attachment Q-sort correlates of the adult attachment interview. *Monographs of the Society for Research in Child Development*, *60*, 133-145. https://doi.org/10.1111/j.1540-5834.1995.tb00208.x

Puckering, C., McIntosh, E., Hickey, A., & Longford, J. (2010). Mellow Babies: a group intervention for infants and mothers experiencing postnatal depression. *Counselling Psychology Review*, *25*, 28-40. Retrieved from https://www.mellowparenting.org/wp-content/uploads/2016/03/Mellow-Babies-A-group-intervention-for-infants-and-mothers-PND-Puckering-et-al-2010.pdf

Putnam, S. P., Helbig, A. L., Gartstein, M. A., Rothbart, M. K. & Leerkes, E. (2014). Development and Assessment of Short and Very Short Forms of the Infant Behavior Questionnaire-Revised. *Journal of Personality Assessment, 96,* 445-458. https://doi.org/10.1080/00223891.2013.841171

Rholes, W. S., Simpson, J. A., & Friedman, M. (2006). Avoidant attachment and the experience of parenting. *Personality and social psychology bulletin*, *32*, 275-285. https://doi.org/10.1177/0146167205280910

Røhder, K., George, C., Brennan, J., Nayberg, E., Trier, C., & Harder, S. (2018). The cross-cultural

validity of the Caregiving Experiences Questionnaire (CEQ) among Danish mothers with preschool children. *European Journal Of Developmental Psychology*, 1-13. https://doi.org/10.1080/17405629.2017.1419951

Rothbart, M. K. (1981). Measurement of temperament in infancy. *Child Development, 52,* 569-578. https://doi.org/10.2307/1129176

Russell, D., & Cutrona, C. E. (1984, August). The provisions of social relationships and adaptation to stress. Paper presented at the annual meeting of the American Psychological Association, Anaheim, CA.

Sharp, H., Pickles, A., Meaney, M., Marshall, K., Tibu., & Hill, J. (2012). Frequency of infant stroking reported by mothers moderates the effect of prenatal depression on infant behavioural and physiological outcomes. *PLoS ONE, 7,* e45446. http://doi.org/10.1371/jounral.pone.0045446.

Simpson, J. A., Rholes, W. S., & Nelligan, J. S. (1992). Support seeking and support giving within couples in an anxiety-provoking situation: The role of attachment styles. *Journal of personality and social psychology*, *62*, 434. http://dx.doi.org/10.1037/0022-3514.62.3.434

Stewart-Brown, S. (2013). The Warwick-Edinburgh Mental Well-being Scale (WEMWBS): performance in different cultural and geographical groups. In *Mental well-being* (pp. 133-150). Springer, Dordrecht. https://doi.org/10.1007/978-94-007-5195-8_7

Stewart-Brown, S., Tennant, A., Tennant, R., Platt, S., Parkinson, J., & Weich, S. (2009). Internal construct validity of the Warwick-Edinburgh Mental Well-being Scale (WEMWBS): a Rasch

analysis using data from the Scottish Health Education Population Survey. *Health and Quality of Life Outcomes*, *7*, 15. https://doi.org/10.1186/1477-7525-7-15

Tennant, R., Hiller, L., Fishwick, R., Platt, S., Joseph, S., & Weich, S. et al. (2007). The Warwick-Edinburgh Mental Well-being Scale (WEMWBS): development and UK validation. *Health and Quality of Life Outcomes*, *5*, 63. https://doi.org/10.1186/1477-7525-5-63

Thoits, P. A. (1985). Social support and psychological well-being: Theoretical possibilities. In *Social support: Theory, research and applications* (pp. 51-72). Springer, Dordrecht.

Uvnäs-Moberg, K. U., & Prime, D. K. (2013). Oxytocin effects in mothers and infants during breastfeeding. *Infant*, *9*(6), 201-206.

Warwick-Edinburgh Mental Wellbeing Scale (WEMWBS). Retrieved from https://warwick.ac.uk/fac/sci/med/research/platform/wemwbs/

Welcome to the Sheffield Sling Surgery and Library! - Sheffield Sling Surgery. Retrieved from https://www.sheffieldslingsurgery.co.uk/

Williams, M. (2016). *Guide to feasibility and pilot studies: a guide for NIHR Research Design Service advisors.* London, UK: National Institute for Health Research, Research Design Service.

Winberg, J. (2005). Mother and newborn baby: Mutual regulation of physiology and behaviour, a selective review. *Developmental Psychobiology*, *47*, 217-229. https://doi.org/10.1002/dev.20094

Appendix A Recruitment Materials

A.1: Recruitment Poster.

[Logos removed]

Participants Needed - Free Sling Hire!

We are seeking volunteers for a maternal mental health and wellbeing research project.

[Images removed]

We are looking for women who are due to give birth between March 2019 and July 2019, and who have not used a baby sling before.

We want to see if using a sling might influence your mood, your general wellbeing, or your parenting experiences.

If you agree to participate:

- When your child is born, we would ask you to complete a set of online questionnaires 3 times over the course of 12 weeks.
- We would need you to travel to the Sheffield Sling Surgery for a one-off session where you will learn how to safely use a sling and be given a sling to hire for free for the duration of the study.
- You would be randomly assigned to one of two groups. For one group you will be asked to attend this session when your child is between 0 and 6 weeks old. If you are in the other group you will be asked to attend when your child is between 12 and 18 weeks old.

If you or anyone you know might be interested in participating, to learn more about it please email Helen Wigglesworth (Trainee Clinical Psychologist and Principal Investigator) at:

hmwigglesworth1@sheffield.ac.uk

Contacting us in no way commits you to participate. And if you do agree to take part in this study, you can stop participating at any time.

This study has been approved by the University of Sheffield's Ethics Committee, reference 024147.

Appendix B Items for All Time Points

<u>Sling Use Measure</u>

Sling Use and Access to Sling Surgery Services

Over the past 6 weeks, how often would you say that you have used your sling?

- ☐ Not at all
- ☐ Once or twice
- ☐ A few times
- ☐ About once a day
- ☐ About twice or three times a day
- ☐ More than three times a day

Over the past 6 weeks, how often would you say that you have used a pram or buggy (or similar)?

- ☐ Not at all
- ☐ Once or twice
- ☐ A few times
- ☐ About once a day
- ☐ About twice or three times a day
- ☐ More than three times a day

Over the past 6 weeks, how often would you say that you have accessed the sling surgery services (either online, in person, or other)?

- ☐ Not at all
- ☐ About once a week
- ☐ About twice a week
- ☐ Generally more than three times a week
- ☐ Daily

You will be now be asked the same questions again, but about your partner:

Over the past 6 weeks, how often would you say that your partner has used the sling?

- ☐ Not at all
- ☐ Once or twice
- ☐ A few times
- ☐ About once a day
- ☐ About twice or three times a day
- ☐ More than three times a day
- ☐ N/A

Over the past 6 weeks, how often would you say that your partner has used a pram or buggy (or similar)?

- ☐ Not at all

- ☐ Once or twice
- ☐ A few times
- ☐ About once a day
- ☐ About twice or three times a day
- ☐ More than three times a day
- ☐ N/A

Over the past 6 weeks, how often would you say that your partner has accessed the sling surgery services (either online, in person, or other)?

- ☐ Not at all
- ☐ About once a week
- ☐ About twice a week
- ☐ Generally more than three times a week
- ☐ Daily
- ☐ N/A

Over the past six weeks, have you needed to swap your sling for a different one, and if so, how many times have you swapped slings?

- ☐ I have not swapped slings.
- ☐ Yes, I have swapped slings once.
- ☐ Yes, I have swapped slings twice.
- ☐ Yes, I have swapped slings three times.
- ☐ Yes, I have swapped slings more than three times.

<u>Mental Health (Access to Support) Items</u>

Overall, how would you rate your mental health currently?

- ☐ Good
- ☐ Somewhat Good
- ☐ Average
- ☐ Somewhat Poor
- ☐ Poor

Are you currently accessing support for any mental health difficulties? (e.g. medication, therapy, support groups etc.).

- ☐ Yes
- ☐ No

If yes, would you mind saying what support you are accessing?

Appendix C Edinburgh Post-natal Depression Scale

(EPDS; Cox, Holden & Sagovsky, 1987)

As you are pregnant or have recently had a baby, we would like to know how you are feeling. Please check the answer that comes closest to how you have felt **IN THE PAST 7 DAYS**, not just how you feel today.

Here is an example, already completed.

I have felt happy:
- ☐ Yes, all the time
- ☒ Yes, most of the time
- ☐ No, not very often
- ☐ No, not at all

This would mean: "I have felt happy most of the time" during the past week. Please complete the other questions in the same way.

In the past 7 days:

1. I have been able to laugh and see the funny side of things
   - ☐ As much as I always could
   - ☐ Not quite so much now
   - ☐ Definitely not so much now
   - ☐ Not at all

2. I have looked forward with enjoyment to things
   - ☐ As much as I ever did
   - ☐ Rather less than I used to
   - ☐ Definitely less than I used to
   - ☐ Hardly at all

*3. I have blamed myself unnecessarily when things went wrong
   - ☐ Yes, most of the time
   - ☐ Yes, some of the time
   - ☐ Not very often
   - ☐ No, never

4. I have been anxious or worried for no good reason
   - ☐ No, not at all
   - ☐ Hardly ever
   - ☐ Yes, sometimes
   - ☐ Yes, very often

*5 I have felt scared or panicky for no very good reason
   - ☐ Yes, quite a lot
   - ☐ Yes, sometimes
   - ☐ No, not much
   - ☐ No, not at all

*6. Things have been getting on top of me
   - ☐ Yes, most of the time I haven't been able to cope at all
   - ☐ Yes, sometimes I haven't been coping as well as usual
   - ☐ No, most of the time I have coped quite well
   - ☐ No, I have been coping as well as ever

*7 I have been so unhappy that I have had difficulty sleeping
   - ☐ Yes, most of the time
   - ☐ Yes, sometimes
   - ☐ Not very often
   - ☐ No, not at all

*8 I have felt sad or miserable
   - ☐ Yes, most of the time
   - ☐ Yes, quite often
   - ☐ Not very often
   - ☐ No, not at all

*9 I have been so unhappy that I have been crying
   - ☐ Yes, most of the time
   - ☐ Yes, quite often
   - ☐ Only occasionally
   - ☐ No, never

*10 The thought of harming myself has occurred to me
   - ☐ Yes, quite often
   - ☐ Sometimes
   - ☐ Hardly ever
   - ☐ Never

Appendix D Ten-point Supplement to EPDS Manual (Cox, Holden & Henshaw, 2014)

**When using the EPDS for clinical or research purposes, give very careful consideration to the following ten points:**
1. Be careful to check the validity of the scale for the population of mothers completing the EPDS.
2. Establish its sensitivity, specificity, positive predictive value and optimal cut off points for the purpose of your clinical or research work.
3. Remember that the EPDS is NOT diagnostic of mental disorder. A 'high score' indicates that depressive symptoms are present—but not their duration or intensity. The EPDS is not a test for PND or for an anxiety disorder.
4. When using the EPDS in other languages, make sure that the back translation is satisfactory and that there is also evidence of satisfactory Face, Semantic, Conceptual and Technical validity.
5. Remember that the EPDS is NOT a check list of common symptoms of perinatal depression. It deliberately omitted somatic symptoms and items concerned with the mother-infant relationship.
6. Remember that the EPDS was validated, piloted and evaluated in Edinburgh by a clinically informed research team to assist with the detection of postnatal and antenatal depression in community clinics.
7. When using the EPDS as an aid to assessment, or in universal or targeted screening, remember that its administration must be supervised by a trained health professional with access to mental health services.
8. When used to assess a mother in the community, the practitioner should discuss the responses with her, listen to her story, ascertain whether clinical depression or another mental disorder is present—and consider referral and/or further listening visits.
9. The risk of developing a postnatal psychosis should always be assessed. The EPDS does NOT screen for bipolar disorder.
10. The EPDS was developed in Edinburgh by John Cox, Jenifer Holden and Ruth Sagovsky over thirty years ago. Please read the recent EPDS Manual (Cox et al. 2014) so that the scale continues to be used optimally in research and clinical work.

Appendix E EPDS: Postnatal Depression Threshold Letter

The EPDS is not a diagnostic tool, but it is a screening tool which is designed to identify women who may benefit from further assessment or follow-up care (Cox, Holden & Sagovsky, 1987).

According to the EPDS instructions, a score of 13 or higher on this measure indicates a high likelihood of depression. As such, it is recommended that the person access primary care services for further assessment.

This letter will be sent to women who achieve a score of 13 or more when completing the EPDS at any time point.

Dear **[insert name]**,

You recently completed a set of questionnaires as part of your participation in the Sling Provision and Maternal Wellbeing study.

On one of the questionnaires, the Edinburgh Postnatal Depression Scale, the score you have suggests that you may be feeling low, tired or worried, following the birth of your child. It may be that you are suffering from postnatal depression, which is very common among families and underdiagnosed. There is help available if you are feeling this way. We recommend that you contact your GP, midwife or health visitor, to talk about these experiences, and seek further support.

Whatever you decide to do, whether you contact your GP, midwife or health visitor, or not, this will not impact on your participation in this study.

If you feel that at this point you would like to withdraw from the study, please feel free to do so, but please let us know by emailing the address below.

Hmwigglesworth1@sheffield.ac.uk

Thank you and best wishes,

Helen Wigglesworth and Abi Millings.

Sling Use and Maternal Wellbeing Project,

University of Sheffield.

Appendix F Depression Anxiety and Stress Scales-21 (DASS-21; Lovibond & Lovibond, 1995).

DASS21

Name: *Date:*

Please read each statement and circle a number 0, 1, 2 or 3 which indicates how much the statement applied to you *over the past week*. There are no right or wrong answers. Do not spend too much time on any statement.

The rating scale is as follows:

0 Did not apply to me at all
1 Applied to me to some degree, or some of the time
2 Applied to me to a considerable degree, or a good part of time
3 Applied to me very much, or most of the time

1	I found it hard to wind down	0	1	2	3
2	I was aware of dryness of my mouth	0	1	2	3
3	I couldn't seem to experience any positive feeling at all	0	1	2	3
4	I experienced breathing difficulty (eg, excessively rapid breathing, breathlessness in the absence of physical exertion)	0	1	2	3
5	I found it difficult to work up the initiative to do things	0	1	2	3
6	I tended to over-react to situations	0	1	2	3
7	I experienced trembling (eg, in the hands)	0	1	2	3
8	I felt that I was using a lot of nervous energy	0	1	2	3
9	I was worried about situations in which I might panic and make a fool of myself	0	1	2	3
10	I felt that I had nothing to look forward to	0	1	2	3
11	I found myself getting agitated	0	1	2	3
12	I found it difficult to relax	0	1	2	3
13	I felt down-hearted and blue	0	1	2	3
14	I was intolerant of anything that kept me from getting on with what I was doing	0	1	2	3
15	I felt I was close to panic	0	1	2	3
16	I was unable to become enthusiastic about anything	0	1	2	3
17	I felt I wasn't worth much as a person	0	1	2	3
18	I felt that I was rather touchy	0	1	2	3
19	I was aware of the action of my heart in the absence of physical exertion (eg, sense of heart rate increase, heart missing a beat)	0	1	2	3
20	I felt scared without any good reason	0	1	2	3
21	I felt that life was meaningless	0	1	2	3

Appendix G Experiences in Close Relationships Scale-12 (ECR-12; Lafontaine et al., 2015)

Experiences in Close Relationship Scale-Short Form (ECR-S)

Instruction: The following statements concern how you feel in romantic relationships. We are interested in how you generally experience relationships, not just in what is happening in a current relationship. Respond to each statement by indicating how much you agree or disagree with it. Mark your answer using the following rating scale:

1	2	3	4	5	6	7
Strongly Disagree	Disagree	Slightly Disagree	Neutral	Slightly Agree	Agree	Strongly Agree

1. It helps to turn to my romantic partner in times of need.

2. I need a lot of reassurance that I am loved by my partner.

3. I want to get close to my partner, but I keep pulling back.

4. I find that my partner(s) don't want to get as close as I would like.

5. I turn to my partner for many things, including comfort and reassurance.

6. My desire to be very close sometimes scares people away.

7. I try to avoid getting too close to my partner.

8. I do not often worry about being abandoned.

9. I usually discuss my problems and concerns with my partner.

10. I get frustrated if romantic partners are not available when I need them.

11. I am nervous when partners get too close to me.

12. I worry that romantic partners won't care about me as much as I care about them.

Appendix H Warwick Edinburgh Mental Wellbeing Scale (WEMWBS; Tennant et al., 2007)

Below are some statements about feelings and thoughts.

Please tick (√) the box that best describes your experience of each over the **last 2 weeks.**

STATEMENTS	None of the Time	Rarely	Some of the time	Often	All of the time
I've been feeling optimistic about the Future	1	2	3	4	5
I've been feeling useful	1	2	3	4	5
I've been feeling relaxed	1	2	3	4	5
I've been feeling interested in other People	1	2	3	4	5
I've had energy to spare	1	2	3	4	5
I've been dealing with problems well	1	2	3	4	5
I've been thinking clearly	1	2	3	4	5
I've been feeling good about myself	1	2	3	4	5
I've been feeling close to other People	1	2	3	4	5
I've been feeling confident	1	2	3	4	5
I've been able to make up my own mind about things	1	2	3	4	5
I've been feeling loved	1	2	3	4	5
I've been interested in new things	1	2	3	4	5
I've been feeling cheerful	1	2	3	4	5

© WEMWBS

Appendix I Short Warwick Edinburgh Mental Well-Being Scale
(SWEMWBS)

Below are some statements about feelings and thoughts.

Please tick the box that best describes your experience of each over the last 2 weeks

STATEMENTS	None of the time	Rarely	Some of the time	Often	All of the time
I've been feeling optimistic about the future	1	2	3	4	5
I've been feeling useful	1	2	3	4	5
I've been feeling relaxed	1	2	3	4	5
I've been dealing with problems well	1	2	3	4	5
I've been thinking clearly	1	2	3	4	5
I've been feeling close to other people	1	2	3	4	5
I've been able to make up my own mind about things	1	2	3	4	5

Appendix J Parenting Sense of Competency Scale (PSCS; Gibaud-Wallston & Wandersman, 1978)

Please rate the extent to which you agree or disagree with each of the following statements.

Strongly Disagree	Somewhat Disagree	Disagree	Agree	Somewhat Agree	Strongly Agree
1	2	3	4	5	6

1. The problems of taking care of a child are easy to solve once you know how your actions affect your child, an understanding I have acquired.	1 2 3 4 5 6
2. Even though being a parent could be rewarding, I am frustrated now while my child is at his / her present age.	1 2 3 4 5 6
3. I go to bed the same way I wake up in the morning, feeling I have not accomplished a whole lot.	1 2 3 4 5 6
4. I do not know why it is, but sometimes when I'm supposed to be in control, I feel more like the one being manipulated.	1 2 3 4 5 6
5. My mother was better prepared to be a good mother than I am.	1 2 3 4 5 6
6. I would make a fine model for a new mother to follow in order to learn what she would need to know in order to be a good parent.	1 2 3 4 5 6
7. Being a parent is manageable, and any problems are easily solved.	1 2 3 4 5 6
8. A difficult problem in being a parent is not knowing whether you're doing a good job or a bad one.	1 2 3 4 5 6
9. Sometimes I feel like I'm not getting anything done.	1 2 3 4 5
10. I meet by own personal expectations for expertise in caring for my child.	1 2 3 4 5 6
11. If anyone can find the answer to what is troubling my child, I am the one.	1 2 3 4 5 6
12. My talents and interests are in other areas, not being a parent.	1 2 3 4 5 6
13. Considering how long I've been a mother, I feel thoroughly familiar with this role.	1 2 3 4 5 6
14. If being a mother of a child were only more interesting, I would be motivated to do a better job as a parent.	1 2 3 4 5 6
15. I honestly believe I have all the skills necessary to be a good mother to my child.	1 2 3 4 5 6
16. Being a parent makes me tense and anxious.	1 2 3 4 5 6

Appendix K Items from the Caregiving Experiences Questionnaire

(CEQ; Brennan, George & Solomon, 2013)

E - Enjoyment, H - Heightened, Hs - Helplessness, RR- Role Reversal

I feel that my child is worth all the love and attention I give him or her. *(E)*
I am scared of my child. (My child scares me.) *(Hs)*
I enjoy being with my child when s/he is learning. *(E)*
I am delighted when I see my child's face. *(E)*
My child is amazing. I am in awe of him or her. *(RR)*
My child is my top priority. *(E)*
My child is a real part of me. I can't imagine what it would be like to live without him or her. *(RR)*
My child deserves my love and attention, especially when he or she is not feeling well. *(E)*
I'm so lucky to have my child. S/he is a miracle—the best gift I ever had. Being a mother is wondrous. *(RR)*
It frightens me when my child is angry and I plead with him or her to stop. *(Hs)*
When s/he gets up in the morning, just to see the smile on his or her face—I know that s/he loves me, s/he trusts me. *(E)*
I feel helpless as a mother. *(Hs)*
My child pushes me away or ignores me a lot. *(DC)*
I feel like I'm walking on eggshells when I am with my child. *(Hs)*
My child is so happy when s/he can do something on his own. S/he has a huge smile on his or her face like "I'm so proud, mom, I'm so proud of myself." *(E)*
My child is really gifted. *(RR)*
My child is just as happy and excited to see me as I am to see him or her after we have been away from each other for a while. *(E)*
I'm not inclined to give my child many hugs and kisses when s/he gets hurt. S/he's going to get hurt the rest of his or her life, so s/he might as well get used to it. *(DC)*
I am lonely when my child and I are separated. *(H)*
I think about my child constantly when we've been separated for a while. I really don't know what to do without him or her for very long. *(H)*
Being away from my child makes me feel guilty. *(H)*
When I'm away from my child, I've got to do something else so s/he isn't on my mind. *(H)*
I get sad when I realize that my child won't stay a baby forever. *(H)*
Sometimes I just lose it and scream at him or her or punish too harshly. *(Hs)*
When I'm angry at my child, I have to leave the room so I don't explode. *(Hs)*
There are a lot of times when I cannot control or restrain my child. *(Hs)*
I get out of control and there's nothing I can do about it. *(Hs)*
Sometimes I think my child would be better off if I weren't there and somebody else could do better. *(Hs)*
My child can get so demanding. It's particularly annoying when s/he makes demands after I've just done something special for him or her. *(DC)*
My child can get wild and out of control. *(Hs)*
Life is chaotic and my child makes me feel out of control. *(Hs)*
My child can get so wound up and out of control and I don't want to take him or her anywhere. *(Hs)*
I get overwhelmed because my child always needs my help and cannot handle problems on his or her own. *(Hs)*
Sometimes being a parent seems like a battle and if my child won't cooperate, one of us must give in. *(Hs)*
Sometimes I say, "I can't do this right now." I need time to relax. *(DC)*
My child and I are really close. I can just sit there and tell him or her if I had a bad day and s/he understands. *(RR)*
My child and I are so close we can almost tell each other's feelings. We're really tuned into each other. *(RR)*
My child cheers me up if I am sad or angry. S/he makes me smile and feel better. *(RR)*
My child goes out of his way to be sensitive and tuned in to me and others. *(RR)*

Appendix L Infant Behaviour Questionnaire-Revised Very Short Form

(IBQ-R VSF; Putnam, Helbig, Gartstein, Rothbart, & Leerkes, 2014)

INSTRUCTIONS:
Please read carefully before starting:

As you read each description of the baby's behavior below, please indicate how often the baby did this during the LAST WEEK (the past seven days) by circling one of the numbers in the left column. These numbers indicate how often you observed the behavior described during the last week.

1	2	3	4	5	6	7	NA
**Never**	**Very Rarely**	**Less Than Half the Time**	**About Half the Time**	**More Than Half the Time**	**Almost Always**	**Always**	**Does Not Apply**

The "Does Not Apply" (X) column is used when you did not see the baby in the situation described during the last week. For example, if the situation mentions the baby having to wait for food or liquids and there was no time during the last week when the baby had to wait, circle the (X) column. "Does Not Apply" is different from "Never" (1). "Never" is used when you saw the baby in the situation but the baby never engaged in the behavior listed during the last week. For example, if the baby did have to wait for food or liquids at least once but never cried loudly while waiting, circle the (1) column.

Please be sure to circle a number for every item.

1. When being dressed or undressed during the last week, how often did the baby squirm and/or try to roll away?

1 2 3 4 5 6 7 NA

2. When tossed around playfully how often did the baby laugh?

1 2 3 4 5 6 7 NA

3. When tired, how often did your baby show distress?

1 2 3 4 5 6 7 NA

4. When introduced to an unfamiliar adult, how often did the baby cling to a parent?

1 2 3 4 5 6 7 NA

5. How often during the last week did the baby enjoy being read to?

1 2 3 4 5 6 7 NA

6. How often during the last week did the baby play with one toy or object for 5-10 minutes?

1 2 3 4 5 6 7 NA

7. How often during the week did your baby move quickly toward new objects?

1 2 3 4 5 6 7 NA

8. When put into the bath water, how often did the baby laugh?

1 2 3 4 5 6 7 NA

9. When it was time for bed or a nap and your baby did not want to go, how often did s/he whimper or sob?

1 2 3 4 5 6 7 NA

10. After sleeping, how often did the baby cry if someone doesn't come within a few minutes?

1 2 3 4 5 6 7 NA

11. In the last week, while being fed in your lap, how often did the baby seem eager to get away as soon as the feeding was over?

1 2 3 4 5 6 7 NA

12. When singing or talking to your baby, how often did s/he soothe immediately?

1 2 3 4 5 6 7 NA

13. When placed on his/her back, how often did the baby squirm and/or turn body?

1 2 3 4 5 6 7 NA

14. During a peekaboo game, how often did the baby laugh?

1 2 3 4 5 6 7 NA

15. How often does the infant look up from playing when the telephone rings?

1 2 3 4 5 6 7 NA

16. How often did the baby seem angry (crying and fussing) when you left her/him in the crib?

1 2 3 4 5 6 7 NA

17. How often during the last week did the baby startle at a sudden change in body position (e.g., when moved suddenly)?

1 2 3 4 5 6 7 NA

18. How often during the last week did the baby enjoy hearing the sound of words, as in nursery rhymes?

1 2 3 4 5 6 7 NA

19. How often during the last week did the baby look at pictures in books and/or magazines for 5 minutes or longer at a time?

1 2 3 4 5 6 7 NA

20. When visiting a new place, how often did your baby get excited about exploring new surroundings?

1 2 3 4 5 6 7 NA

21. How often during the last week did the baby smile or laugh when given a toy?

1 2 3 4 5 6 7 NA

22. At the end of an exciting day, how often did your baby become tearful?

1 2 3 4 5 6 7 NA

23. How often during the last week did the baby protest being placed in a confining place (infant seat, play pen, car seat, etc.)?

1 2 3 4 5 6 7 NA

24. When being held, in the last week, did your baby seem to enjoy him/herself?

1 2 3 4 5 6 7 NA

25. When showing the baby something to look at, how often did s/he soothe immediately?

1 2 3 4 5 6 7 NA

26. When hair was washed, how often did the baby vocalize?

1 2 3 4 5 6 7 NA

27. How often did your baby notice the sound of an airplane passing overhead?

1 2 3 4 5 6 7 NA

28. When introduced to an unfamiliar adult, how often did the baby refuse to go to the unfamiliar person?

1 2 3 4 5 6 7 NA

29. When you were busy with another activity, and your baby was not able to get your attention, how often did s/he cry?

1 2 3 4 5 6 7 NA

30. How often during the last week did the baby enjoy gentle rhythmic activities, such as rocking or swaying?

1 2 3 4 5 6 7 NA

31. How often during the last week did the baby stare at a mobile, crib bumper or picture for 5 minutes or longer?

1 2 3 4 5 6 7 NA

32. When the baby wanted something, how often did s/he become upset when s/he could not get what s/he wanted?

1 2 3 4 5 6 7 NA

33. When in the presence of several unfamiliar adults, how often did the baby cling to a parent?

1 2 3 4 5 6 7 NA

34. When rocked or hugged, in the last week, did your baby seem to enjoy him/herself?

1 2 3 4 5 6 7 NA

35. When patting or gently rubbing some part of the baby's body, how often did s/he soothe immediately?

1 2 3 4 5 6 7 NA

36. How often did your baby make talking sounds when riding in a car?

1 2 3 4 5 6 7 NA

37. When placed in an infant seat or car seat, how often did the baby squirm and turn body?

1 2 3 4 5 6 7 NA

Appendix M Short Version of the Social Provisions Scale (Russell & Cutrona, 1984)

As Presented in Cutrona & Troutman, 1986.

“Each item was rated on a 7-point scale (from "Not at all true" to "Completely true"). Items included in the short version were:

I can always depend on my family to help me if I really need it.

I have friends who enjoy the same activities I do.

I don't think people at work, school, or in groups I belong to know and value me.

There is a trustworthy person I could turn to for advice if I were having problems.

There is no one who really relies on me for their well-being.”

Appendix N Maternal Stroking Questions as seen in Sharp et al., 2012

The Parent-Infant Caregiving Touch Scale (Subset of Items)

1. How often do you stroke your baby's face?
☐ Never
☐ Rarely
☐ Sometimes
☐ Often
☐ A lot

2. How often do you stroke your baby's back?
☐ Never
☐ Rarely
☐ Sometimes
☐ Often
☐ A lot

3. How often do you stroke your baby's tummy?
☐ Never
☐ Rarely
☐ Sometimes
☐ Often
☐ A lot

4. How often do you stroke your baby's arms and legs?
☐ Never
☐ Rarely
☐ Sometimes
☐ Often
☐ A lot

Appendix O Demographic Questions

To be included on Qualtrics within online battery of measures given at baseline (Time-point 1). The ethnicity question wording is as recommended by the Office of National Statistics:
https://www.ons.gov.uk/methodology/classificationsandstandards/measuringequality/ethnicgroupnationalidentityandreligion

Age:

- ☐ Under 18
- ☐ 18-25
- ☐ 26-35
- ☐ 36-45
- ☐ 46-55
- ☐ Over 55

What is your ethnic group? (These options are recommended by

Choose one option that best describes your ethnic group or background

White

- ☐ English/Welsh/Scottish/Northern Irish/British
- ☐ Irish
- ☐ Gypsy or Irish Traveller
- ☐ Any other White background, please describe:

Mixed/Multiple ethnic groups

- ☐ White and Black Caribbean
- ☐ White and Black African
- ☐ White and Asian
- ☐ Any other Mixed/Multiple ethnic background, please describe:

Asian/Asian British

- ☐ Indian
- ☐ Pakistani
- ☐ Bangladeshi
- ☐ Chinese
- ☐ Any other Asian background, please describe:

Black/ African/Caribbean/Black British

- ☐ African
- ☐ Caribbean

- ☐ Any other Black/African/Caribbean background, please describe:

Other ethnic group

- ☐ Arab
- ☐ Any other ethnic group, please describe:

Is your child your….?

- ☐ Firstborn
- ☐ Second born
- ☐ Third born
- ☐ Fourth born
- ☐ Fifth born +

Would you like to provide any further information?

…………………………………………………………………………………………
……………..………………………………………………………………………………

How do you feed your baby? (We're interested in their milk feeds only, not solids)

- ☐ Formula feed
- ☐ Breastfeed
- ☐ Both formula feed and breastfeed

Have you attended any antenatal sling workshops during this pregnancy or any previous pregnancies?

- ☐ Yes
- ☐ No

Marital Status

- ☐ Single
- ☐ Married
- ☐ Co-habiting
- ☐ In a relationship, but not co-habiting
- ☐ Separated/Divorced
- ☐ Widowed

Overall, how would you rate your mental health currently?

- ☐ Good
- ☐ Somewhat Good
- ☐ Average
- ☐ Somewhat Poor
- ☐ Poor

Is there a history of mental health difficulties in your family?

- ☐ Yes
- ☐ No
- ☐ Don't Know

Have you ever been diagnosed with a mental health difficulty, either recently or in the past?

- ☐ Yes, prior to my pregnancy
- ☐ Yes, during my pregnancy
- ☐ No

If yes, would you mind saying what it is?

Are you currently accessing support for any mental health difficulties? (e.g. medication, therapy, support groups etc.).

- ☐ Yes
- ☐ No

If yes, would you mind saying what support you are accessing?

Your employment status (excluding maternity leave):

- ☐ Employed full-time
- ☐ Employed part-time
- ☐ Unemployed
- ☐ Student
- ☐ Other

Your partner's employment status:

- ☐ N/A
- ☐ Employed full-time
- ☐ Employed part-time
- ☐ Unemployed
- ☐ Student
- ☐ Other

Household Income (Annually):

- ☐ Less than £10,000
- ☐ £10,000-£19,999
- ☐ £20,000-£29,999
- ☐ £30,000-£39,999
- ☐ £40,000-£49,999
- ☐ £50,000-£59,999

- ☐ £60,000 or over.

What is the highest level of education that you have attained?

- ☐ High School
- ☐ Apprenticeship
- ☐ College Qualification (NVQ, BTEC, Diploma etc.)
- ☐ University - Undergraduate (BA, BSc etc.)
- ☐ University - Postgraduate (Masters -MA, MPhil, MSc, etc; Doctoral - PhD, DPhil, Doctorate etc.)
- ☐ Professional or other Vocational Qualification (e.g. nursing, accountancy, teaching).

What is your Postcode?

……………………………

Appendix P Qualitative Questions

(to be included at T3)

We'd like to know how you have found taking part in this study (the way it was organised, the questionnaires we asked you to complete, your contact with the research team), and how you have found the sling loan and associated support (the services provided by Sheffield Sling Surgery & Library). There are separate questions on each below. Please feel free to say as little or as much as you'd like to.

1. Participation in the Study

How have you found this experience of participating in this study?

Is there anything that you particularly enjoyed/liked about this experience?

Is there anything that you would have wanted to be different?

2. Sling Use

Your first visit:

How was the experience of learning to use a sling when you first visited the Sling Library?

Is there anything that you particularly enjoyed/liked about this experience?

Is there anything that you would have wanted to be different?

Using the sling:

How did you get on with the sling after your initial visit?

Is there anything that you particularly enjoyed/liked about using the sling?

Is there anything that you would have wanted to be different?

Subsequent contact/support with the Sling Library:

What have you liked most about any of the sling surgery services you have used?

Are there any aspects of the sling library services you would want to change or improve?".

Appendix Q Participant Information Sheet

DATE AND VERSION

Sling Provision and Maternal Wellbeing Study

Participant Information Sheet

1. Research Project Title:

Evaluating the Impact of Sling Provision and Training upon Maternal Wellbeing and Parenting: A Randomised Feasibility Trial

You are being invited to take part in a research project. Before you decide whether or not to participate, it is important for you to understand why the research is being done and what it will involve.

Please take time to read the following information carefully and discuss it with others if you wish. Please feel free to contact myself or another member of the research team if there is anything that is not clear or if you would like more information. Contact details may be found further down on this information sheet.

Please take time to decide whether or not you wish to take part. Thank you for taking the time to look at this information sheet.

2. What is the purpose of this study?

The months after childbirth are a physically and emotionally challenging time, and parenting a new-born can be hard. New mothers often feel low, tired, isolated, or anxious in this period. We want to see whether baby slings can help new mothers to cope with the challenges that having a new baby brings.

A baby sling is a piece of cloth that supports an infant or other small child from a carer's body. There are many different types of slings (stretchy wrap, ring, pouch, structured carrier etc.).

Sling libraries loan out slings and carriers and offer advice and information on sling use. Sheffield Sling Surgery is one of the largest sling libraries in the UK.

This study aims to work in collaboration with Sheffield Sling Surgery, to explore whether the provision of a sling, and also the provision of training in the safe use of a sling and how to access peer support from other sling users, may have an impact on the mental health, wellbeing or parenting experiences of women who have recently given birth.

Your involvement, which would be as part of our data collection, should be for around 12 weeks.

This research is being undertaken as part of completion of the Principal Investigator's Doctorate in Clinical Psychology.

3. Why am I being invited to take part?

You have been invited to take part in this study because you have not previously used a sling, and you are due to give birth between late February 2019 and late July 2019. You have been sent this information sheet because you have got in touch in response to an advert. Altogether, we would like around 60 mothers to participate in this study.

4. Do I have to take part?

It is up to you to decide whether or not to take part in this study. If you do decide to take part you will be given this information sheet to keep and be asked to complete a consent form. You can still withdraw from the study in the future without any negative consequences. You do not have to give a reason. If you wish to withdraw from the research, please contact Helen Wigglesworth (Principal Investigator; hmwigglesworth1@sheffield.ac.uk) or Dr. Abigail Millings (Project Supervisor; a.millings@sheffield.ac.uk).

5. What will happen to me if I take part? What do I have to do?

If you agree to take part in the study, please complete the following consent form and participant details form. You will be able to keep a copy of this information sheet and consent form.

Once your baby is born, you will be sent a text or an email asking you to complete a series of online questionnaires. We estimate that altogether these questionnaires should take around **DURATION** minutes to complete. Once you have completed these questionnaires, you may be asked to attend a session at the Sheffield Sling Surgery either as soon as possible, or in 12 weeks' time.

When you attend your session at the Sling Surgery (whether immediately or in 12 weeks' time), you will be offered training and support around how to safely use a baby sling. You will be shown two different types of sling and will be able to hire one sling for free for either 12 weeks (if attending this session immediately) or 4 weeks (if attending after 12 weeks). You will be encouraged to use this sling regularly and sent an email containing information including reminders around safe sling use, and access to the local sling using community. You will be asked to return this sling to the Sheffield Sling Surgery after 4 or 12 weeks, but will be able to re-hire it, or hire another, with the usual hire charges now applying after this time.

You will be asked to complete further online questionnaires 6 weeks and 12 weeks after completing your first set of questionnaires. You will be send text or email reminders at each of these time points, asking you to complete these questionnaires and sending you a webpage link to do so.

6. What are the possible disadvantages and risks of taking part?

The only disadvantage anticipated for taking part in this study is the time that it may take for you to complete these online questionnaires. Otherwise it is not anticipated that participating in this study will cause you any disadvantage or discomfort.

The potential physical and/or psychological harm or distress will be the same as any experienced in everyday life.

7. What are the possible benefits of taking part?

Direct benefits of this study include free sling hire where normally a charge would apply. While there may be no other immediate benefits for those participating in this study, it is hoped that this work will help improve our understanding of the impact of sling use upon maternal mental health, wellbeing and parenting, and will inform future studies on this topic.

8. Will my taking part in this project be kept confidential?

Yes. All of the information that we collect about you during the course of the research will be kept strictly confidential and will only be accessible to members of the research team. You will not be able to be identified in any reports or publications.

9. What is the legal basis for processing my personal data?

According to data protection legislation (General Data Protection Regulation; applicable in the UK and EU from 25 May 2018), we are required to inform you that the legal basis we are applying in order to process your personal data is that 'processing is necessary for the performance of a task carried out in the public interest' (Article 6(1)(e)). Further information can be found in the University's Privacy Notice https://www.sheffield.ac.uk/govern/data-protection/privacy/general.

As we will be collecting some data that is defined in the legislation as more sensitive (information about ethnicity and health), we also need to let you know that we are applying the following condition in law: that the use of your data is "necessary for scientific or historical research purposes".

10. What will happen to the data collected, and the results of the research project?

Any data collected from you (by you filling the questionnaires or giving contact details or other information to the Sling Surgery) will generally be anonymised. This data will be stored securely and will only be available to members of the research team including staff from the Sheffield Sling Surgery. Sling Surgery staff will have access to your contact details and sling hire information.

It will not be possible to anonymise your contact details or information about the date which you first completed these online questionnaires, as this information will be needed in order to send you texts or emails reminding you to complete the online questionnaires at the right time,

or to return your sling at the end of your allotted period of free hire. However this information will be stored securely and securely destroyed once it is no longer necessary for completion of the doctoral thesis or publication of the project (see below).

As the study is part of my doctoral course in Clinical Psychology, it will be submitted to the University for marking. It may be that in the future the findings of this study are published in a relevant journal or presented at a conference. A brief report of the findings will be sent to interested participants. Participants will not be identified within any of these publications.

Due to the nature of this research it is very likely that other researchers may find the data collected to be useful in answering future research questions. Thus anonymous data from this study may be made available to other researchers after this current research is completed.

11. Who is organising and funding the research?

The University of Sheffield is organising and funding the research. This is in collaboration with Sheffield Sling Surgery who are providing the sling hire and training services free of charge.

12. Who is the Data Controller?

The University of Sheffield will act as the Data Controller for this study. This means that the University is responsible for looking after your information and using it properly.

13. Who has ethically reviewed the project?

This project has been ethically approved via the University of Sheffield's Ethics Review Procedure, as administered by the Department of Clinical Psychology. The University's Research Ethics Committee monitors the application and delivery of the University's Ethics Review Procedure across the University.

14. What if something goes wrong and I wish to complain about the research?

If you have a concern about any aspect of this study, please do not hesitate to contact either myself or my project supervisor (please see below):

**Principal Investigator:**	**Project Supervisor:**
Helen Wigglesworth, Trainee Clinical Psychologist<br>hmwigglesworth1@sheffield.ac.uk<br>Clinical Psychology Unit,<br>Department of Psychology,<br>University of Sheffield,	Dr Abigail Millings<br>a.millings@sheffield.ac.uk<br>Lecturer in Psychology, Postgraduate Tutor, and PG Careers Contact,<br>Department of Psychology,<br>University of Sheffield,

Floor F, Cathedral Court,<br>1 Vicar Lane,<br>Sheffield, S1 2LT	Floor D, Cathedral Court,<br>1 Vicar Lane,<br>Sheffield,<br>S1 2LT<br>Tel: 01142226525

Should you feel that your complaint has not been handled to your satisfaction, or if you wish to contact a person external to the project, please do not hesitate to contact our Head of Department:

Professor Glenn Waller,

g.waller@sheffield.ac.uk

Head of Psychology Department

Department of Psychology
University of Sheffield
Floor D, Cathedral Court
1 Vicar Lane
Sheffield
S1 2LT

The Head of Department will then be able to escalate the complaint through the appropriate channels.

If your complaint relates to how your personal data has been handled, then further information about raising this type of complaint may be found in the University's Privacy Notice: https://www.sheffield.ac.uk/govern/data-protection/privacy/general.

15. Contact for further information

For further information please do not hesitate to contact a member of the project team (please see above).

You will be given a copy of the information sheet and of your consent form, to keep.

Thank you for taking the time to read this information sheet.

Appendix R Participant Consent Form

Sling Provision and Maternal Wellbeing Study Participant Consent Form

Please tick the appropriate boxes	**Yes**	**No**
**Taking Part in the Project**		
I have read and understood the project information sheet dated DD/MM/YYYY or the project has been fully explained to me. (If you will answer No to this question please do not proceed with this consent form until you are fully aware of what your participation in the project will mean.)	☐	☐
I have been given the opportunity to ask questions about the project.	☐	☐
I understand that taking part in the project may include:		
Completing a number of questionnaires over the course of around 12 weeks.	☐	☐
Attending one session at the Sheffield Sling Surgery, when my infant is either between 0 and 6 weeks old or between 12 and 18 weeks old.	☐	☐
Use of a baby sling.	☐	☐
Being contacted by the Sling Surgery and research project staff via email and/or text.	☐	☐
I agree to take part in the project.	☐	☐
I understand that my taking part is voluntary and that I can withdraw from the study at any time; I do not have to give any reasons for why I no longer want to take part and there will be no adverse consequences if I choose to withdraw.	☐	☐
**How my information will be used during and after the project**		
I understand my personal details such as name, phone number, address and email address etc. will not be revealed to people outside the project.	☐	☐
I understand and agree that my words may be quoted in publications, reports, web pages, and other research outputs. I understand that I will not be named in these outputs unless I specifically request this.	☐	☐
I understand and agree that any data that is collected during the study will only be shared with members of the research team.	☐	☐
I understand and agree that other authorised researchers may use my data in publications, reports, web pages, and other research outputs, only if they agree to preserve the confidentiality of the information as requested in this form.	☐	☐
**So that the information you provide can be used legally by the researchers**		
I agree to assign the copyright I hold in any materials generated as part of this project to The University of Sheffield.	☐	☐

Name of participant [printed] Signature Date

Name of Researcher [printed] Signature Date

For further information, please do not hesitate to contact us:

Principal Investigator:
Helen Wigglesworth, Trainee Clinical Psychologist

hmwigglesworth1@sheffield.ac.uk
Clinical Psychology Unit
Department of Psychology
University of Sheffield
Floor F, Cathedral Court
1 Vicar Lane
Sheffield, S1 2LT

Project Supervisor:
Dr Abigail Millings

a.millings@sheffield.ac.uk
Lecturer in Psychology, Postgraduate Tutor, and PG Careers Contact
Department of Psychology
University of Sheffield
Floor D, Cathedral Court
1 Vicar Lane
Sheffield
S1 2LT

Tel: 01142226525

In the event of a complaint, if you wish to contact a person external to the project, please contact:

Professor Glenn Waller,
g.waller@sheffield.ac.uk
Head of Psychology Department
Department of Psychology
University of Sheffield
Floor D, Cathedral Court
1 Vicar Lane
Sheffield
S1 2LT

This consent form has been approved by the University of Sheffield Research Ethics Committee, reference XXXX.

Appendix S Templates of all Emails/Texts and Qualtrics Pages

S.1 Further Information Email

Please find below the email that will be sent if someone contacts us after seeing a recruitment poster/flyer.

Hello,

Thank you for contacting us about the sling use and maternal wellbeing study.

Please click on the link below for further information about the study and what participating in the study would involve for you. Through this link, please complete the consent form that follows this information, indicating whether you wish to participate in this study.

Insert Qualtrics link

We have attached to this email copies of the study information and the consent form for you to keep.

If you have any questions or concerns about this study and your participation, then please do not hesitate to contact us, either using this email address or the details included in the information sheet and consent form.

Thank you for taking the time to contact us and read this information. We look forward to hearing from you.

Best wishes,

Helen Wigglesworth (Trainee Clinical Psychologist) and Abi Millings (Lecturer in Psychology and Project Supervisor)

University of Sheffield.

S.2 Participant Detail Form to be included on Qualtrics

This is a page that will be set up on Qualtrics. When participants read the information sheet and complete the consent form, they will then be shown this page which will ask for their contact details and preferred method of communication. This page will be skipped if they do not consent to participating in the study.

Thank you for consenting to participate in this study. Please enter your name, due date, and contact details below:

Name:

How did you hear about this study?:

- ☐ From a friend/relative
- ☐ Via. The National Childbirth Trust
- ☐ Via. Forging Families

- ☐ From advertising in a baby care shop
- ☐ From advertising in a library
- ☐ Via a church/church-run group
- ☐ From advertising in a café
- ☐ From advertising in a soft play centre
- ☐ From advertising in a sports/yoga centre
- ☐ From advertising in a private health clinic (e.g. chiropractors, physiotherapists)
- ☐ Via an online group (Mumsnet, facebook, Instagram etc.)
- ☐ Via the Sheffield Sling Surgery
- ☐ Other

  If other, please state here: …………………….

Due date:

Email address:

Telephone number (mobile):

Generally, I would prefer to be contacted by:

- ☐ Email
- ☐ Text
- ☐ Both email and text

Thank you very much for agreeing to participate in this study. You will now receive an email with information about what happens next.

Aside from this email, and contact from the Sling Surgery, we will aim to contact you via your preferred method, as indicated by yourself above.

If you have any questions at this stage, please do not hesitate to contact us on:

Hmwigglesworth1@sheffield.ac.uk

If you did not mean to consent to participate in the study, please contact us on the above email address to let us know, and we will remove your information immediately.

Thank you and best wishes,

Helen Wigglesworth (Trainee Clinical Psychologist) and Abi Millings (Lecturer in Psychology and Project Supervisor)

University of Sheffield.

S.3 Qualtrics Information for those who do not Consent to Participate

This is the page that will be shown on Qualtrics if a person does not consent to participating in the study:

Thank you for taking the time to contact us and read this information. As you have not consented to participate in this study at this time, you will receive no further contact from us.

If you do wish to participate in the study and came to this page by accident or through a computer error, please contact us on the below email address and let us know:

Hmwigglesworth1@sheffield.ac.uk

Thank you, and best wishes,

Helen Wigglesworth (Trainee Clinical Psychologist) and Abi Millings (Lecturer in Psychology and Project Supervisor)

University of Sheffield.

S.4 Welcome Email

Please find below the email that is to be sent when participants consented to participating in the study.

Hello,

Thank you for agreeing to participate in this sling use and maternal wellbeing research project.

When you have given birth, and when you feel able, please click on this link below to complete your first set of questionnaires:

INSERT LINK

These questionnaires should take about **DURATION** minutes to complete in total.

We will send you a text reminder to complete these questionnaires around the time of your due date. If you would prefer to receive an email reminder, or would prefer not to receive a reminder at all, please reply to this email and let us know.

Again, thank you for your participation, and please feel free to reply to this email with any questions that you may have,

Helen Wigglesworth (Trainee Clinical Psychologist) and Abi Millings (Lecturer in Psychology and Project Supervisor)

University of Sheffield.

S.5 Reminder Email/Text

Content that may be sent in either an email or text (depending on the participant's stated preference - email if not known) as a reminder for the participant to complete either their T1, T2 or T3 questionnaires.

Hello,

This **[email/text]** is being sent to you as a reminder that you are due to complete your **[first/second/final]** set of questionnaires. Please complete these questionnaires as soon as possible after receiving this **[email/text]**.

Please click on the link below to complete the questionnaires:

Insert link

Thank you for participating in the sling provision and maternal wellbeing research project.

You are free to withdraw from this study at any point. If you no longer wish to participate, please email us using the contact details below to let us know, and we will withdraw your data.

[Also, if you would like to opt out of these text reminders, and be contacted by email only, please let us know.] - For text reminders only.

Please do not hesitate to contact a member of the research team if you have any questions or concerns:

Principal Investigator: Helen Wigglesworth, Trainee Clinical Psychologist, hmwigglesworth1@sheffield.ac.uk

Project Supervisor: Dr Abigail Millings, a.millings@sheffield.ac.uk

Best wishes,

Helen and Abi,

University of Sheffield

S.6. Qualtrics Intro - All Time-points

Introduction text that will be presented on Qualtrics at T1, T2, and T3. This is a template as the details will change slightly based on the time-point (this template applies to T1).

Hello,

Thank you for agreeing to participate in this study.

You will now be asked to complete a series of questionnaires. These should take around **DURATION** to complete. These questionnaires will ask you about your mood, general wellbeing and experiences of parenting so far. You will also be asked demographic questions (e.g. age, ethnicity, postcode, education). Please read the instructions for each questionnaire carefully, as each questionnaire may ask you about a different time period (e.g. over the last week or over the last year etc.), or may give you different response options.

We do not anticipate that these questionnaires will cause you undue distress, but of course, talking about our mood, wellbeing or experiences of parenting so far can be an emotional process. We encourage you to take breaks if you need to. If you experience any difficult feelings while completing these questionnaires, and feel that you require support, please contact your GP.

Please remember that you can stop and withdraw your data at any point if you no longer wish to participate in this study.

Contact details:

Principal Investigator: Helen Wigglesworth, Trainee Clinical Psychologist, hmwigglesworth1@sheffield.ac.uk

Project Supervisor: Dr Abigail Millings, a.millings@sheffield.ac.ukBest wishes and thank you again,

Helen and Abi.

University of Sheffield

S.7 Qualtrics End Information - T1 - Intervention Group

Information to be presented on Qualtrics at the end of the T1 battery of measures for the intervention group only. This will be sent in an email or text (depending on the participant's stated preference) as well, as an extra reminder.

Thank you for completing these questionnaires.

You are now invited to attend a session at the Sheffield Sling Surgery.

Please attend a session as soon as is convenient for you (if possible, within the next two weeks).

You will now be sent an email with a link to upcoming Sling Surgery drop-in sessions.

Using this link, please decide which Sling Surgery drop-in session you would like to attend.

Please then respond to the email (hmwigglesworth1@sheffield.ac.uk) letting us know which session you intend to go to. This is so that we can make sure that a sling is available for you to hire for free for the duration of the study.

When you attend, please let a member of staff know that you are a participant in this study so that you are not charged for your sling hire.

When you attend this drop-in session you will receive training and support around how to safely use a baby sling, and you will be given a sling to hire free of charge. Please feel free to also access any of the other services offered by the Sling Surgery.

You will receive an email or text, depending on your preference, letting you know when you are due to complete your next set of questionnaires in 6 weeks' time. If you have not told us whether you prefer to communicate with us via email or text, please let us know by emailing the following:

Principal Investigator: Helen Wigglesworth, Trainee Clinical Psychologist, hmwigglesworth1@sheffield.ac.uk

Thank you again for participating,

Best wishes,

Helen and Abi.

University of Sheffield

S.8 "Next Steps" Email to be sent to Intervention Participants asking them to Book a Sling Surgery Session to attend

Hi,

Thank you for completing this first set of questionnaires.

You are now invited to attend a session at the Sheffield Sling Surgery.

Please attend a session as soon as is convenient for you (if possible, within the next two weeks).

Below is a link showing upcoming Sling Surgery drop-in sessions:

https://www.sheffieldslingsurgery.co.uk/calendar/

Using this link, please decide which Sling Surgery drop-in session you would like to attend.

Please respond to this email (hmwigglesworth1@sheffield.ac.uk) letting us know which session you intend to go to. This is so that we can make sure that a sling is available for you to hire for free for the duration of the study.

When you attend, please let a member of staff know that you are a participant in this study so that you are not charged for your sling hire.

When you attend this drop-in session you will receive training and support around how to safely use a baby sling, and you will be given a sling to hire free of charge. Please feel free to also access any of the other services offered by the Sling Surgery.

You will receive an email or text, depending on your preference, letting you know when you are due to complete your next set of questionnaires in 6 weeks' time. If you have not told us whether you prefer to communicate with us via email or text, please let us know by emailing the following:

Principal Investigator: Helen Wigglesworth, Trainee Clinical Psychologist, hmwigglesworth1@sheffield.ac.uk

Thank you again for participating,

Best wishes,

Helen and Abi.

University of Sheffield

S.9 Qualtrics End Information - T1 - Control Group

Information to be presented on Qualtrics at the end of the T1 battery of measures for the control group only.

Thank you for completing these questionnaires.

You will receive an email or text, depending on your preference, letting you know when you are due to complete your next set of questionnaires in 6 weeks' time. If you have not told us whether you prefer to communicate with us via email or text, please let us know by emailing the following:

hmwigglesworth1@sheffield.ac.uk

Thank you again for participating,

Best wishes,

Helen and Abi,

University of Sheffield.

S.10 Qualtrics End Information - T2 - Intervention Group

Information to be presented on Qualtrics at the end of the T2 battery of measures for the intervention group only.

Thank you for completing these questionnaires.

You will receive an email or text, depending on your preference, letting you know when you are due to complete your next set of questionnaires in 6 weeks' time. If you have not told us whether you prefer to communicate with us via email or text, please let us know by emailing the following:

hmwigglesworth1@sheffield.ac.uk

We encourage you to keep using your sling regularly between now and your next set of questionnaires. If you have any questions or concerns about your sling, please do not hesitate to contact the Sheffield Sling Surgery using the email below. If you contact the Sling Surgery, please remember to state that you are a participant in this study:

info@sheffieldslingsurgery.co.uk

Thank you again for participating,
Best wishes,
Helen and Abi,
University of Sheffield

S.11 Qualtrics End Information - T2 - Control Group

Information to be presented on Qualtrics at the end of the T2 battery of measures for the control group only.

Thank you for completing these questionnaires.

You will receive an email or text, depending on your preference, letting you know when you are due to complete your next set of questionnaires in 6 weeks' time. If you have not told us whether you prefer to communicate with us via email or text, please let us know by emailing the following:

Principal Investigator: Helen Wigglesworth, Trainee Clinical Psychologist,
hmwigglesworth1@sheffield.ac.uk

Thank you again for participating,
Best wishes,
Helen and Abi,

University of Sheffield.

S.12 Qualtrics End Information - T3 - Intervention Group

Information to be presented on Qualtrics at the end of the T3 battery of measures for the intervention group only.

Thank you for completing these questionnaires.

You have now completed all three sets of questionnaires. You will be sent an email, debriefing you about this study.

Please now return your hire sling to the Sheffield Sling Surgery as soon as possible.

The Sling Surgery can support you in either continuing to hire this sling, with the usual hire charge applied, or in hiring or purchasing another sling.

If at this point you have any questions or concerns about having participated in this study, please do not hesitate to contact us using the details below:

Principal Investigator: Helen Wigglesworth, Trainee Clinical Psychologist, hmwigglesworth1@sheffield.ac.uk

Project Supervisor: Dr Abigail Millings, a.millings@sheffield.ac.uk

Also, please let us know if you would to be sent (via email) a summary of the results of this study when they are available.

Thank you again for participating,

Best wishes,
Helen and Abi,
University of Sheffield.

S.13 Qualtrics End Information - T3 - Control Group

Information to be presented on Qualtrics at the end of the T3 battery of measures for the control group only.

Thank you for completing these questionnaires.

You have now completed all three sets of questionnaires. You will be sent an email, debriefing you about this study.

You are now invited to attend a session at the Sheffield Sling Surgery.

Please attend a session as soon as is convenient for you (if possible, within the next two weeks).

You will now be sent an email with a link to upcoming Sling Surgery drop-in sessions.

Using this link, please decide which Sling Surgery drop-in session you would like to attend.

Please then respond to the email (hmwigglesworth1@sheffield.ac.uk) letting us know which session you intend to go to. This is so that we can make sure that a sling is available for you to hire for free.

When you attend, please let a member of staff know that you are a participant in this study so that you are not charged for your sling hire.

When you attend this **drop-in session** you will receive training and support around how to safely use a baby sling, and you will be given a sling to hire free of charge **for 4 weeks**. **Please return your sling after this time**. You will be given the opportunity to either re-hire it out at the usual rate, or hire out or purchase another sling.

You will receive an email or text, depending on your preference, letting you know when you are due to return your sling. If you have not told us whether you prefer to communicate with us via email or text, please let us know by contacting the following:

Principal Investigator: Helen Wigglesworth, Trainee Clinical Psychologist, hmwigglesworth1@sheffield.ac.uk

Also, please let us know if you would to be sent (via email) a summary of the results of this study when they are available.

Thank you again for participating.

Best wishes,

Helen and Abi,

University of Sheffield.

S.14 "Next Steps"/Debrief Email to be sent to Control Participants asking them to Book a Sling Surgery Session to attend

Hi,

Thank you for completing all three sets of questionnaires. **Please find attached to this email a debrief sheet**, which offers further information about the purpose of this study and what happens next.

You are now invited to attend a session at the Sheffield Sling Surgery.

Please attend a session as soon as is convenient for you (if possible, within the next two weeks).

Please find below a link to information about upcoming Sling Surgery sessions:

https://www.sheffieldslingsurgery.co.uk/calendar/

Using this link, please decide which Sling Surgery drop-in session you would like to attend.

Please then respond to the email (hmwigglesworth1@sheffield.ac.uk) letting us know which session you intend to go to. This is so that we can make sure that a sling is available for you to hire for free.

When you attend, please let a member of staff know that you are a participant in this study so that you are not charged for your sling hire.

When you attend this **drop-in session** you will receive training and support around how to safely use a baby sling, and you will be given a sling to hire free of charge **for 4 weeks**. **Please return your sling after this time**. You will be given the opportunity to either re-hire it out at the usual rate, or hire out or purchase another sling.

You will receive an email or text, depending on your preference, letting you know when you are due to return your sling. If you have not told us whether you prefer to communicate with us via email or text, please let us know by contacting the following:

Principal Investigator: Helen Wigglesworth, Trainee Clinical Psychologist, hmwigglesworth1@sheffield.ac.uk

Also, please let us know if you would to be sent (via email) a summary of the results of this study when they are available.

Thank you again for participating.

Best wishes,

Helen and Abi,

University of Sheffield.

S.15 Debrief Email to be Sent to Intervention Participants

Hi,

Thank you for completing these questionnaires.

You have now completed all three sets of questionnaires. **Please find attached to this email a debrief sheet**, which offers further information about the purpose of this study and what happens next.

Please now return your hire sling to the Sheffield Sling Surgery as soon as possible.

The Sling Surgery can support you in either continuing to hire this sling, with the usual hire charge applied, or in hiring or purchasing another sling.

If at this point you have any questions or concerns about having participated in this study, please do not hesitate to contact us using the details below:

Principal Investigator: Helen Wigglesworth, Trainee Clinical Psychologist, hmwigglesworth1@sheffield.ac.uk

Project Supervisor: Dr Abigail Millings, a.millings@sheffield.ac.uk

Also, please let us know if you would to be sent (via email) a summary of the results of this study when they are available.

Thank you again for participating,

Best wishes,
Helen and Abi,
University of Sheffield.

Appendix T Drop-In Session Checklist

Checklist to be used by Sling Surgery staff when providing sling training and information to study participants. This is based on the Sling Surgery's current standard session procedures.

Session Checklist

1. Greet and state your name
2. State whether you are a peer supporter or a consultant
3. Ask the reason for the person wanting a sling and what has brought the person to the sling surgery.
4. Ask whether there are any special circumstances that it would be helpful to be aware of (e.g. physical disability (parent or infant), dyspraxia, a particular budget).
5. Ask how old the baby is.
6. Check the parent's body shape.
7. Select one or two possible slings to offer (from stretchy or close caboo types, or one type of buckle carrier).
8. Demonstrate sling use, using a demo doll.
9. Ask the parent to practice wearing the sling using a demo doll.
10. Offer the parent the opportunity to practice wearing the sling with their baby.
11. While the parent is practicing with either their baby or the demo doll, offer sling safety instructions (as stated in the safety information leaflet).
12. If the parent chooses to hire the sling, direct them to the shop so that they can complete the relevant paperwork.

Appendix U Sling Surgery Materials and Emails

U.1 Sling safety leaflet provided to participants during their sling training.

[Copyrighted material removed. Further information available from authors on request.]

U.2. Sling Surgery Emails

Examples of the emails that participants will receive when first hiring their sling from, or returning their sling to, the Sling Surgery as part of the study.

U.2a Email address confirmation email.

Sheffield Sling Surgery

Hello, Jane

To fully activate your account with Sheffield Sling Surgery and Library, please click on the link below:

Confirm email address

Janedoe1@sheffield.ac.uk has an account with Sheffield Sling Surgery. Your username: JDoe123.

Many thanks

Rob and Rosie and the team

A note about privacy:

When you become a customer of the Sheffield Sling Surgery and Sling Spot we invite you to be a part of a community. We would like to reassure you that your privacy is important. We will never sell your data, all details about you are stored securely and we only send you information directly related to your purchase, hire, consult or workshop. More information

regarding our privacy policy is available on the website at www.sheffieldslingsurgery.co.uk/privacy

---

Sheffield Sling Surgery
The Snug, 71, Leadmill Road, Sheffield, S1 4SE, United Kingdom

Monday	10:00 - 14:00 (BST)
Tuesday	10:00 - 14:00 (BST)
Wednesday	10:00 - 14:00 (BST)
Thursday	10:00 - 14:00 (BST)
Friday	10:00 - 14:00 (BST)
Saturday	10:00 - 13:00 (BST)
Sunday	Closed

Please check our website or our Facebook page for our library drop in dates each week.

Our sister service, Sling Spot (next door to the Snug), is open every day for returns and fast track hires.

Powered by myTurn

U.2b Welcome email.

Sheffield Sling Surgery

Hello Helen

Welcome to Sheffield Sling Surgery and Library! We are happy to be to helping you on your carrying journey, do get in touch if you have any questions.

Please keep this email for your records. Your account information is as follows:

Username: JDoe123
Your Email: Janedoe1@sheffield.ac.uk
Name: Jane Doe

Please confirm your email address

https://sheffieldslingsurgery.myturn.com/library/

Edit Your Account

Your password was automatically generated and is stored securely in our database. If you wish to access your account on the site itself, you can reset your password to one of your own choosing on the login page.

Thank you for registering.

Rob and Rosie and the team

A note about privacy:

When you become a customer of the Sheffield Sling Surgery and Sling Spot we invite you to be a part of a community. We would like to reassure you that your privacy is important. We will never sell your data, all details about you are stored securely and we only send you information directly related to your purchase, hire, consult or workshop. More information regarding our privacy policy is available on the website at www.sheffieldslingsurgery.co.uk/privacy

---

Sheffield Sling Surgery
The Snug, 71, Leadmill Road, Sheffield, S1 4SE, United Kingdom

---

U2.c Hiring/returning email.

Sheffield Sling Surgery

Thank you for hiring (or returning) a carrier from the Sheffield Sling Surgery and Library! This is your receipt email for the transaction.

If you have **returned** today, many thanks. We hope to see you again soon!

If you **hired** today, please click on the blue underlined link to the carrier you have hired, it contains all the user information you may need. You may also find helpful links here http://www.sheffieldslingsurgery.co.uk/personal-support/using-your-carrier/

If you would like to return/swap your carrier, the upcoming Surgery drop ins can be found here

www.sheffieldslingsurgery.co.uk/calendar or www.facebook.com/sheffieldslingsurgery/events

Alternatively you can bring it back to the Sling Spot shop (next door to the Snug, 73 Leadmill Road, S1 4SE) on the day it is due back if there is no library session that day.

If you are enjoying your carrier and would like your own, we stock most major brands at the Sling Spot and offer local discounts.

More information can be found on our website (www.sheffieldslingsurgery.co.uk)) and in the Virtual Sling Surgery, our online facebook support/chat group (www.facebook.com/groups/virtualslingsurgery) Please do join, it is a great way to keep up with local sling news and make friends.

28/07/2018

Transaction details (reference #XXXXXX)

Name: Jane Doe
Email: Janedoe1@Sheffield.ac.uk
Telephone: 07123456789

You have checked out this item.

Summary

**Transactions**
Checkout: (Close Caboo Anniversary Edition)

Due Dates

Item	Due Date
Close Caboo Anniversary Edition	04/08/2018

View in browser

Thank you for using the Sheffield Sling Surgery and Library services.

We hope to see you again soon.

Rob and Rosie and the team

A note about privacy:

When you become a customer of the Sheffield Sling Surgery and Sling Spot we invite you to be a part of a community. We would like to reassure you that your privacy is important. We will never sell your data, all details about you are stored securely and we only send you information directly related to your purchase, hire, consult or workshop. More information regarding our privacy policy is available on the website at www.sheffieldslingsurgery.co.uk/privacy

---

Sheffield Sling Surgery
The Snug, 71, Leadmill Road, Sheffield, S1 4SE, United Kingdom

Monday	10:00 - 14:00 (BST)
Tuesday	10:00 - 14:00 (BST)
Wednesday	10:00 - 14:00 (BST)
Thursday	10:00 - 14:00 (BST)
Friday	10:00 - 14:00 (BST)
Saturday	10:00 - 13:00 (BST)
Sunday	Closed

Please check our website or our Facebook page for our library drop in dates each week.

Our sister service, Sling Spot (next door to the Snug), is open every day for returns and fast track hires.

Appendix V Participant Debrief Sheet

21/02/19 Version 1

Sling Provision and Maternal Wellbeing Study

Participant Debrief Sheet

Research Project Title:

Evaluating the Impact of Sling Provision and Training upon Maternal Wellbeing and Parenting: A Randomised Feasibility Trial

Researcher:

Helen Wigglesworth, Trainee Clinical Psychologist

Thank you for taking part in this study.

What were the aims of this study?

This study aimed to investigate whether the provision of a sling, and also the provision of training in the safe use of a sling and how to access peer support from other sling users, may have an impact on the mental health, wellbeing or parenting experiences of women who have recently given birth.

We also examined whether differences in aspects of personality relevant to close relationships, "attachment style", played a part in the impact of sling use on mental health, wellbeing, or parenting experience.

This was a feasibility study. This means that the main aim of this study was to see whether a study like this is even possible to conduct, as the effect of sling-use on maternal mental health is a new area of research.

How was this done?

To do this, you were randomly allocated to one of two groups. If you were in the intervention group, you will have been given a baby sling at the beginning of the study, when your baby was 0-6 weeks old. You will have been asked to complete questionnaires three times; at the start of the study, after 6 weeks and after 12 weeks. These questionnaires included measures of postnatal depression, wellbeing, parental attachment style, and various aspects of the parenting experience (e.g. caregiving experience, sense of competency, social support).

If you were in the control group, you will have completed the same questionnaires, at the same times, but will have not been given a baby sling until after you had completed the final questionnaire at 12 weeks. This is so that we can look at whether there are any differences in the questionnaire scores between the two groups, and whether these differences change over time.

Talking about our mood, wellbeing or experiences of parenting so far can be an emotional process. If you experienced any difficult feelings while completing these questionnaires, and feel that you require support, please contact your GP or health visitor.

What will happen to the data collected?

Any questionnaire data collected from you has been anonymised. This data is stored securely and is only available to members of the research team, including staff from the Sheffield Sling Surgery. Sheffield Sling Surgery will retain your contact details until after you have returned the sling you received under a free hire arrangement as part of this project, at which point, you can request that this information be destroyed. The University of Sheffield will destroy your contact details after sending you this debriefing sheet. Your contact details are not linked in any way to the questionnaire data you have provided, so you cannot be identified within the aggregated set of responses.

What will happen to the results of the research?

As the study is part of my doctoral course in Clinical Psychology, it will be submitted to the University for marking. It may be that in the future the findings of this study are published in a relevant journal or presented at a conference. Participants will not be identifiable within any of these publications.

A brief report of the findings will be sent to interested participants. To register your interest, please email me (Helen Wigglesworth, Principal Investigator) using the following email address:

Hmwigglesworth1@sheffield.ac.uk

Due to the nature of this research it is very likely that other researchers may find the data collected to be useful in answering future research questions. Thus anonymous data from this study may be made available to other researchers after this current research is completed.

Who has ethically reviewed the project?

This project has been ethically approved via the University of Sheffield's Ethics Review Procedure, as administered by the Department of Clinical Psychology. The University's Research Ethics Committee monitors the application and delivery of the University's Ethics Review Procedure across the University.

What if I have any questions or concerns, or want to withdraw my data?

If you have any questions, concerns, or complaints about any aspect of this study, please do not hesitate to contact either myself or my project supervisor (please see below):

**Principal Investigator:**	**Project Supervisor:**
Helen Wigglesworth, Trainee Clinical Psychologist<br>hmwigglesworth1@sheffield.ac.uk	Dr Abigail Millings<br>a.millings@sheffield.ac.uk

Clinical Psychology Unit,<br>Department of Psychology,<br>University of Sheffield,<br>Floor F, Cathedral Court,<br>1 Vicar Lane,<br>Sheffield, S1 2LT	Lecturer in Psychology, Postgraduate Tutor, and PG Careers Contact,<br>Department of Psychology,<br>University of Sheffield,<br>Floor D, Cathedral Court,<br>1 Vicar Lane,<br>Sheffield,<br>S1 2LT<br>Tel: 01142226525

If you have made a complaint, but feel that your complaint has not been handled to your satisfaction, or that you wish to contact a person external to the project, please do not hesitate to contact our Head of Department:

Professor Glenn Waller,

g.waller@sheffield.ac.uk

Head of Psychology Department

Department of Psychology
University of Sheffield
Floor D, Cathedral Court
1 Vicar Lane
Sheffield
S1 2LT

The Head of Department will then be able to escalate the complaint through the appropriate channels.

If your complaint relates to how your personal data has been handled, then further information about raising this type of complaint may be found in the University's Privacy Notice: https://www.sheffield.ac.uk/govern/data-protection/privacy/general.

Thank you again for your willingness to participate in this study. It is much appreciated. And thank you for taking the time to read this debrief sheet.

Appendix W CONSORT 2010 Flow Diagram Template

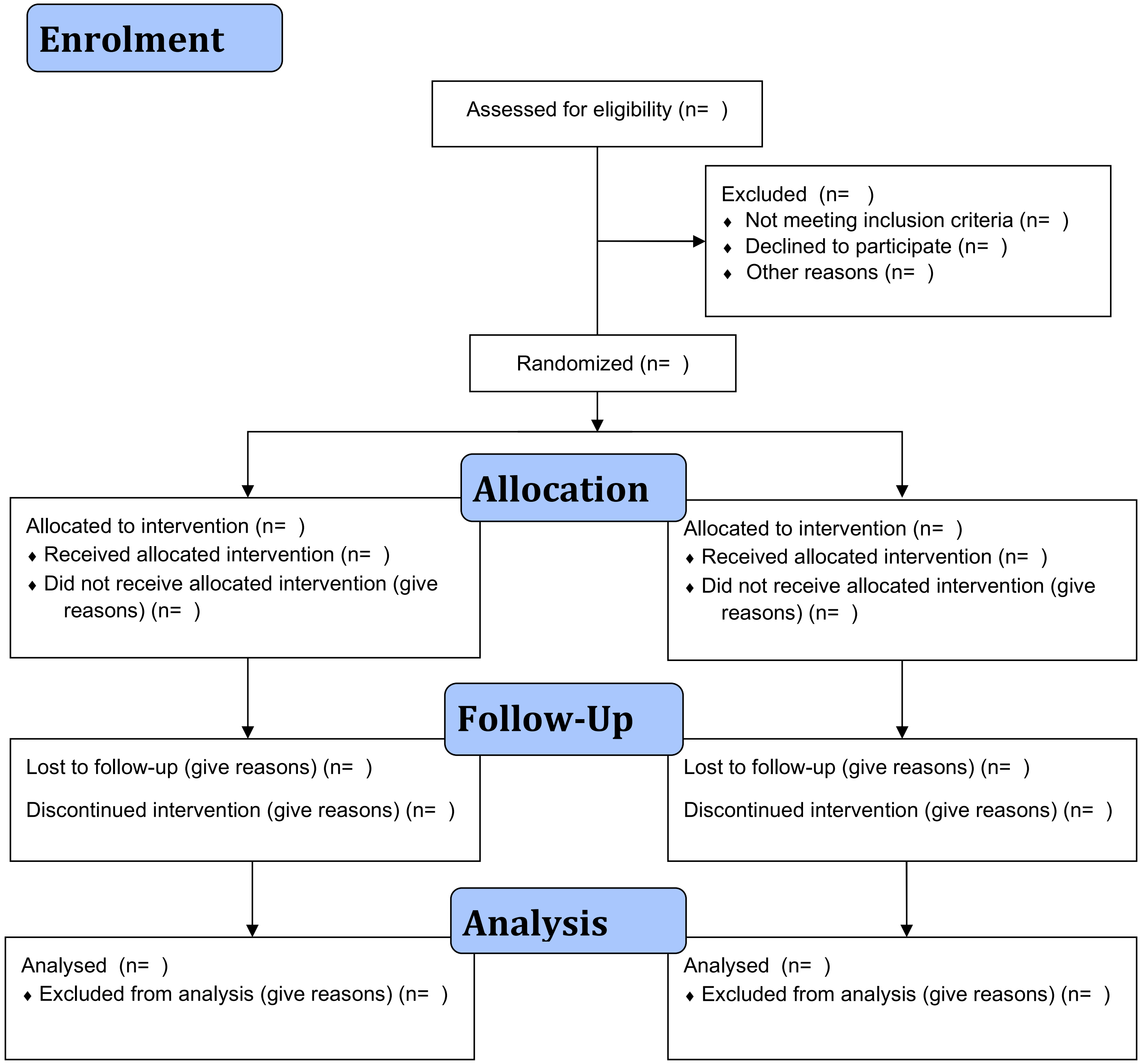

Appendix X Timetable of Work

			2018	2019												2020				
			December	January	February	March	April	May	June	July	August	September	October	November	December	January	February	March	April	May
Task Name	***Total Duration***	***Who is Responsible?***																		
Application for ethical approval	28/12/2018	HW																		
Recruitment	5.5 Months	HW																		
**Data Collection & Intervention**																				
T1 (baseline) measure completion & intervention participants attend sling training.	6.5 months	HW/RK																		
Intervention participants receiving free sling hire	8.5 months																			
T2 measure completion	5.5 months	HW																		
T1/T2 data analysis and comparison	6 months	HW																		
T3 measure completion & control participants attend sling sessions.	6.5 months	HW/RK																		
**Full Data Analysis & Waitlist Control Receive Intervention**																				
Control participants receiving free sling hire	6.5 months																			
Analysis of all data	2 months	HW																		
Write up results and conclusions, and receive feedback.	4 months	HW/AM																		
Thesis submission	20/05/2018	HW																		
HW - Helen Wigglesworth, Trainee Clinical Psychologist	*AM - Dr. Abigail Millings, Project Supervisor*	*RK - Dr. Rosie Knowles, Sheffield Sling Surgery*																		

Appendix Y Project Costing

Research Proposal Costing Form

Name of Trainee ....Helen Wigglesworth...................................

Date of costing………13/08/18……………………………………….

Please provide itemised details of all costings under the following headings – refer to costing guidelines

**Stationery** (envelopes, labels, paper etc)<br><br>20 x A5 Manilla Envelopes	£1.00
subtotal	£1.00

Postage Include:<br>- cost of outward postage (NB: varies according to size/weight – check with Royal Mail) e.g. 200 2nd class letters @ [amount each] for outward mailing<br>- Freepost envelopes, plus cost of return postage for the number of estimated returns<br><br>20 1st Class signed for letters @ £1.77 each for outward mailing.<br><br>*This more expensive postage option has been selected for ethical reasons, as it is for letters that will be sent in response to participants who have scored as likely to be suffering from postnatal depression, recommending that they contact their GP/Midwife/Health Visitor.*	£35.40
subtotal	£35.40

Trainee name ....Helen Wigglesworth..

Photocopying / Duplicating / Printing<br>e.g. 200 x 4 page questionnaires in booklet form [amount]<br><br>*(Quotes are from University Print and Design Solutions Service - myprint service)*<br><br>250 x A4 Recruitment posters.<br><br>250 x A5 Double sided recruitment leaflets.<br><br>*Printing using the Clinical Psychology Unit Photocopier (Cathedral Court)*<br><br>20 x A4 One-sided letter	 <br><br><br><br>£42.50<br><br>£30.00<br><br><br><br>£0.60
subtotal	£73.10

Travel costs<br>Itemise journeys – number of and reason for journeys<br>For mileage, itemise miles and deduct normal home-work mileage	0
subtotal	0

Tests / Equipment<br>Qualtrics Survey Software<br><br>Pay as you go SIM card<br>Budget for pay as you go credit (for 6 months)	 <br>£0<br><br>£10<br>£70
subtotal	£80

Transcribing<br><br>Calculate at current UoS worker rate for transcribing	0
subtotal	0

Miscellaneous costs	0
subtotal	0
**Total estimated cost for your research project**:	£189.50
